# Observational, causal relationship and shared genetic basis between cholelithiasis and gastroesophageal reflux disease: evidence from a cohort study and comprehensive genetic analysis

**DOI:** 10.1093/gigascience/giaf023

**Published:** 2025-03-26

**Authors:** Yanlin Lyu, Shuangshuang Tong, Wentao Huang, Yuying Ma, Ruijie Zeng, Rui Jiang, Ruibang Luo, Felix W Leung, Qizhou Lian, Weihong Sha, Hao Chen

**Affiliations:** Department of Gastroenterology, Guangdong Provincial People’s Hospital (Guangdong Academy of Medical Sciences), Southern Medical University, Guangzhou 510080, China; The Second School of Clinical Medicine, Southern Medical University, Guangzhou 510515, China; Shantou University Medical College, Shantou University, Shantou 515041, China; Department of Gastroenterology, Guangdong Provincial People’s Hospital (Guangdong Academy of Medical Sciences), Southern Medical University, Guangzhou 510080, China; Shantou University Medical College, Shantou University, Shantou 515041, China; Department of Gastroenterology, Guangdong Provincial People’s Hospital (Guangdong Academy of Medical Sciences), Southern Medical University, Guangzhou 510080, China; The Second School of Clinical Medicine, Southern Medical University, Guangzhou 510515, China; Department of Gastroenterology, Guangdong Provincial People’s Hospital (Guangdong Academy of Medical Sciences), Southern Medical University, Guangzhou 510080, China; The Second School of Clinical Medicine, Southern Medical University, Guangzhou 510515, China; Department of Gastroenterology, Guangdong Provincial People’s Hospital (Guangdong Academy of Medical Sciences), Southern Medical University, Guangzhou 510080, China; Shantou University Medical College, Shantou University, Shantou 515041, China; Department of Gastroenterology, Guangdong Provincial People’s Hospital (Guangdong Academy of Medical Sciences), Southern Medical University, Guangzhou 510080, China; School of Medicine, South China University of Technology, Guangzhou 510006, China; Department of Computer Science, The University of Hong Kong, Hong Kong 999077, China; Sepulveda Ambulatory Care Center, VA Greater Los Angeles Healthcare System, Los Angeles, CA 91343, USA; University of California Los Angeles David Geffen School of Medicine, Los Angeles, CA 90095, USA; Faculty of Synthetic Biology, Shenzhen Institute of Advanced Technology, Chinese Academy of Sciences, Shenzhen 518055, China; Cord Blood Bank, Guangzhou Institute of Eugenics and Perinatology, Guangzhou Women and Children’s Medical Center, Guangzhou Medical University, Guangzhou 510623, China; State Key Laboratory of Pharmaceutical Biotechnology, The University of Hong Kong, Hong Kong 999077, China; Department of Gastroenterology, Guangdong Provincial People’s Hospital (Guangdong Academy of Medical Sciences), Southern Medical University, Guangzhou 510080, China; The Second School of Clinical Medicine, Southern Medical University, Guangzhou 510515, China; Shantou University Medical College, Shantou University, Shantou 515041, China; School of Medicine, South China University of Technology, Guangzhou 510006, China; Department of Gastroenterology, Guangdong Provincial People’s Hospital (Guangdong Academy of Medical Sciences), Southern Medical University, Guangzhou 510080, China; The Second School of Clinical Medicine, Southern Medical University, Guangzhou 510515, China; Shantou University Medical College, Shantou University, Shantou 515041, China; School of Medicine, South China University of Technology, Guangzhou 510006, China

**Keywords:** cholelithiasis, gastroesophageal reflux disease, cohort study, Mendelian randomization, genetic analyses, causal association, shared genetic basis

## Abstract

**Objective:**

Cholelithiasis and gastroesophageal reflux disease (GERD) contribute to significant health concerns. We aimed to investigate the potential observational, causal, and genetic relationships between cholelithiasis and GERD.

**Design:**

The observational correlations were assessed based on the prospective cohort study from UK Biobank. Then, by leveraging the genome-wide summary statistics of cholelithiasis (*N* = 334,277) and GERD (*N* = 332,601), the bidirectional causal associations were evaluated using Mendelian randomization (MR) analysis. Subsequently, a series of genetic analyses was used to assess the genetic correlation, shared loci, and genes between cholelithiasis and GERD.

**Results:**

The prospective cohort analyses revealed a significantly increased risk of GERD in individuals with cholelithiasis (hazard ratio [HR] = 1.99; 95% confidence interval [CI], 1.89–2.10) and a higher risk of cholelithiasis among patients with GERD (HR = 2.30; 95% CI, 2.18–2.44). The MR study indicated the causal effect of genetic liability to cholelithiasis on the incidence of GERD (odds ratio [OR] = 1.08; 95% CI, 1.05–1.11) and the causal effect of genetic predicted GERD on cholelithiasis (OR = 1.15; 95% CI, 1.02–1.31). In addition, cholelithiasis and GERD exhibited a strong genetic association. Cross-trait meta-analyses identified 5 novel independent loci shared between cholelithiasis and GERD. Three shared genes, including *SUN2, CBY1*, and *JOSD1*, were further identified as novel risk genes.

**Conclusion:**

The elucidation of the shared genetic basis underlying the phenotypic relationship of these 2 complex phenotypes offers new insights into the intrinsic linkage between cholelithiasis and GERD, providing a novel research direction for future therapeutic strategy and risk prediction.

## Graphical Abstract

**Graphical Abstract:**
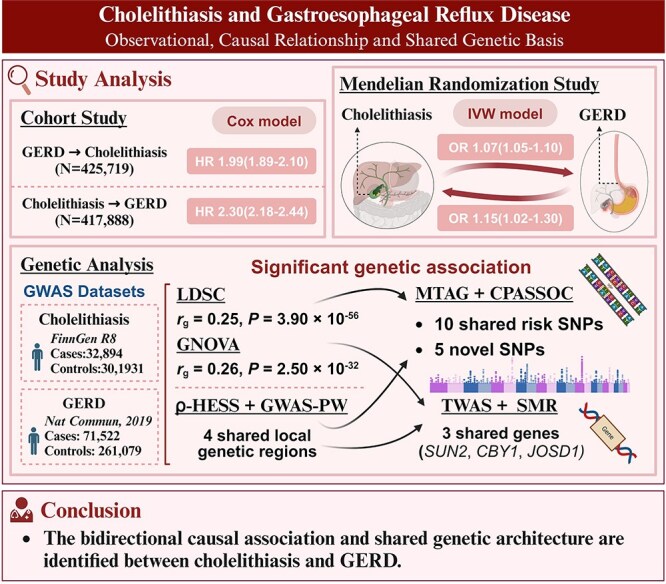

## Introduction

Cholelithiasis, a condition characterized by lithic deposits of either cholesterol or bilirubin in the gallbladder or the bile ducts, is one of the most prevalent digestive disorders, imposing a significant socioeconomic burden [1]. Cholelithiasis affects nearly 20% of the adult population worldwide, with a continuously rising incidence rate [1, 2]. The development of cholelithiasis involves intricate mechanisms, encompassing genetic and environmental factors and their interactions. [1, 3] Gastrointestinal defects in patients with cholelithiasis have raised widespread concerns and require further exploration [1, 4].

Gastroesophageal reflux disease (GERD) is a common gastrointestinal disorder typically characterized by recurrent heartburn and regurgitation [5, 6]. This condition could pose a substantial public health challenge, owing to its association with a spectrum of subsequent severe complications, including Barrett’s esophagus, esophageal stenosis, and esophageal adenocarcinoma [7]. Therefore, early identification and vigilant monitoring of individuals at high risk for GERD can facilitate timely intervention, potentially mitigating the severity of the disease and decreasing the risk of GERD and GERD-related complications.

Several studies have investigated the correlation between cholelithiasis and the risk of GERD [8–11]. Nonetheless, the existing findings have been inconsistent and insufficient, lacking support from prospective studies. For instance, a retrospective, observational study involving 1,381,004 individuals with gallstone disease found that 40% of the patients had concurrent GERD [11]. On the contrary, a case-control study, comprising 790 cases and 407 controls demonstrated no associations between the presence of cholelithiasis and GERD [9]. Most of the previous studies are outdated and statistically underpowered due to small sample sizes. In addition, these observational studies are prone to some inevitable defects such as potential reverse causality and confounding [12]. The causal association between cholelithiasis and GERD remains obscure. Therefore, large datasets and updated methodologies are warranted to disentangle the conflicting relationship between them and to further reveal the underlying genetic underpinnings.

**Figure 1:**
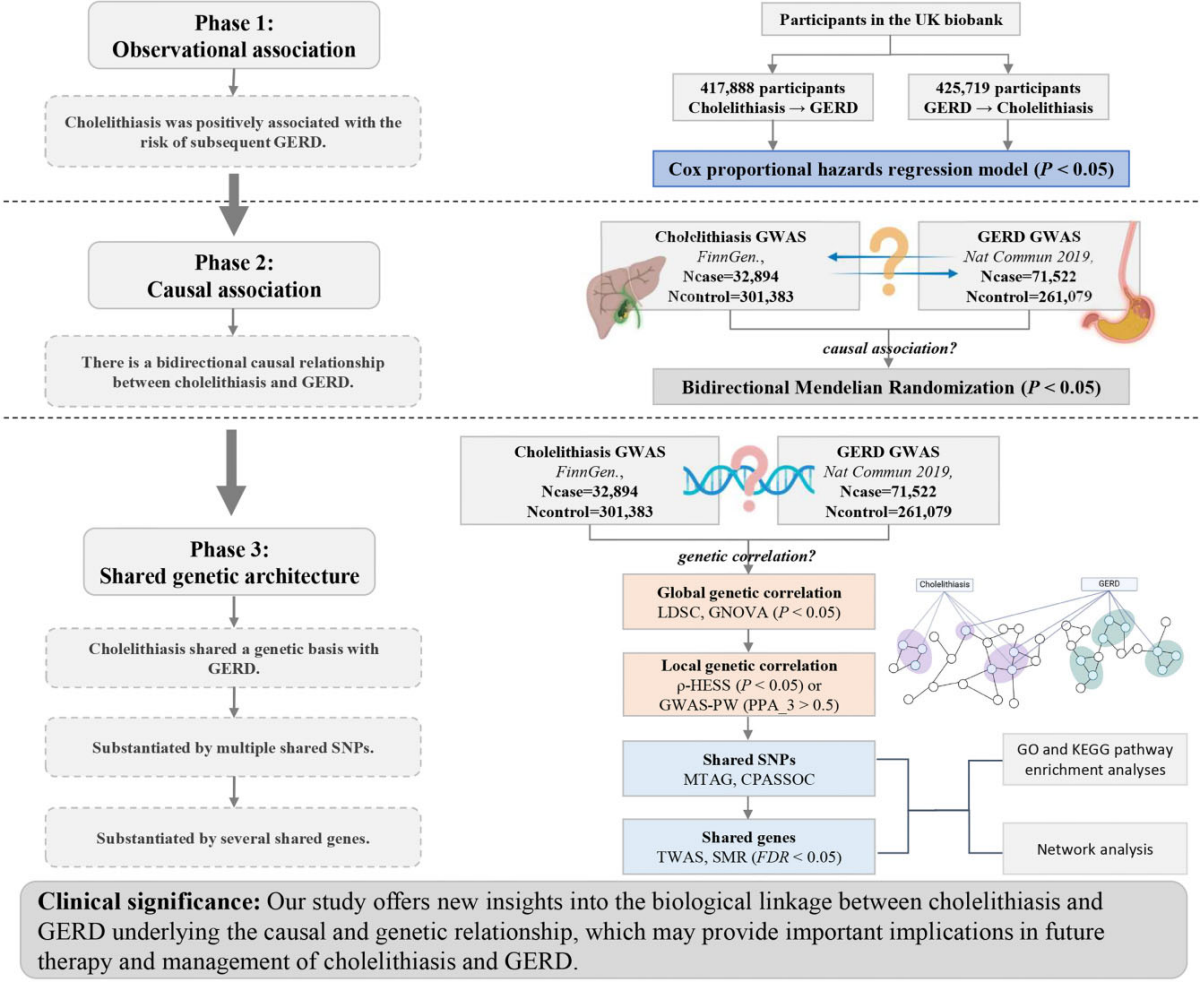
Flowchart of the overall study design. First, we assessed the phenotypic correlations between cholelithiasis and GERD based on the prospective cohort data from the UK Biobank. Second, we conducted a bidirectional 2-sample Mendelian randomization analysis to investigate the causality by using large-scale GWAS data. Third, we utilized a variety of approaches to dissect the genetic correlations and shared genetic architecture. LDSC and GNOVA methods were applied to detect the global genetic correlation. ρ-HESS and GWAS-PW methods were used to further explore the local genetic correlation. Then, MTAG and CPASSOC methods were employed to find the shared-risk SNPs. Finally, TWAS and SMR methods were utilized to study the shared genes between cholelithiasis and GERD. CPASSOC: cross-phenotype association test; FDR: false discovery rate; GERD: gastroesophageal reflux disease; GNOVA: genetic covariance analyzer; GO, Gene Ontology Biological Process; GWAS: genome-wide association study; GWAS-PW: pairwise genome-wide association study; KEGG, Kyoto Encyclopedia of Genes and Genomes; LDSC: linkage disequilibrium score regression; MTAG: multitrait analysis of genome-wide association study; SMR: summary data-based Mendelian randomization; SNP: single nucleotide polymorphism; TWAS: transcriptome-wide association studies; ρ-HESS: heritability estimator from summary statistics.

The evolution of genetic statistical methods has facilitated the understanding of the interconnected genetic basis of complex diseases, providing novel perspectives on the potential biological mechanisms behind the epidemiologic correlations. In our study, we initiated a comprehensive evaluation of the correlations and the shared genetic basis between cholelithiasis and GERD via a prospective cohort study, Mendelian randomization (MR) analyses, and a range of genetic analyses (Fig. 1).

## Methods

### Data summary

#### Prospective data from the UK Biobank

The UK Biobank (UKB) is a large-scale prospective cohort study with 502,368 participants aged 37–73 years who were recruited between 2006 and 2010 [13]. Participants visited 1 of 22 assessment centers across England, Scotland, and Wales to complete touch-screen questionnaires, verbal interviews, and physical measurements at recruitment.

Data on hospital admissions were collected regularly through linkages to the Scottish Morbidity Records, the Patient Episode Database, and Health Episode Statistics. Information on death was obtained from the National Health Service Central Register and National Health Service Digital. This study was conducted under the UK Biobank project 83339. The UK Biobank received ethical approval from the North West Multi-Centre Research Ethics Committee (21/NW/0157, 16/NW/0274, and 11/NW/0382).

Diagnostic information was sourced from primary care data, hospital admission data, and death registry records. We defined diagnoses according to the International Classification of Diseases, 10th revision (ICD-10) code K80 for cholelithiasis and K21 for GERD, respectively.

As shown in the flowchart (Supplementary Fig. S1), participants with self-reported cholelithiasis or GERD (*N* = 13,320) or without follow-up data (*N* = 1,298) were excluded, leaving 487,750 individuals. To ensure a similar distribution of follow-up time between groups, the index date of participants in the control group was manually assigned based on the distribution of the first diagnosis date of those patients with diseases of interest when conducting corresponding analyses. After excluding 69,862 participants with a history of GERD before the index date, 417,888 participants were finally included to analyze the association between cholelithiasis and GERD. After excluding 62,031 participants with a history of cholelithiasis before the index date, 425,719 participants were finally included to analyze the association between GERD and cholelithiasis.

Follow-up time was calculated from the index date to the time to diagnosis of outcome of interest or the censoring date (30 October 2022) or death, whichever occurred first.

#### Genome-wide association study datasets

Genome-wide association study (GWAS) summary data for cholelithiasis were obtained from the FinnGen databases comprising 32,894 cholelithiasis cases and 301,383 controls of European ancestry [14]. The cholelithiasis dataset was defined with the ICD-10 code K80, ICD-9 code 574, and ICD-8 code 574.

GWAS summary data for GERD were obtained from a meta-analysis of 332,601 individuals including 71,522 cases and 261,079 controls of European ancestry combining the 2 largest existing genetic studies of GERD (UKB and the QSkin study) [15]. The phenotypes ranged from self-reported GERD, ICD-10, and use of GERD medication. For the replication dataset of GERD, we utilized the summary data with 129,080 European ancestry cases and 473,524 European ancestry controls from the UK and Australian population [16]. Detailed information of sample collection, quality control, and imputation process for these datasets has been explained in the original articles [14–16]. There is no population overlap between the datasets for cholelithiasis and GERD. The GWAS summary datasets utilized in this research are publicly available, and the ethical statements can be found in the original publications corresponding to the data. Patients or the public were not involved in the design, conduct, reporting, or dissemination plans of our research.

### Statistical analysis

#### Observational analysis

To handle the missing data of the covariates, we applied multiple imputation by chained equations (MICE packages in R) [17] with a predictive mean matching method that combining regression models and nearest-neighbor matching. Five imputations and 50 iterations were performed, and 1 of the 5 imputations was selected randomly as the final imputed data set.

We constructed a Cox proportional hazards regression model with exposure to cholelithiasis to calculate the hazard ratios (HRs) and 95% confidence intervals (CIs). The proportional hazards assumption was tested by Schoenfeld residuals tests, and no evidence of violation was found. Three sets of adjustments were established to minimize the role of confounding. Model 1 was without any adjustments. Model 2 was adjusted only for age and sex. Model 3 was further adjusted for ethnicity, average total annual household income, deprivation index, body mass index (BMI), alcohol consumption, smoking status, physical activity, education, fresh fruit consumption, raw vegetable consumption, tea consumption, coffee consumption, hypertension, diabetes, renal failure, myocardial infarction, stroke, chronic obstructive pulmonary disease, asthma, anxiety, depression, and peptic ulcer. All analyses were performed using RStudio (RRID:SCR_000432) and R 4.2.1 software. Statistical significance was set at a 2-tailed *P* value of less than 0.05.

#### Mendelian randomization analysis

We performed the bidirectional MR analysis to explore the potential causal relationship between cholelithiasis and GERD, using R packages “TwoSampleMR” [18], and “MR-PRESSO” [19] in R software (version 4.2.1). MR analysis utilizes genetic variants as instruments, and the validity of its causal inference relies on 3 critical assumptions of independence, relevance, and exclusion restriction [20]. These assumptions are indispensable for mitigating bias and establishing causality. Only significant single nucleotide polymorphisms (SNPs) independently associated with the exposure at a *P* threshold of 5 × 10^–8^ and satisfying the linkage disequilibrium (LD) criteria of *r*^2^ < 0.001 and kb > 10,000 were identified as instruments in MR studies. Additionally, we searched the instrumental variables in the GWAS catalog [21] to identify potential confounders like BMI, smoking, and certain dietary habits and excluded the confounding variants from further analyses.

We employed inverse variance weighting (IVW) [22] as the main MR approach, complemented by 3 additional sensitivity analysis methods, including MR-Egger [23], weighted median [24], and weighted mode [25], to detect the causal relationships between cholelithiasis and GERD. Different methods were based on different assumptions concerning the influence of horizontal pleiotropy. The IVW MR model, assuming balanced pleiotropy, applies multiplicative random effects to meta-analyze the Wald estimates of each SNP [22]. The MR-Egger model allows the uncorrelated directional pleiotropy by adding a nonzero intercept that relaxes the assumption of relevance of selected genetic variants [23]. The weighted median and weighted mode models remain robust when up to 50% or more of genetic variants are valid, which exhibit greater resilience to pleiotropy [24, 25].

We conducted the MR-Egger intercept test, Cochran’s *Q* statistic, MR-PRESSO, and leave-one-out analysis to evaluate the heterogeneity, pleiotropy, and potential outliers of the MR results. If heterogeneity is detected in the MR analysis (*P* < 0.05), we would recalculate the MR estimates after the removal of outliers identified with a *P* value of less than 1 in the MR-PRESSO outlier test to ensure the robustness of the MR results. The MR analysis in this research has been documented in accordance with the Strengthening the Reporting of Observational Studies in Epidemiology (STROBE) guideline specific for MR study.

#### Global genetic correlation analysis

To quantify the heritability of each trait and the global genetic correlation between cholelithiasis and GERD, we applied linkage disequilibrium score regression (LDSC) method with Python 2.7 [26]. Based on precomputed LD scores derived from 1000 Genomes reference data of European population, we selected SNPs that matched the reference panel (minor allele frequency [MAF] > 0.01 and INFO score > 0.9) in the GWAS datasets [27]. We used univariate LDSC to estimate SNP heritability for each trait and bivariate LDSC to calculate the genetic correlations between cholelithiasis and GERD with and without constraining the intercept. Based on the prevalence rates of 20% [1] for cholelithiasis and 17.1% [6] for GERD, we calculated the liability scale of the reported heritability for both traits. The genetic correlation with a *P* value less than 0.05 was considered significant [28, 29].

Additionally, we employed the genetic covariance analyzer (GNOVA) as a supplementary method to validate the genetic correlations. The steps of quality control on GWAS datasets are similar to the LDSC method [30]. More detailed descriptions are in the original study [31]. Based on the framework of the annotation-stratified genetic covariance estimation, GNOVA provides a more powerful statistical inference of the shared genetic basis between complex traits and shows higher estimation accuracy. Threshold of *P* < 0.05 was regarded as strong evidence for MAF-stratified genetic correlation [31].

#### Local genetic correlation analysis

To identify whether cholelithiasis and GERD have genetic correlation in local genomic region, we further applied the heritability estimator from summary statistics (ρ-HESS) with Python 2.7 [32]. We first calculated the LD block and eigenvalues by referring to the 1000 Genomes Project of Europeans. Then, we explored the local SNP heritability for each trait and estimated the local genetic correlation in 1,613 approximately LD-independent regions [33]. Suggestive genetic associations with a *P* value less than 0.05 were noted.

Similarly, pairwise-GWAS (GWAS-PW) was supplemented to explore the significant shared local regions [34]. Based on the Bayesian statistical framework, GWAS-PW calculated the posterior probabilities of association (PPAs) for each genomic region across 4 models. Genomic regions with a PPA of model 3 larger than 0.5 were considered significantly associated with both traits, in accordance with a previous article [35, 36].

#### Cross–trait meta–analysis

To detect the shared genetic variants in cholelithiasis and GERD, we performed multitrait analysis of GWAS (MTAG) [37]. MTAG is based on a fundamental assumption that all SNPs exhibit the same variance–covariance matrix of effect sizes and heritability across traits. To meet the assumption, we rigorously filtered the MTAG SNP with MAF ≥1% and sample size ≥75% of the 90th percentile and dropped the outliers [37]. By joint analysis of multiple traits, MTAG substantially enhances the statistical power to detect the genetic associations for each trait and generate trait-specific estimates for each SNP. To identify the significant and independent loci, we utilized the threshold *P*_MTAG_ < 5 × 10^–8^ and the “clumping” function of PLINK (settings: clump_p1 = 5e^−8^, clump_p2 = 1e^−5^, clump_r^2^ = 0.2, clump_kb = 500) [38].

Cross-phenotype association test (CPASSOC) is a complementary method to deduce the shared risk SNPs between complex traits [39]. Compared with the single-trait analysis, CPASSOC improves statistical power and reasonably controlled type I error rate. Considering the heterogeneity effects for different phenotypes, we primarily used the heterogeneous version of cross-phenotype statistic (Shet) method to integrate association evidence of different but correlated traits [40]. Given the inherent variability induced by the random sampling analysis embedded in this method, we set a random seed to 123 to ensure a reproducible result. After getting the estimates, we identified the independent loci using the “clumping” function of PLINK (settings as before). The variant in each locus with the smallest *P* value was regarded as the index SNP. Index SNPs that met the criteria of *P*_CPASSOC_ < 5 × 10^–8^ and *P*_each trait_ < 1 × 10^–3^ were deemed significant pleiotropic SNPs. Newly discovered pleiotropic SNPs were defined as those significant pleiotropic SNPs that were not genome-wide significant (5 × 10^–8^ < *P*_each trait_ < 1 × 10^–3^) and independent (*r*^2^ < 0.20) of earlier identified trait-related genome-wide significant SNPs, and all their adjacent SNPs (±500 kb) did not reach *P* < 5 × 10^–8^ in each GWAS dataset.

We used dbSNP [41] and 3DSNP [42] for detailed functional annotation of the identified pleiotropic SNPs.

#### Transcriptome–wide association analysis

Numerous genetic variants impact intricate traits through the regulation of gene expression. To identify significant gene–trait associations, we implemented a transcriptome-wide association scan (TWAS) leveraging FUSION software [43]. Based on the LD reference data of European 1000 Genomes, we converted the GWASs of cholelithiasis and GERD into an LD-score format. We prioritized the trait-related tissues; thus, we prepared the expression quantitative trait loci (eQTL) data of whole blood, liver, stomach, and esophagus-related tissues from GTEx v8 (Genotype-Tissue Expression, version 8) [43]. By integrating the precomputed phenotypic summary data and corresponding eQTL data, we identified significant tissue-specific genes with a false discovery rate (FDR) <0.05 for each trait and selected genes that overlapped between cholelithiasis and GERD in the same tissue.

Summary data-based Mendelian randomization (SMR) analysis is a complementary method to deduce the causative genes underlying cholelithiasis and GERD [44]. We used the eQTL data of whole blood, liver, stomach, and esophagus-related tissues from GTEx v8 [45] and *cis*-eQTL data of whole blood from the eQTLGen consortium [46]. The heterogeneity in dependent instruments (HEIDI) test was conducted to distinguish pleiotropy or causality from linkage. We primarily focused on the genes with FDR < 0.05 and passed the *P* value thresholds for the HEIDI test (*P*_HEIDI_ > 0.05) [44].

#### The pathway enrichment analyses and biomolecular network analyses

To gain shared biological insights into cholelithiasis and GERD, we conducted functional annotation of the pleiotropic SNPs and shared genes using multiple methods. We utilized the knowledge-based Kyoto Encyclopedia of Genes and Genomes (KEGG) and Gene Ontology (GO) databases to perform pathway enrichment analyses for identifying pathways associated with these genes, using the ClusterProfiler R package (RRID:SCR_016884) [47, 48]. *P* values from the pathway enrichment analyses were adjusted for multiple comparisons through the FDR approach. In addition, we utilized the STRING database [49] to find the interactions mapped to the pleiotropic SNPs and shared functional genes.

## Results

### Observational association between cholelithiasis and GERD

Baseline characteristics of the study cohort by cholelithiasis are presented in Supplementary Table S1. In total, participants were followed for 2,736,451 person-years, during which 1,628 cholelithiasis patients and 20,780 non-cholelithiasis individuals developed GERD (Table 1A). In the age/sex-adjusted model, the risk of GERD was 2.28 times higher in cholelithiasis patients compared to those without cholelithiasis. In the fully adjusted model, the risk of GERD remained statistically significant in cholelithiasis patients (HR = 1.99; 95% CI, 1.89–2.10; *P* < 0.001).

**Table 1:** Observational association between cholelithiasis and GERD: (A) associations of cholelithiasis with the risk of GERD and (B) associations of GERD with the risk of cholelithiasis

	Case/person-years	Model 1		Model 2		Model 3	
		HR (95% CI)	*P* value	HR (95% CI)	*P* value	HR (95% CI)	*P* value
**(A) Cholelithiasis** Non-cholelithiasis	20,780/2,649,797	1.00 (reference)		1.00 (reference)		1.00 (reference)	
Cholelithiasis	1,628/86,654	2.40 (2.30–2.52)	<0.001	2.28 (2.17–2.40)	<0.001	1.99 (1.89–2.10)	<0.001
**(B) GERD**							
Non-GERD	5,883/2,372,960	1.00 (reference)		1.00 (reference)		1.00 (reference)	
GERD	1,066/220,497	2.86 (2.70–3.02)	<0.001	2.69 (2.54–2.84)	<0.001	2.30 (2.18–2.44)	<0.001

Model 1: without any adjustments.

Model 2: adjusted for age and sex.

Model 3: adjusted for age, sex, ethnicity, average total annual household income, deprivation index, body mass index, alcohol consumption, smoking status, physical activity, education, fresh fruit consumption, raw vegetable consumption, tea consumption, coffee consumption, hypertension, diabetes, renal failure, myocardial infarction, stroke, chronic obstructive pulmonary disease, asthma, anxiety, depression, and peptic ulcer.

Moreover, we also observed the association between baseline GERD and incident cholelithiasis, as shown in Table 1B. In the age/sex-adjusted model, the HR for cholelithiasis was 2.69 (95% CI, 2.54–2.84; *P* < 0.001) for GERD patients. In the fully adjusted model, the GERD group also displayed a significantly increased risk of developing cholelithiasis (HR = 2.30; 95% CI, 2.18–2.44, *P* < 0.001).

### Causal association between cholelithiasis and GERD

**Figure 2:**
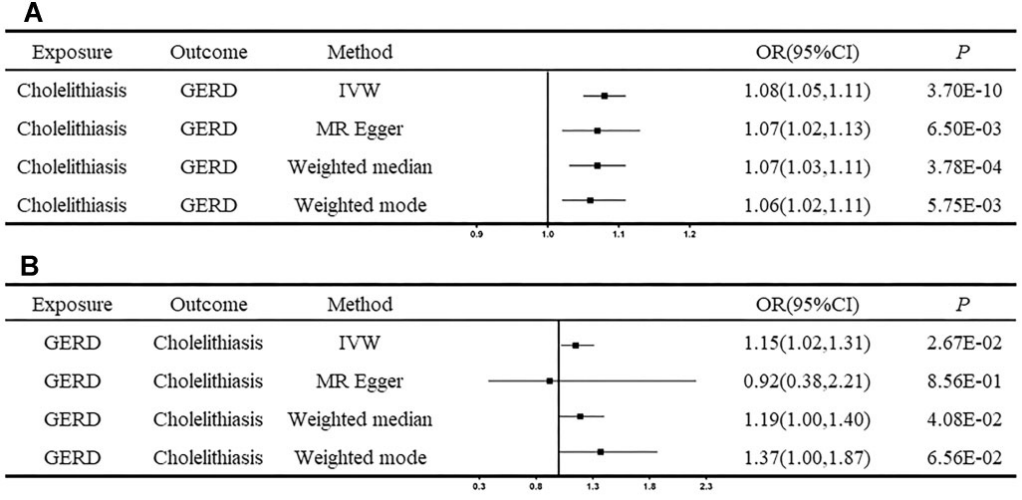
The causal associations between cholelithiasis and GERD. (A) The causal effect of cholelithiasis on GERD. (B) The causal effect of GERD on cholelithiasis. Error bars represent the 95% CIs for the estimates. CI: confidence interval; GERD: gastroesophageal reflux disease; IVW: inverse variance weighted.

After excluding the confounding instrumental variables, we used 46 cholelithiasis-associated and 20 GERD-associated genetic instruments (Supplementary Table S2), respectively, in the analyses and provided evidence for the causal association between cholelithiasis and GERD. Genetically determined cholelithiasis has the possibility to increase the risk of GERD by 8% (IVW OR = 1.08; 95% CI, 1.05–1.11; *P* = 3.70 × 10^–10^; Fig. 2A, Supplementary Table S3), which was further validated by the other 3 MR methods and the analyses with a supplementary dataset (Supplementary Table S4). Besides, we also conducted a reverse MR analysis and found that genetically predicted GERD could increase the risk of cholelithiasis by 15% (OR = 1.15; 95% CI, 1.02–1.31; *P* = 0.027) according to IVW method (Fig. 2B, Supplementary Table S3). This association was further validated through analyses employing the weighted median method and an additional dataset (Supplementary Tables S3–S4), although it could not be confirmed using the MR-Egger and the weighted mode methods.

The *F* statistic of each SNP related to cholelithiasis and GERD was larger than the empirical threshold of 10, suggesting little possibility of weak instrument bias (Supplementary Table S2). We also performed several sensitive analyses to validate the causal association between cholelithiasis and GERD. Cochran’s *Q* test in the IVW model and the MR-Egger model suggested a lack of evidence for the existence of heterogeneity in effects across the instrumental variables. The *P* value of the MR-Egger intercept test was larger than 0.05, which indicated that there was a lower possibility of horizontal pleiotropy in the causal estimates (Supplementary Tables S3–S4). The leave-one-out analysis suggested that the observed causal relationship was not influenced by any outliers (Supplementary Figs. S2–S3). The scatterplots, forest plots, and funnel plots of the MR results are displayed in Supplementary Figs. S2–S3.

### Global and local genetic correlations between cholelithiasis and GERD

SNP-based liability-scale heritability *h*² for cholelithiasis and GERD was 26.65% and 14.01% when utilizing the univariate LDSC with constraining the intercept. The observed heritability of cholelithiasis and GERD was 6.60% and 7.68% utilizing GNOVA. The cross-trait LDSC suggested that cholelithiasis had a relatively strong positive genetic correlation with GERD, exhibiting a genetic correlation (r_g_) of 0.31 and a *P* value of 2.77 × 10^–27^. After constraining the intercept, the genetic correlation was decreased but remained significant (r_g_ = 0.25, *P* = 3.90 × 10^–56^). This finding was consistent with the GNOVA analysis, reflecting a genetic correlation (r_g_) of 0.26 and a *P* value of 2.50 × 10^–32^ (Table 2).

**Table 2:** Heritability and genetic correlation between cholelithiasis and GERD

		Cholelithiasis	GERD
Total liability scale heritability	LDSC without constraining the intercept	0.1695	0.1251
Total liability scale heritability	LDSC with constraining the intercept	0.2665	0.1401
Observed heritability	GNOVA	0.0659	0.0768
Genetic correlation (r_g_), *P*	LDSC without constraining the intercept	0.3053, 2.77 × 10^–27^	
Genetic correlation (r_g_), *P*	LDSC with constraining the intercept	0.2499, 3.90 × 10^–56^	
Genetic correlation (r_g_), *P*	GNOVA	0.2625, 2.50 × 10^–32^	

We also tested the local genetic correlation by ρ-HESS and GWAS-PW (Supplementary Table S5). Seven suggestively significant regions were identified by ρ-HESS, and 8 significant regions were identified by GWAS-PW. Four regions were overlapped according to ρ-HESS and GWAS-PW. These findings suggested a potential shared genetic foundation, necessitating further exploration to elucidate the underlying biological mechanisms.

### Identification of shared risk loci for cholelithiasis and GERD

MTAG identified 8 independent pleiotropic loci (rs146812426, rs4299376, rs6733452, rs7596134, rs4681515, rs9297994, rs10935762, rs3922717), which were also significant in CPASSOC (Table 3, Supplementary Table S6). CPASSOC found 23 pleiotropic loci, 5 of which were significant in MTAG, including rs9297994, rs10935762, rs3922717, rs12633863, and rs802036 (Table 3, Supplementary Table S7). Overall, 10 independently significant loci have been identified as shared between cholelithiasis and GERD by both MTAG and CPASSOC, namely, rs146812426, rs4299376, rs6733452, rs7596134, rs10935762, rs12633863, rs4681515, rs3922717, rs802036, and rs9297994, which mapped to 9 genes, including *PLEKHH2, ABCG8, DYNC2LI1, ABCG5, TM4SF4, LOC100270746, CROT, UBXN2B*, and *CYP7A1* (Table 3). It is worth noting that 5 novel pleiotropic loci were identified in the CPASSOC analysis, including rs10167227, rs6742945, rs335208, rs72664027, and rs11537754, which mapped to genes *PNPT1, LOC105369165, PRDM6, LINC02842*, and *RAB11FIP3*, respectively (Table 3, Supplementary Table S7). Other SNP-associated genes are listed in Supplementary Tables S6–S7.

**Table 3:** Genome-wide significant loci shared between cholelithiasis and GERD in cross-trait meta-analyses

SNP	Chromosome	Base pairs	A1	A2	Odds ratio		Cross-trait meta-analyses		*P*_MTAG		*P*_CPASSOC	Gene
					Cholelithiasis	GERD	MTAG	CPASSOC	Cholelithiasis	GERD		
**rs10167227**	2	56,004,781	T	C	1.055259069	1.041435448	**−**	**+**	9.28E-06	7.90E-07	**2.93E-08**	*PNPT1* *
**rs6742945**	2	53,201,324	T	C	1.033076972	1.02716235	**−**	**+**	3.38E-06	1.10E-06	**1.43E-08**	*LOC105369165*
**rs335208**	5	122,503,245	G	A	1.033058996	0.973653294	**−**	**+**	2.58E-06	5.66E-07	**6.47E-09**	*PRDM6*
**rs72664027**	8	62,948,007	G	A	1.157742514	0.927187007	**−**	**+**	5.03E-06	3.49E-06	**3.89E-08**	*LINC02842*
**rs11537754**	16	570,557	C	T	1.037051717	0.975700114	**−**	**+**	8.29E-07	2.31E-06	**5.95E-09**	*RAB11FIP3*
rs146812426 ^a^	2	43,909,666	A	G	1.733518226	1.057174729	**+**	**+**	1.26E-93	2.48E-08	3.68E-121	*PLEKHH2*
rs4299376 ^a^	2	44,072,576	T	G	1.3178505	1.017247042	**+**	**+**	5.88E-124	2.12E-12	2.10E-158	*ABCG8*
rs6733452 ^a^	2	44,094,845	A	G	1.775888747	1.085672923	**+**	**+**	1.63E-120	9.66E-14	1.04E-151	*ABCG8*
rs7596134 ^a^	2	44,052,833	A	C	1.324747665	1.018366629	**+**	**+**	8.81E-175	3.78E-15	8.31E-227	*DYNC2LI1, ABCG5*
rs4681515 ^a^	3	149,212,076	G	A	0.879264075	1.024597651	**+**	**+**	1.35E-48	5.58E-11	3.67E-55	*TM4SF4*
rs9297994 ^ab^	8	59,392,324	A	G	0.888037773	0.974529976	**+**	**+**	2.25E-40	2.40E-10	1.06E-44	*UBXN2B, CYP7A1*
rs10935762 ^ab^	3	149,216,298	T	C	0.850727852	0.960117122	**+**	**+**	5.12E-42	1.33E-09	1.55E-47	*TM4SF4*
rs3922717 ^ab^	6	27,030,924	G	A	0.959186164	1.043833506	**+**	**+**	2.16E-08	1.99E-11	1.07E-13	*LOC100270746* *
rs12633863 ^b^	3	149,211,512	A	G	0.878788522	0.977457956	**+**	**+**	2.03E-48	2.63E-10	1.56E-55	*TM4SF4*
rs802036 ^b^	7	86,977,894	C	T	0.865221271	1.050850672	**+**	**+**	5.58E-21	5.84E-09	1.52E-22	*CROT*

* Genes that interact the SNP through 3-dimensional chromatin loops in different cell types.

a Independent pleiotropic loci in MTAG and significant in CPASSOC.

b Independent pleiotropic loci in CPASSOC and significant in MTAG.

The bolded SNPs represent the independent new loci shared between cholelithiasis and GERD identified in the CPASSOC method.

**Figure 3:**
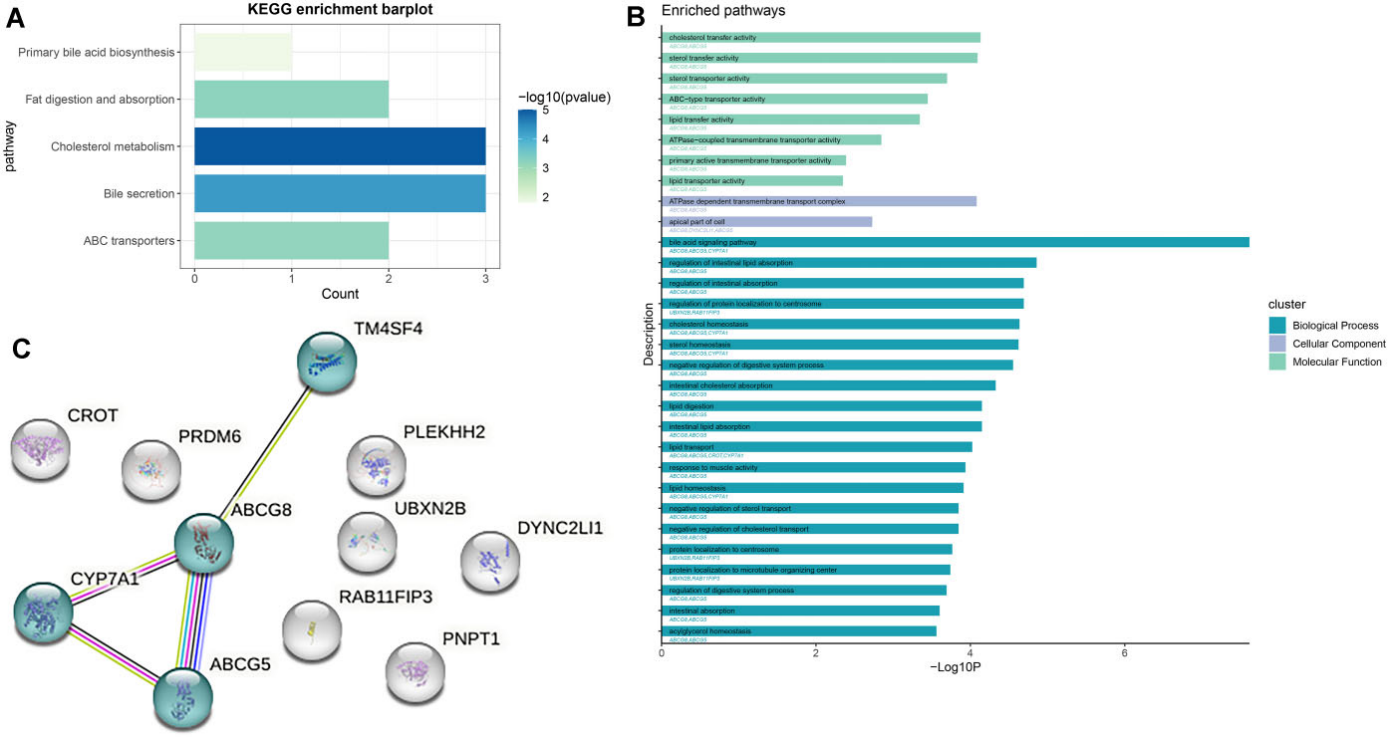
Enriched pathways identified using KEGG and GO databases and network of genes associated with pleiotropic variants. (A) Enriched pathways identified using the KEGG database. (B) Enriched pathways identified using the GO database. (C) The network of genes associated with pleiotropic variants.

After multiple corrections, the pathway enrichment analysis using the KEGG database identified 5 pathways according to the above genes, including cholesterol metabolism, bile secretion, fat digestion and absorption, ABC transporters, and primary bile acid biosynthesis (Fig. 3A, Supplementary Table S8). The pathway enrichment analysis using the GO database identified 65 biological processes, 2 cellular components, and 8 molecular functions; most of these pathways are related to lipid and bile acid metabolism (Fig. 3B, Supplementary Table S9). In the network analysis, we observed a close association among *TM4SF4, CYP7A1, ABCG5*, and *ABCG8* (Fig. 3C).

### Identification of shared genes for cholelithiasis and GERD

Results from tissue-specific TWAS and SMR revealed gene-level genetic overlap. After FDR corrections, a total of 15 genes were shared by cholelithiasis and GERD and enriched in 6 tissues, including blood, liver, esophagus mucosa, esophagus muscularis, esophagus gastroesophageal junction, and stomach in the TWAS analysis (Supplementary Table S10). Among them, 7 genes significantly overlapped in 2 or more tissues. Five of 7 genes (*SUN2, CBY1, JOSD1, DDX17, FAM227A*) were located in 22q13.1. The TWAS analysis showed that overexpression of *SUN2, JOSD1*, and *CBY1* was negatively associated with the risk of cholelithiasis and GERD in the blood and esophagus-related tissues, while overexpression of *JOSD1* and *CBY1* was positively associated with these 2 diseases in the liver tissue. *SUN2, JOSD1*, and *CBY1* also displayed a significant SMR association signal with *FDR* < 0.05 and passed the HEIDI-outlier test in blood, esophagus mucosa, and esophagus muscularis (Supplementary Table S11). No significant shared causal gene was found in other tissues, namely liver, esophagus gastroesophageal junction, and stomach, according to SMR results.

**Figure 4:**
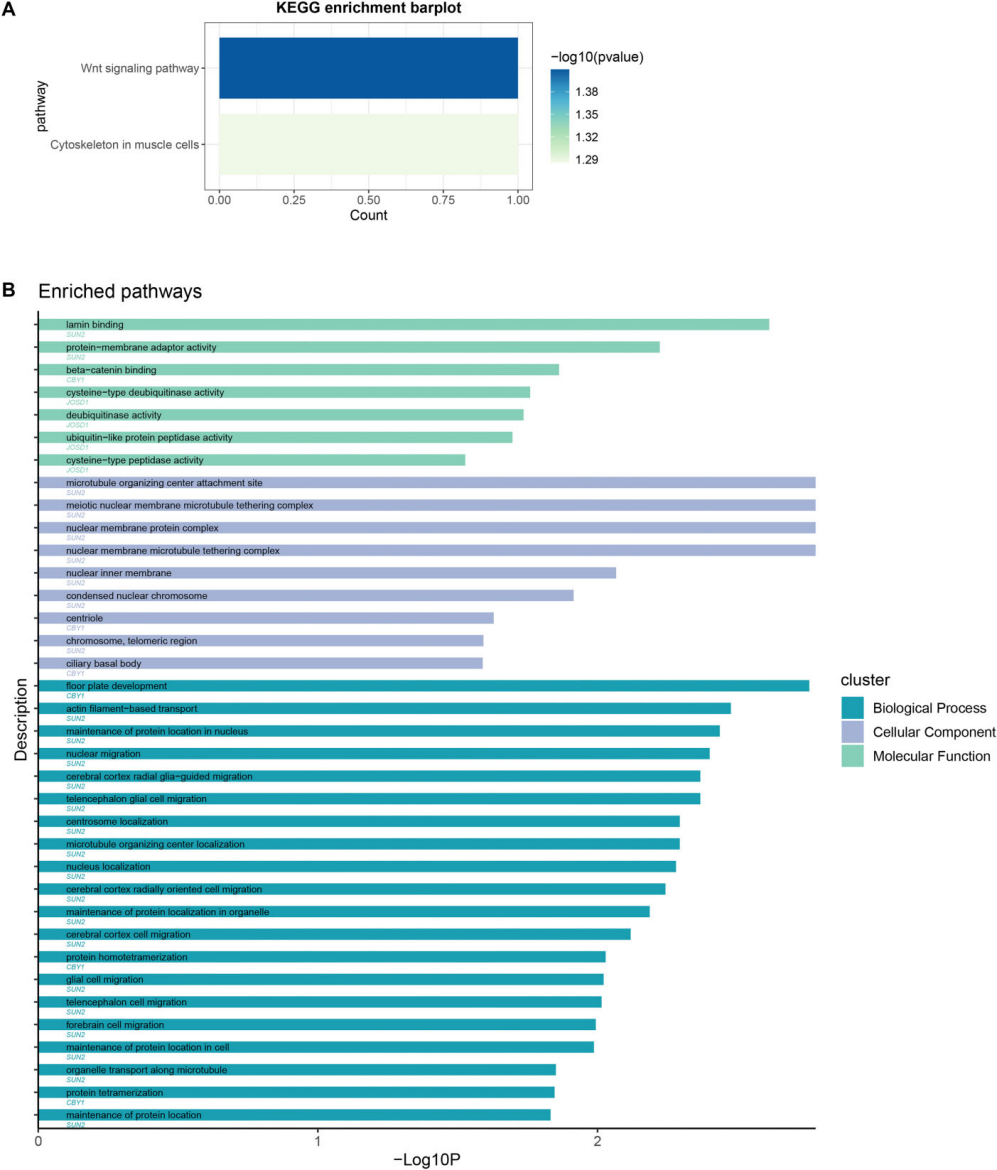
Enriched pathways of shared genes identified using KEGG and GO databases. (A) Enriched pathways identified using the KEGG database. (B) Enriched pathways identified using the GO database.

Using the KEGG database, we found 2 significantly enriched pathways, Wnt signaling pathway (*CBY1*) and cytoskeleton in muscle cells (*SUN2*) (Fig. 4A, Supplementary Table S12). Wnt signaling pathway (*CBY1*) also enriched significantly in the GO pathway enrichment analysis, which is shown in Fig. 4B and Supplementary Table S13. In the network analysis, we did not identify the association between these 3 shared genes.

## Discussion

To our knowledge, this is the first study to comprehensively explore the observational, causal, and genetic relationships between cholelithiasis and GERD. By leveraging UK Biobank data and GWAS data, we found the bidirectional causal relationship between cholelithiasis and GERD. The subsequent genetic analyses provided new insights into their shared genetic basis and related biological mechanism, which may contribute to the prediction, diagnosis, and treatment of these diseases.

Previous research has reported that cholelithiasis and GERD shared numerous common etiological risk factors such as obesity [50], type 2 diabetes mellitus [51], depression [52], and smoking [53]. We conducted a Cox proportional hazards regression model analysis using the UKB cohort, with adjustments for a wide range of established and potential confounders associated with these 2 conditions. Although the HRs were slightly attenuated after controlling the covariates, the bidirectional association between cholelithiasis and GERD remained statistically significant. This is consistent with the findings by Unalp-Arida et al. [11] and Portincasa et al. [8], which reported a statistically significant association between cholelithiasis and GERD. Subsequently, using the MR approach, we identified bidirectional causality between cholelithiasis and GERD, while the pathophysiologic mechanisms underlying the causal relationship remain unclear. Previous studies suggested that patients with gallstones showed impaired gastric motility [4, 8], which might be related to the pathogenesis of GERD. Meanwhile, patients with GERD presented a higher incidence of gallbladder dyskinesia [54, 55], which may be attributed to the routine use of proton pump inhibitors (PPIs) in GERD treatment. It has been reported that PPIs could reduce the release of cholecystokinin, which might diminish gallbladder motility, thereby causing the formation of gallstones [56]. The current evidence indicated the potential shared pathogenesis or genetic basis between cholelithiasis and GERD, warranting further exploration.

In the analysis of heritability and genetic correlation, the heritability of cholelithiasis and GERD was estimated to be 17% and 13%, respectively, indicating a significant genetic contribution to the etiology of both diseases, consistent with previous studies [15, 57]. The genetic correlation between cholelithiasis and GERD was 0.31, suggesting a moderate to strong genetic association between these conditions. The finding supports the hypothesis that genetic factors, such as local genetic correlations, shared loci, and common functional genes, play an important role in the co-occurrence of cholelithiasis and GERD. We identified 4 regions that exhibited a suggestively significant local genetic association, as evidenced by ρ-HESS < 0.05 and GWAS-PW > 0.5. Most loci identified by MTAG and CPASSOC were situated within these regions. Moreover, we found that 22q13.1 might be a shared region between gallstone disease and GERD by combining analyses of local genetic correlation, shared loci, and shared genes: First, this region showed suggestively significant local genetic association between cholelithiasis and GERD using GWAS-PW. Second, the shared loci rs1056661, identified by CPASSOC, were located within this region. Third, 5 and 3 overlapped genes, identified from TWAS analysis and SMR, respectively, were situated within this region. Previous studies have reported that several significant loci related to gallstone disease, including rs12004, rs41281265, and rs1946990, were in this region [58, 59]. However, currently, there is no research linking this region to GERD. Future research is warranted to delve deeper into this specific region to elucidate the genetic correlation between gallstone disease and GERD.

Given the significant genetic correlation observed, we conducted cross-trait GWAS meta-analyses to detect risk SNPs underlying the joint phenotypes of cholelithiasis–GERD. We identified 10 shared independently significant loci through MTAG and CPASSOC. According to the results of pathway enrichment analyses, the genes associated with these loci were enriched in pathways related to lipid and bile acid metabolism, including cholesterol metabolism, bile secretion, ABC transporters, and primary bile acid biosynthesis. Several studies have reported that aberrant lipid and bile acid metabolism contributes to the development of both cholelithiasis and GERD [1, 60–62]. *ABCG5* (index SNP: rs7596134), *ABCG8* (index SNP: rs4299376 and rs6733452), and *CYP7A1* (index SNP: rs9297994) are associated with lipid metabolism. Numerous investigations have suggested the involvement of these genes in the development of gallstone disease [63–65]. Although several studies have reported that obesity [50] and dyslipidemia [60] are risk factors for GERD, no research has investigated the involvement of these genes in GERD. Therefore, the relationship between these genes and GERD warrants further investigation. Additionally, 5 new loci associated with cholelithiasis and GERD were identified via CPASSOC analysis. *PNPT1* (index SNP: rs10167227) is associated with the mitochondrial respiratory chain, and mutations in *PNPT1* can lead to mitochondrial dysfunction, subsequently causing neuromuscular dysfunction, which affects the peristaltic function of the gastrointestinal tract [66, 67]. The functions of long noncoding RNA (lncRNA) gene *LINC02842* (index SNP: rs72664027) and noncoding RNA (ncRNA) gene *LOC105369165* (index SNP: rs6742945) remain unclear, but research has suggested that lncRNAs might have a crucial role in the dysfunction of the lower esophageal sphincter (LES) [68], potentially shedding light on the onset of GERD. Additional research is required to offer more detailed functional annotation of these shared loci.

In addition to detecting shared loci, we also explored whether the cholelithiasis–GERD association can be mediated by shared risk genes through TWAS and SMR analysis. In general, we identified 3 putatively functional genes shared between cholelithiasis and GERD, including *SUN2, CBY1*, and *JOSD1*, overexpression of which was negatively associated with the risk of cholelithiasis and GERD in the esophagus-related tissues. Prior research has reported the negative effect of *CBY1* and *SUN2* genes on tumorigenesis [69–71], which implied a potential role of them in the pathogenesis of gallstone disease and GERD, given that these 2 diseases are risk factors for gallbladder and esophageal cancer, respectively [72, 73]. Furthermore, existing studies suggested the involvement of bile acids in GERD progression through the activation of the Wnt/β-catenin pathway [74]. *CBY1* might be involved in the linkage between gallstone disease and GERD, as it can inhibit the Wnt/β-catenin pathway [75], which was enriched according to the results of the pathway enrichment analyses. *JOSD1* is a deubiquitinating enzyme, playing a pivotal role in many cellular biological processes [76]. Our findings imply that *JOSD1* may play a significant role in the associative mechanisms between cholelithiasis and GERD via the deubiquitination processes. In general, our study offers novel insights into the underlying shared genetic basis of cholelithiasis and GERD, and additional research is required for a more profound elucidation.

## Strengths and Limitations

In our study, we conducted the largest prospective study assessing the phenotypic association between cholelithiasis and incident GERD. Besides, we performed a series of sensitive analyses and further applied validation datasets in MR estimates to enhance the robustness of our results. Furthermore, genetic correlation, pleiotropic loci, and gene detection were fully analyzed by 2 different approaches. The convergent evidence acquired through these dual approaches reinforces the reliability of our findings. However, several limitations need to be acknowledged. First, the causal relationship from GERD to cholelithiasis was not significant in all sensitivity analyses, which may be attributed to the limitations of GWAS statistics. Therefore, larger and more powerful GWAS data for cholelithiasis and GERD will be needed to establish the causal relationships from GERD to cholelithiasis. Second, all the data used in this study came from European ancestry populations, which limited the extension of our findings to other ethnic populations; thus, future studies involving a broader range of ancestries are warranted. Third, due to limited GWAS data availability at the time of conducting the analysis, we were unable to perform a deeper subgroup analysis based on the stratification information, such as age, gender, and severity of the disease.

## Conclusion

In summary, we found a bidirectional association between cholelithiasis and GERD, which may be attributed to a bidirectional causal relationship and a shared genetic basis, including the significant genetic correlation, novel shared loci, and genes. Our findings provided new insights into the biological mechanisms for cholelithiasis and GERD and suggested promising therapeutic targets, which might provide an innovative research direction for future therapeutic strategy and risk prediction.

## Supplementary Material

giaf023_Supplemental_Files

giaf023_GIGA-D-24-00123_Original_Submission

giaf023_GIGA-D-24-00123_Revision_1

giaf023_Response_to_Reviewer_Comments_Original_Submission

giaf023_Reviewer_1_Report_Original_Submission Jian Zeng -- 7/9/2024

giaf023_Reviewer_1_Report_Revision_1 Jian Zeng -- 12/15/2024

giaf023_Reviewer_2_Report_Original_Submission Mangala Hegde -- 8/12/2024

## Data Availability

Primary data from the UK Biobank resource are accessible upon application. Dataset of cholelithiasis was downloaded from the FinnGen study [14] and datasets of gastroesophageal reflux disease were downloaded at Figshare [15, 77] and GWAS catalog (GCST90000514). All supporting data and materials are available in the *GigaScience* database, GigaDB [78].

## Additional Files

**Supplementary Table S1**. Baseline characteristics of UK Biobank participants.

**Supplementary Table S2**. Instrumental variables employed in the primary GWAS data for bidirectional Mendelian randomization analysis.

**Supplementary Table S3**. Summarized results of bidirectional Mendelian randomization analysis and sensitive analysis about cholelithiasis and gastroesophageal reflux disease in the primary datasets.

**Supplementary Table S4**. Summarized results of bidirectional Mendelian randomization analysis and sensitive analysis about cholelithiasis and gastroesophageal reflux disease in the replication datasets.

**Supplementary Table S5**. Characteristics of the regions of the genome with a genetic correlation between cholelithiasis and gastroesophageal reflux disease detected by ρ_HESS and GWAS-PW.

**Supplementary Table S6**. Independent genome-wide significant loci shared between cholelithiasis and gastroesophageal reflux disease in MTAG.

**Supplementary Table S7**. Independent genome-wide significant loci shared between cholelithiasis and gastroesophageal reflux disease in CPASSOC.

**Supplementary Table S8**. Enriched pathways of genes associated with pleiotropic variants based on the KEGG database.

**Supplementary Table S9**. Significantly enriched pathways of genes associated with pleiotropic variants based on the GO database.

**Supplementary Table S10**. TWAS-prioritized genes associated with cholelithiasis and gastroesophageal reflux disease.

**Supplementary Table S11**. SMR-prioritized genes associated with cholelithiasis and gastroesophageal reflux disease.

**Supplementary Table S12**. Enriched pathways of shared genes based on the KEGG database.

**Supplementary Table S13**. Significantly enriched pathways of shared genes based on the GO database.

**Supplementary Fig. S1**. The flowchart of the prospective cohort study.

**Supplementary Fig. S2**. Forest plot (A), leave-one-out analysis (B), scatterplot (C), and funnel plot (D) of the causal effect of cholelithiasis on gastroesophageal reflux disease.

**Supplementary Fig. S3**. Forest plot (A), leave-one-out analysis (B), scatterplot (C), and funnel plot (D) of the causal effect of gastroesophageal reflux disease on cholelithiasis.

## Abbreviations

bp: base pair; CHR: chromosome; CI: confidence interval; CPASSOC: cross-phenotype association test; FDR: false discovery rate; GERD: gastroesophageal reflux disease; GNOVA: genetic covariance analyzer; GWAS: genome-wide association study; GWAS-PW: pairwise genome-wide association study; HR: hazard ratio; LDSC: linkage disequilibrium score regression; MR: Mendelian randomization; MTAG: multitrait analysis of genome-wide association study; SMR: summary data-based Mendelian randomization; SNP: single nucleotide polymorphism; TWAS: transcriptome-wide association studies; ρ-HESS: heritability estimator from summary statistics.

## References

[1] Lammert F, Gurusamy K, Ko CW, et al. Gallstones. Nat Rev Dis Primers. 2016;2:16024. 10.1038/nrdp.2016.24. 27121416

[2] Wang F, Wang J, Li Y, et al. Gallstone disease and type 2 diabetes risk: a Mendelian randomization study. Hepatology. 2019;70(2):610–20. 10.1002/hep.30403. 30515881

[3] Katsika D, Grjibovski A, Einarsson C, et al. Genetic and environmental influences on symptomatic gallstone disease: a Swedish study of 43,141 twin pairs. Hepatology. 2005;41(5):1138–43. 10.1002/hep.20654. 15747383

[4] Di Ciaula A, Molina-Molina E, Bonfrate L, et al. Gastrointestinal defects in gallstone and cholecystectomized patients. Eur J Clin Invest. 2019;49(3):e13066. 10.1111/eci.13066. 30592298 PMC8118136

[5] Richter JE, Rubenstein JH. Presentation and epidemiology of gastroesophageal reflux disease. Gastroenterology. 2018;154(2):267–76. 10.1053/j.gastro.2017.07.045. 28780072 PMC5797499

[6] Maret-Ouda J, Markar SR, Lagergren J. Gastroesophageal reflux disease. JAMA. 2020;324(24):2565. 10.1001/jama.2020.21573. 33351044

[7] Katzka DA, Kahrilas PJ. Advances in the diagnosis and management of gastroesophageal reflux disease. BMJ. 2020;371:m3786. 10.1136/bmj.m3786. 33229333

[8] Portincasa P, Di Ciaula A, Palmieri V, et al. Impaired gallbladder and gastric motility and pathological gastro-oesophageal reflux in gallstone patients. Eur J Clin Invest. 1997;27(8):653–61. 10.1046/j.1365-2362.1997.1600709.x. 9279528

[9] Avidan B, Sonnenberg A, Schnell TG, et al. No association between gallstones and gastroesophageal reflux disease. Am J Gastroenterol. 2001;96(10):2858–62. 10.1111/j.1572-0241.2001.04238.x. 11693317

[10] Räihä I, Impivaara O, Seppälä M, et al. Determinants of symptoms suggestive of gastroesophageal reflux disease in the elderly. Scand J Gastroenterol. 1993;28(11):1011–14. 10.3109/00365529309098301. 8284623

[11] Unalp-Arida A, Der JS, Ruhl CE. Longitudinal study of comorbidities and clinical outcomes in persons with gallstone disease using electronic health records. J Gastrointest Surg. 2023;27:2843–56. 10.1007/s11605-023-05861-z. 37914859

[12] Zhu Z, Hasegawa K, Camargo CA, et al. Investigating asthma heterogeneity through shared and distinct genetics: insights from genome-wide cross-trait analysis. J Allergy Clin Immunol. 2021;147(3):796–807. 10.1016/j.jaci.2020.07.004. 32693092 PMC7368660

[13] Sudlow C, Gallacher J, Allen N, et al. UK Biobank: an open access resource for identifying the causes of a wide range of complex diseases of middle and old age. PLoS Med. 2015;12(3):e1001779. 10.1371/journal.pmed.1001779. 25826379 PMC4380465

[14] Kurki MI, Karjalainen J, Palta P, et al. FinnGen provides genetic insights from a well-phenotyped isolated population. Nature. 2023;613(7944):508–18. 10.1038/s41586-022-05473-8. 36653562 PMC9849126

[15] An J, Gharahkhani P, Law MH, et al. Gastroesophageal reflux GWAS identifies risk loci that also associate with subsequent severe esophageal diseases. Nat Commun. 2019;10(1):4219. 10.1038/s41467-019-11968-2. 31527586 PMC6746768

[16] Ong JS, An J, Han X, et al. Multitrait genetic association analysis identifies 50 new risk loci for gastro-oesophageal reflux, seven new loci for Barrett's oesophagus and provides insights into clinical heterogeneity in reflux diagnosis. Gut. 2022;71(6):1053–61. 10.1136/gutjnl-2020-323906. 34187846 PMC9120377

[17] Jolani S, Debray TPA, Koffijberg H, et al. Imputation of systematically missing predictors in an individual participant data meta-analysis: a generalized approach using MICE. Stat Med. 2015;34(11):1841–63. 10.1002/sim.6451. 25663182

[18] Hemani G, Zheng J, Elsworth B, et al. The MR-Base platform supports systematic causal inference across the human phenome. eLife. 2018;7;e34408. 10.7554/eLife.34408. 29846171 PMC5976434

[19] Verbanck M, Chen C-Y, Neale B, et al. Detection of widespread horizontal pleiotropy in causal relationships inferred from Mendelian randomization between complex traits and diseases. Nat Genet. 2018;50(5):693–98. 10.1038/s41588-018-0099-7. 29686387 PMC6083837

[20] Davies NM, Holmes MV, Davey Smith G. Reading Mendelian randomisation studies: a guide, glossary, and checklist for clinicians. BMJ. 2018;362:k601. 10.1136/bmj.k601. 30002074 PMC6041728

[21] Cerezo M, Sollis E, Ji Y, et al. The NHGRI-EBI GWAS catalog: standards for reusability, sustainability and diversity. Nucleic Acids Res. 2025; 53(D1):D998–D1005. 10.1093/nar/gkae1070. 39530240 PMC11701593

[22] Burgess S, Butterworth A, Thompson SG. Mendelian randomization analysis with multiple genetic variants using summarized data. Genet Epidemiol. 2013;37(7):658–65. 10.1002/gepi.21758. 24114802 PMC4377079

[23] Burgess S, Thompson SG. Interpreting findings from Mendelian randomization using the MR-Egger method. Eur J Epidemiol. 2017;32(5):377–89. 10.1007/s10654-017-0255-x. 28527048 PMC5506233

[24] Bowden J, Davey Smith G, Haycock PC, et al. Consistent estimation in Mendelian randomization with some invalid instruments using a weighted median estimator. Genet Epidemiol. 2016;40(4):304–14. 10.1002/gepi.21965. 27061298 PMC4849733

[25] Hartwig FP, Davey Smith G, Bowden J. Robust inference in summary data Mendelian randomization via the zero modal pleiotropy assumption. Int J Epidemiol. 2017;46(6):1985–98. 10.1093/ije/dyx102. 29040600 PMC5837715

[26] Finucane HK, Bulik-Sullivan B, Gusev A, et al. Partitioning heritability by functional annotation using genome-wide association summary statistics. Nat Genet. 2015;47(11):1228–35. 10.1038/ng.3404. 26414678 PMC4626285

[27] 1000 Genomes Project Consortium, Auton A, Brooks LD, et al. A global reference for human genetic variation. Nature. 2015;526(7571):68–74. 10.1038/nature15393. 26432245 PMC4750478

[28] Bulik-Sullivan B, Finucane HK, Anttila V, et al. An atlas of genetic correlations across human diseases and traits. Nat Genet. 2015;47(11):1236–41. 10.1038/ng.3406. 26414676 PMC4797329

[29] Yao Y, Li C, Meng P, et al. An atlas of genetic correlations between gestational age and common psychiatric disorders. Autism Res. 2022;15(6):1008–17. 10.1002/aur.2719. 35384380

[30] Perry BI, Bowker N, Burgess S, et al. Evidence for shared genetic aetiology between schizophrenia, cardiometabolic, and inflammation-related traits: genetic correlation and colocalization analyses. Schizophr Bull Open. 2022;3(1):sgac001. 10.1093/schizbullopen/sgac001. 35156041 PMC8827407

[31] Lu Q, Li B, Ou D, et al. A powerful approach to estimating annotation-stratified genetic covariance via GWAS summary statistics. Am Hum Genet. 2017;101(6):939–64. 10.1016/j.ajhg.2017.11.001. PMC5812911 29220677

[32] Shi H, Mancuso N, Spendlove S, et al. Local genetic correlation gives insights into the shared genetic architecture of complex traits. Am Hum Genet. 2017;101(5):737–51. 10.1016/j.ajhg.2017.09.022. PMC5673668 29100087

[33] Berisa T, Pickrell JK. Approximately independent linkage disequilibrium blocks in human populations. Bioinformatics. 2016;32(2):283–85. 10.1093/bioinformatics/btv546. 26395773 PMC4731402

[34] Pickrell JK, Berisa T, Liu JZ, et al. Detection and interpretation of shared genetic influences on 42 human traits. Nat Genet. 2016;48(7):709–17. 10.1038/ng.3570. 27182965 PMC5207801

[35] Mortlock S, Corona RI, Kho PF, et al. A multi-level investigation of the genetic relationship between endome triosis and ovarian cancer histotypes. Cell Rep Med. 2022;3(3):100542. 10.1016/j.xcrm.2022.100542. 35492879 PMC9040176

[36] Wu X, Zhang W, Zhao X, et al. Investigating the relationship between depression and breast cancer: observational and genetic analyses. BMC Med. 2023;21(1):170. 10.1186/s12916-023-02876-w. 37143087 PMC10161423

[37] Turley P, Walters RK, Maghzian O, et al. Multi-trait analysis of genome-wide association summary statistics using MTAG. Nat Genet. 2018;50(2):229–37. 10.1038/s41588-017-0009-4. 29292387 PMC5805593

[38] Chang CC, Chow CC, Tellier LCAM, et al. Second-generation PLINK: rising to the challenge of larger and richer datasets. Gigascience. 2015;4(1):s13742–015-0047-8. 10.1186/s13742-015-0047-8. PMC4342193 25722852

[39] Zhu X, Feng T, Tayo BO, et al. Meta-analysis of correlated traits via summary statistics from GWASs with an application in hypertension. Am Hum Genet. 2015;96(1):21–36. 10.1016/j.ajhg.2014.11.011. PMC4289691 25500260

[40] Li X, Zhu X. Cross-phenotype association analysis using summary statistics from GWAS. Methods Mol Biol. 2017;1666:455–67. 10.1007/978-1-4939-7274-6_22. 28980259 PMC6417431

[41] Phan L, Zhang H, Wang Q, et al. The evolution of dbSNP: 25 years of impact in genomic research. Nucleic Acids Res. 2025;53(D1):D925–31. 10.1093/nar/gkae977. 39530225 PMC11701571

[42] Quan C, Ping J, Lu H, et al. 3DSNP 2.0: update and expansion of the noncoding genomic variant annotation database. Nucleic Acids Res. 2022;50(D1):D950–55. 10.1093/nar/gkab1008. 34723317 PMC8728236

[43] Gusev A, Ko A, Shi H, et al. Integrative approaches for large-scale transcriptome-wide association studies. Nat Genet. 2016;48(3):245–52. 10.1038/ng.3506. 26854917 PMC4767558

[44] Zhu Z, Zhang F, Hu H, et al. Integration of summary data from GWAS and eQTL studies predicts complex trait gene targets. Nat Genet. 2016;48(5):481–87. 10.1038/ng.3538. 27019110

[45] Consortium GTEx, Laboratory Data Analysis & Coordinating Center—Analysis Working Group, Methods groups—Analysis Working Group Statistical , et al. Genetic effects on gene expression across human tissues. Nature. 2017;550(7675):204–13. 10.1038/nature24277. 29022597 PMC5776756

[46] Võsa U, Claringbould A, Westra H-J, et al. Large-scale cis- and trans-eQTL analyses identify thousands of genetic loci and polygenic scores that regulate blood gene expression. Nat Genet. 2021;53(9):1300–10. 10.1038/s41588-021-00913-z. 34475573 PMC8432599

[47] Wu T, Hu E, Xu S, et al. clusterProfiler 4.0: a universal enrichment tool for interpreting omics data. Innovation. 2021;2(3):100141. 10.1016/j.xinn.2021.100141. 34557778 PMC8454663

[48] Xu S, Hu E, Cai Y, et al. Using clusterProfiler to characterize multiomics data. Nat Protoc. 2024;19(11):3292–320. 10.1038/s41596-024-01020-z. 39019974

[49] Szklarczyk D, Kirsch R, Koutrouli M, et al. The STRING database in 2023: protein-protein association networks and functional enrichment analyses for any sequenced genome of interest. Nucleic Acids Res. 2023;51(D1):D638–46. 10.1093/nar/gkac1000. 36370105 PMC9825434

[50] Yuan S, Ruan X, Sun Y, et al. Birth weight, childhood obesity, adulthood obesity and body composition, and gastrointestinal diseases: a mendelian randomization study. Obesity. 2023;31(10):2603–14. 10.1002/oby.23857. 37664887

[51] Chen J, Yuan S, Fu T, et al. Gastrointestinal consequences of type 2 diabetes mellitus and impaired glycemic homeostasis: a Mendelian randomization study. Diabetes Care. 2023;46(4):828–35. 10.2337/dc22-1385. 36800530 PMC10091506

[52] Ruan X, Chen J, Sun Y, et al. Depression and 24 gastrointestinal diseases: a Mendelian randomization study. Transl Psychiatry. 2023;13(1):146. 10.1038/s41398-023-02459-6. 37142593 PMC10160129

[53] Yuan S, Chen J, Ruan X, et al. Smoking, alcohol consumption, and 24 gastrointestinal diseases: Mendelian randomization analysis. eLife. 2023;12;e84051. 10.7554/eLife.84051. 36727839 PMC10017103

[54] Li Y, Duan Z. Updates in interaction of gastroesophageal reflux disease and extragastroesophageal digestive diseases. Expert Rev Gastroenterol Hepatol. 2022;16(11-12):1053–63. 10.1080/17474124.2022.2056018. 35860994

[55] Izbéki F, Rosztóczy AI, Yobuta JS, et al. Increased prevalence of gallstone disease and impaired gallbladder motility in patients with Barrett's esophagus. Dig Dis Sci. 2008;53;2268–75. 10.1007/s10620-007-0126-5. 18080764

[56] Cahan MA, Balduf L, Colton K et al. Proton pump inhibitors reduce gallbladder function. Surg Endosc. 2006;20:1364–67. 10.1007/s00464-005-0247-x. 16858534

[57] Wittenburg H, Lammert F. Genetic predisposition to gallbladder stones. Semin Liver Dis. 2007;27(1):109–21. 10.1055/s-2006-960174. 17295180

[58] Ferkingstad E, Oddsson A, Gretarsdottir S, et al. Genome-wide association meta-analysis yields 20 loci associated with gallstone disease. Nat Commun. 2018;9(1):5101. 10.1038/s41467-018-07460-y. 30504769 PMC6269469

[59] Fairfield CJ, Drake TM, Pius R, et al. Genome-wide analysis identifies gallstone-susceptibility loci including genes regulating gastrointestinal motility. Hepatology. 2022;75(5):1081–94. 10.1002/hep.32199. 34651315

[60] Fujikawa Y, Tominaga K, Fujii H, et al. High prevalence of gastroesophageal reflux symptoms in patients with non-alcoholic fatty liver disease associated with serum levels of triglyceride and cholesterol but not simple visceral obesity. Digestion. 2012;86(3):228–37. 10.1159/000341418. 22964626

[61] Fiorucci S, Distrutti E, Di Matteo F, et al. Circadian variations in gastric acid and pepsin secretion and intragastric bile acid in patients with reflux esophagitis and in healthy controls. Am J Gastroenterol. 1995;90(2):270–6. 7847299

[62] Xie Y, Blanc V, Kerr TA, et al. Decreased expression of cholesterol 7alpha-hydroxylase and altered bile acid metabolism in Apobec-1-/- mice lead to increased gallstone susceptibility. J Biol Chem. 2009;284(25):16860–71. 10.1074/jbc.M109.010173. 19386592 PMC2719322

[63] Kuo KK, Shin SJ, Chen ZC, et al. Significant association of ABCG5 604Q and ABCG8 D19H polymorphisms with gallstone disease. Br J Surg. 2008;95(8):1005–11. 10.1002/bjs.6178. 18457353

[64] Jiang Z-Y, Han T-Q, Suo G-J, et al. Polymorphisms at cholesterol 7alpha-hydroxylase, apolipoproteins B and E and low density lipoprotein receptor genes in patients with gallbladder stone disease. WJG. 2004;10(10):1508–12. 10.3748/wjg.v10.i10.1508. 15133863 PMC4656294

[65] Qayyum F, Lauridsen BK, Frikke-Schmidt R, et al. Genetic variants in CYP7A1 and risk of myocardial infarction and symptomatic gallstone disease. Eur Heart J. 2018;39(22):2106–16. 10.1093/eurheartj/ehy068. 29529257

[66] Hom XB, Lavine JE. Gastrointestinal complications of mitochondrial disease. Mitochondrion. 2004;4(5–6):601–7. 10.1016/j.mito.2004.07.014. 16120417

[67] Vedrenne V, Gowher A, De Lonlay P, et al. Mutation in PNPT1, which encodes a polyribonucleotide nucleotidyltransferase, impairs RNA import into mitochondria and causes respiratory-chain deficiency. Am Hum Genet. 2012;91(5):912–18. 10.1016/j.ajhg.2012.09.001. PMC3487136 23084291

[68] Lu C, Wei F, He X, et al. LncRNA expression in idiopathic achalasia: new insight and preliminary exploration into pathogenesis. Open Med (Wars). 2022;17(1):732–40. 10.1515/med-2022-0473. 35509690 PMC9007103

[69] Xu M, Jiang B, Man Z, et al. TRIM37 promotes gallbladder cancer proliferation by activating the wnt/β-catenin pathway via ubiquitination of Axin1. Transl Oncol. 2023;35:101732. 10.1016/j.tranon.2023.101732. 37379772 PMC10318496

[70] Wang J, Xu C, Cheng Q, et al. RNA sequencing revealed signals of evolution from gallbladder stone to gallbladder carcinoma. Front Oncol. 2020;10:823. 10.3389/fonc.2020.00823. 32547950 PMC7272658

[71] Chen X, Chen Y, Huang H-M, et al. SUN2: a potential therapeutic target in cancer. Oncol Lett. 2019;17(2):1401–8. 10.3892/ol.2018.9764. 30675193 PMC6341589

[72] Barahona Ponce C, Scherer D, Brinster R, et al. Gallstones, body mass index, C-reactive protein, and gallbladder cancer: Mendelian randomization analysis of Chilean and European genotype data. Hepatology. 2021;73(5):1783–96. 10.1002/hep.31537. 32893372

[73] Maslenkina K, Mikhaleva L, Naumenko M, et al. Signaling pathways in the pathogenesis of Barrett's esophagus and esophageal adenocarcinoma. Int J Mol Sci. 2023;24(11):9304. 10.3390/ijms24119304. 37298253 PMC10253447

[74] Ghatak S, Reveiller M, Toia L, et al. Bile salts at low pH cause dilation of intercellular spaces in in vitro stratified primary esophageal cells, possibly by modulating wnt signaling. J Gastrointest Surg. 2016;20(3):500–9. 10.1007/s11605-015-3062-2. 26715559 PMC7202037

[75] Takemaru K-I, Yamaguchi S, Lee YS, et al. Chibby, a nuclear beta-catenin-associated antagonist of the Wnt/Wingless pathway. Nature. 2003;422(6934):905–9. 10.1038/nature01570. 12712206

[76] Seki T, Gong L, Williams AJ, et al. JosD1, a membrane-targeted deubiquitinating enzyme, is activated by ubiquitination and regulates membrane dynamics, cell motility, and endocytosis. J Biol Chem. 2013;288(24):17145–55. 10.1074/jbc.M113.463406. 23625928 PMC3682520

[77] An J . GERD GWAS summary. Figshare. Dataset. 2019. 10.6084/m9.figshare.8986589.v1. (Accessed 24 January 2025).

[78] Lyu Y, Tong S, Huang W, et al. Supporting data for “Observational, Causal Relationship and Shared Genetic Basis between Cholelithiasis and gastroesophageal Reflux Disease: Evidence from a Cohort Study and Comprehensive Genetic Analysis.” GigaScience Database. 2025. 10.5524/102642. PMC11943489 40139907

