# Supplementary material for: Observational, causal relationship and shared genetic basis between cholelithiasis and gastroesophageal reflux disease: evidence from a cohort study and comprehensive genetic analysis
Source: Gigascience. 2025 Mar 26;14:giaf023. doi: 10.1093/gigascience/giaf023 (PMC11943489; doi:10.1093/gigascience/giaf023)
Supplement: giaf023_Supplemental_Files [file giaf023_supplemental_files.zip › supplementary figures.docx]

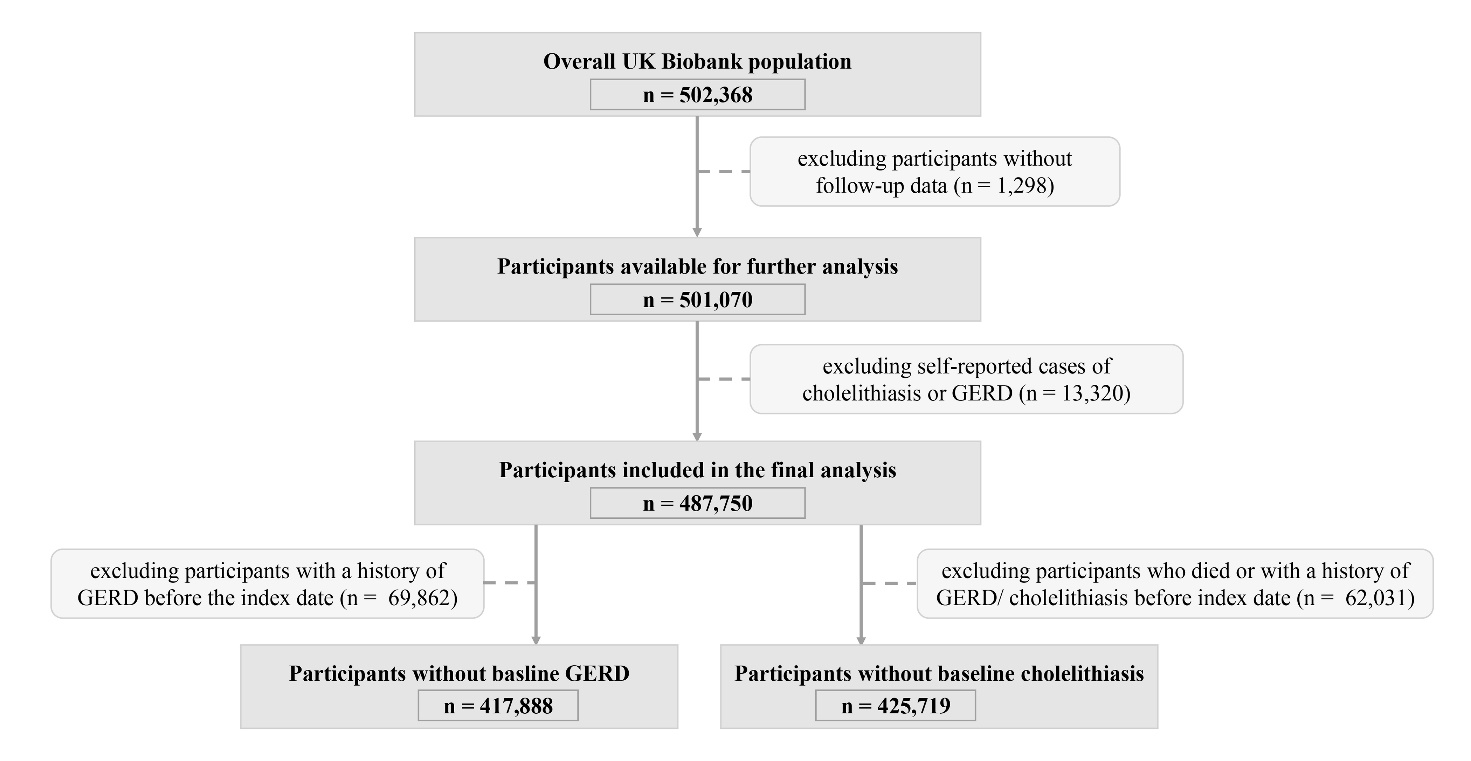


**Supplementary Figure 1.** The flowchart of the prospective cohort study

1. B.


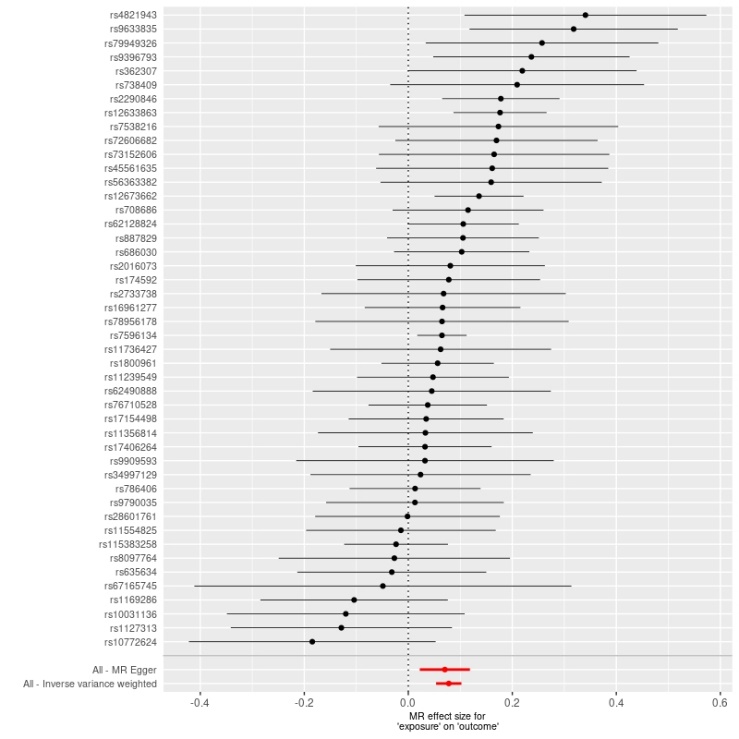

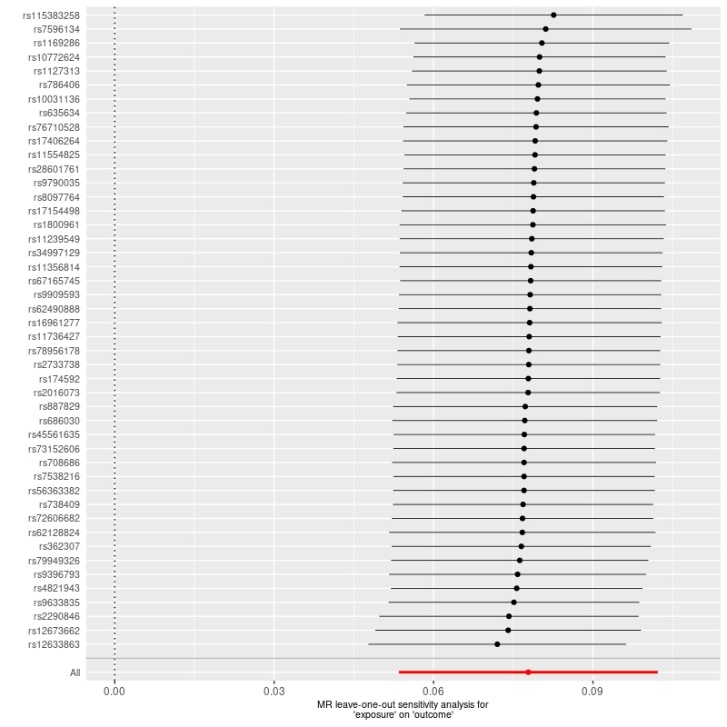


C. D.


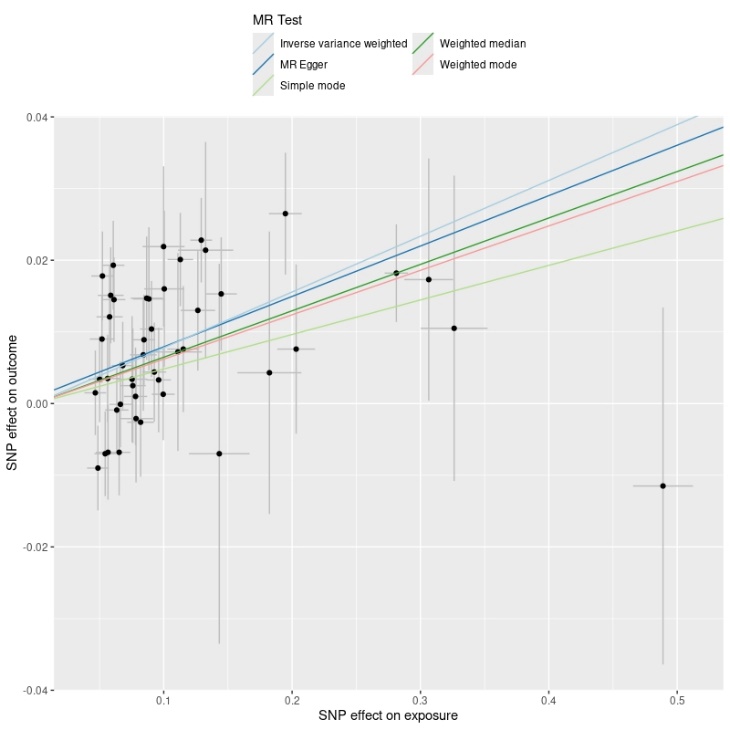

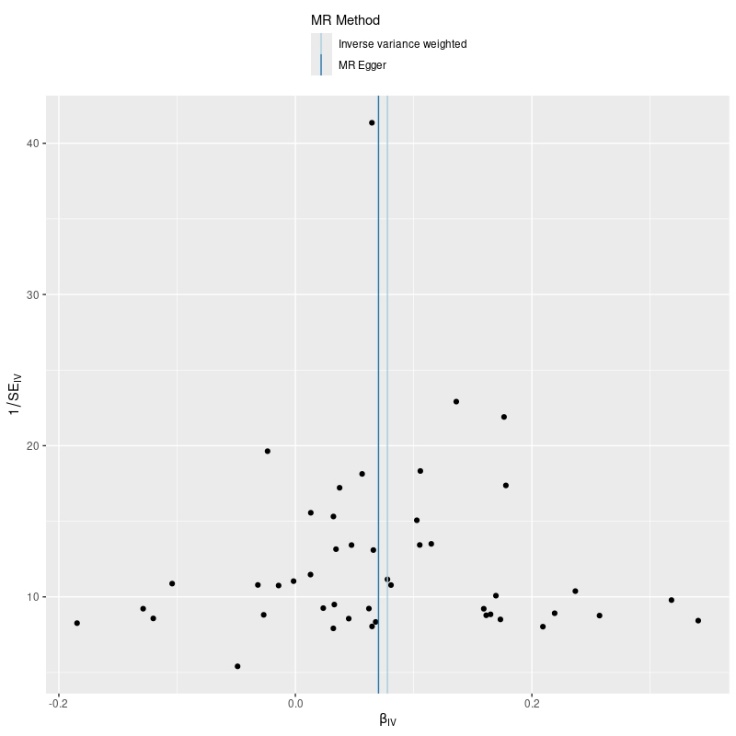


**Supplementary Figure 2.** Forest plot (A), leave-one-out analysis (B), scatter plot (C) and funnel plot (D) of the causal effect of cholelithiasis on gastroesophageal reflux disease.

1. B.


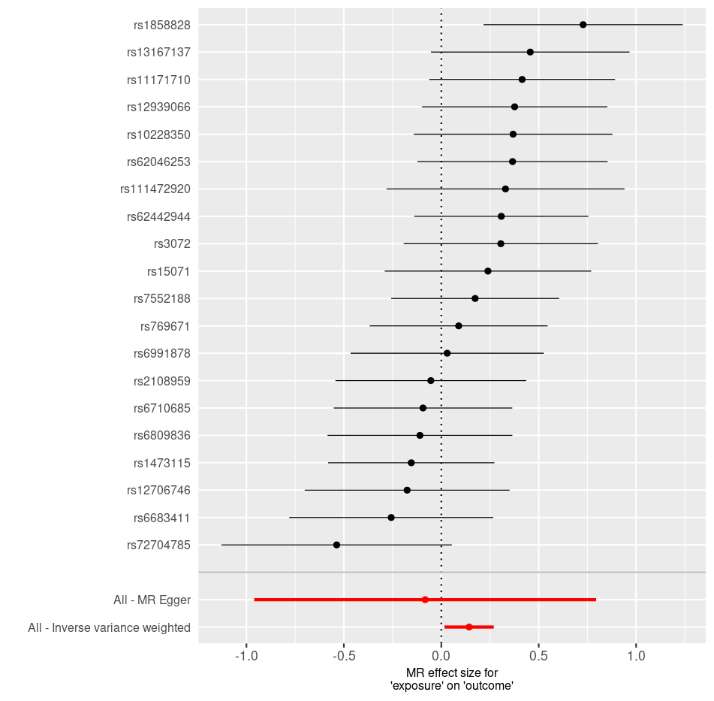

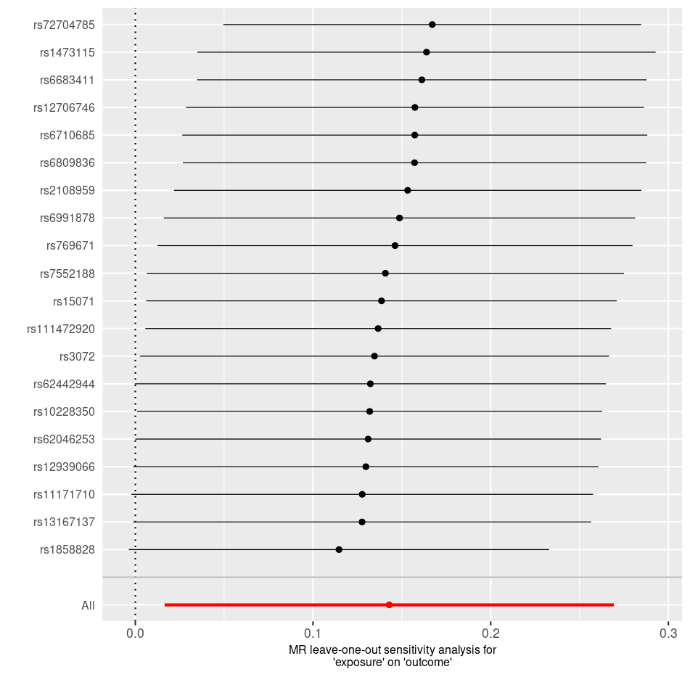


C. D.


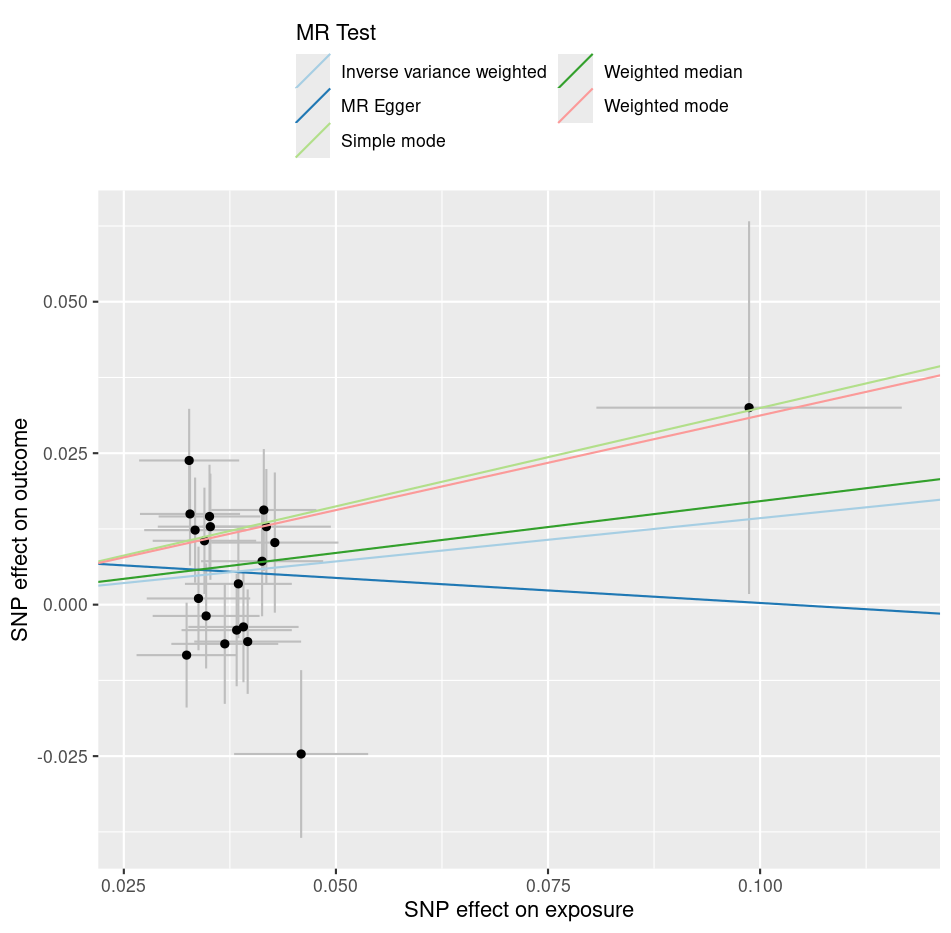

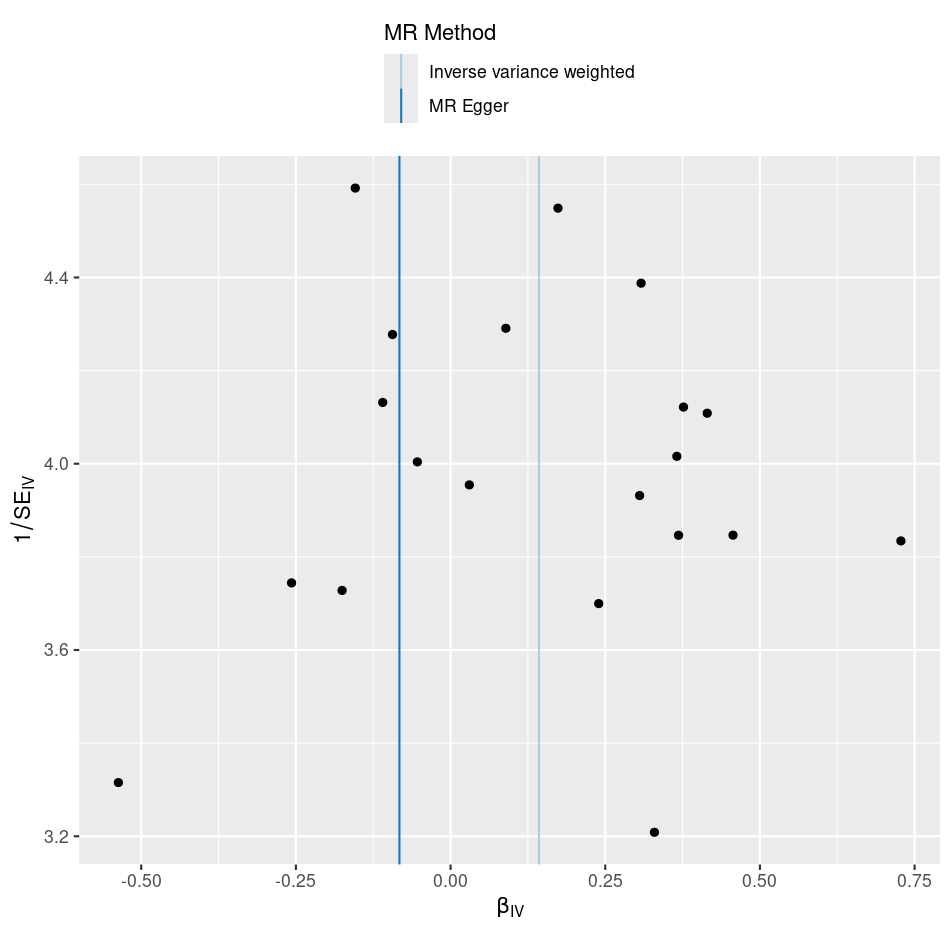


**Supplementary Figure 3.** Forest plot (A), leave-one-out analysis (B), scatter plot (C) and funnel plot (D) of the causal effect of gastroesophageal reflux disease on cholelithiasis.
