# Supplementary material for: Observational, causal relationship and shared genetic basis between cholelithiasis and gastroesophageal reflux disease: evidence from a cohort study and comprehensive genetic analysis
Source: Gigascience. 2025 Mar 26;14:giaf023. doi: 10.1093/gigascience/giaf023 (PMC11943489; doi:10.1093/gigascience/giaf023)
Supplement: giaf023_Supplemental_Files [file giaf023_supplemental_files.zip › Supplementary_Tables.docx]

# Supplementary Tables

Supplementary Table 1. Baseline characteristics of UK Biobank participants

Supplementary Table 2. Instrumental variables employed in the primary GWAS data for bidirectional Mendelian Randomization analysis

Supplementary Table 3. Summarized results of bi-directional Mendelian randomization analysis and sensitive analysis about cholelithiasis and gastroesophageal reflux disease in the primary datasets

Supplementary Table 4. Summarized results of bi-directional Mendelian randomization analysis and sensitive analysis about cholelithiasis and gastroesophageal reflux disease in the replication datasets

Supplementary Table 5. Characteristics of the regions of the genome where genetic correlation between cholelithiasis and gastroesophageal reflux disease detected by ρ_HESS and GWAS-PW

Supplementary Table 6. Independent genome-wide significant loci shared between cholelithiasis and gastroesophageal reflux disease in MTAG

Supplementary Table 7. Independent genome-wide significant loci shared between cholelithiasis and gastroesophageal reflux disease in CPASSOC

Supplementary Table 8. Enriched pathways of genes associated with pleiotropic variants based on KEGG database

Supplementary Table 9. Significantly enriched pathways of genes associated with pleiotropic variants based on GO database

Supplementary Table 10. TWAS-prioritized genes associated with cholelithiasis and gastroesophageal reflux disease

Supplementary Table 11. SMR-prioritized genes associated with cholelithiasis and gastroesophageal reflux disease

Supplementary Table 12. Enriched pathways of shared genes based on KEGG database

Supplementary Table 13. Significantly enriched pathways of shared genes based on GO database

**Supplementary Table 1. Baseline characteristics of UK Biobank participants**

**Participants without baseline GERD Participants without baseline cholelithiasis**

|  | **Cholelithiasis** | **Non-Cholelithiasis** | **GERD** | **Non-GERD** |
| --- | --- | --- | --- | --- |
| **Number of participants, n(%)** | 14 625(3.5) | 403 263(96.5) | 38 466(9.0) | 387 253(91.0) |
| **Age, mean(SD), years** | 57.8(7.9) | 56.0(8.1) | 58.1(7.7) | 55.9(8.1) |
| **Sex, female, n(%)** | 9 096(62.2) | 217 477(53.9) | 21 336(55.5) | 209 183(54.0) |
| **Ethnicity, White, n(%)** | 14 005(95.8) | 380 498(94.4) | 36 415(94.7) | 365 411(94.4) |
| **Household income (£*)** |  |  |  |  |
| <18 000, n(%) | 4 186(28.6) | 83 919(20.8) | 10 852(28.2) | 79 517(20.5) |
| 18 000-30 999, n(%) | 3 999(27.3) | 101 440(25.2) | 10 448(27.2) | 97 052(25.1) |
| 31 000-51 999, n(%) | 3 670(25.1) | 107 111(26.6) | 9 382(24.4) | 103 357(26.7) |
| 52 000-100 000, n(%) | 2 262(15.5) | 86 371(21.4) | 6 273(16.3) | 83 591(21.6) |
| >100 000, n(%) | 508(3.5) | 24 422(6.1) | 1 511(3.9) | 23 736(6.1) |
| **Deprivation index, mean(SD)** | -1.1(3.2) | -1.3(3.1) | -1.1(3.2) | -1.4(3.1) |
| **BMI, mean(SD), kg/m^2^** | 29.4(5.4) | 27.1(4.6) | 28.0(4.8) | 27.1(4.7) |
| **Alcohol consumption** |  |  |  |  |
| Daily or almost daily, n(%) | 2 144(14.7) | 84 291(20.9) | 7 475(19.4) | 80 715(20.8) |
| Three or four times a week, n(%) | 2 718(18.6) | 96 277(23.9) | 8 044(20.9) | 92 682(23.9) |
| Once or twice a week, n(%) | 3 761(25.7) | 104 933(26.0) | 9 740(25.3) | 100 943(26.1) |
| One to three times a month, n(%) | 2 008(13.7) | 44 084(10.9) | 4 355(11.3) | 42 463(11.0) |
| Special occasions only or never, n(%) | 2 398(16.4) | 43 661(10.8) | 5 149(13.4) | 41 835(10.8) |
| Never, n(%) | 1 596(10.9) | 30 017(7.4) | 3 703(9.6) | 28 615(7.4) |
| **Smoking status** |  |  |  |  |
| Never smoker, n(%) | 7 604(52.0) | 226 204(56.1) | 19 376(50.4) | 218 199(56.3) |
| Previous smoker, n(%) | 5 345(36.5) | 135 668(33.6) | 14 749(38.3) | 129 451(33.4) |
| Current smoker, n(%) | 1 676(11.5) | 41 391(10.3) | 4 341(11.3) | 39 603(10.2) |

**Physical activity, mean(SD), MET minutes/week**

2383.4(2564.9) 2673.6(2714.5) 2669.1(2802.3) 2667.8(2705.7)

# Education

| College/University, n(%) | 3 635(24.9) | 138 192(34.3) | 28 831(75.0) | 253 336(65.4) |
| --- | --- | --- | --- | --- |
| Other, n(%) | 10 990(75.1) | 265 071(65.7) | 9 635(25.0) | 133 917(34.6) |
| **Fresh fruit, mean(SD), pieces/day** | 1.9(2.7) | 1.9(2.7) | 1.9(2.7) | 1.9(2.7) |
| **Raw vegetable, mean(SD), tablespoons/day** | 1.6(3.2) | 1.7(3.4) | 1.7(3.4) | 1.7(3.4) |
| **Tea consumption, mean(SD), cups/day** | 3.2(3.7) | 3.1(3.7) | 3.3(3.7) | 3.0(3.7) |
| **Coffee consumption, mean(SD), cups/day** | 1.2(3.8) | 1.3(3.7) | 1.2(3.7) | 1.3(3.7) |
| **Comorbidities** |  |  |  |  |
| Hypertension, n(%) | 4 906(33.5) | 98 751(24.5) | 12 485(32.5) | 93 781(24.2) |
| Diabetes, n(%) | 5 088(34.8) | 101 806(25.2) | 12 885(33.5) | 96 715(25.0) |
| Renal failure, n(%) | 247(1.7) | 3 917(1.0) | 645(1.7) | 3 659(0.9) |
| Myocardial infarction, n(%) | 529(3.6) | 7 834(1.9) | 1 265(3.3) | 7 328(1.9) |
| COPD, n(%) | 423(2.9) | 5 926(1.5) | 1 215(3.2) | 5 430(1.4) |
| Asthma, n(%) | 2 045(14.0) | 44 892(11.1) | 5 940(15.4) | 42 493(11.0) |
| Peptic ulcer, n(%) | 458(3.1) | 7 428(1.8) | 1 545(4.0) | 6 796(1.8) |
| Depression, n(%) | 1 522(10.4) | 30 004(7.4) | 4 351(11.3) | 28 175(7.3) |
| Anxiety, n(%) | 641(4.4) | 13 756(3.4) | 1 962(5.1) | 12 855(3.3) |
| Dementia, n(%) | 7(<0.1) | 138(<0.1) | 19(<0.1) | 130(<0.1) |

d

*£18 000=€21 489; $23 253.

Abbreviations: GERD: gastroesophageal reflux disease; BMI: body mass index; MET: metabolic equivalent of task; COPD: chronic obstructive pulmonary disease; SD: standard deviation

**Supplementary Table 2. Instrumental variables employed in the primary GWAS data for bidirectional Mendelian Randomization analysis**

| **Outcome Exposure SNP CHR Effect** | | | | | **Reference** |  | **Exposure** |  |  | **Outcome** |  | **F** |
| --- | --- | --- | --- | --- | --- | --- | --- | --- | --- | --- | --- | --- |
|  |  |  |  | **allele** | **allele** | **Beta** | **SE** | ***P*** | **Beta** | **SE** | ***P*** | **statistics** |
| Cholelithiasis | GERD | rs6683411 | 1 | A | G | 0.032 | 0.006 | 4.82E-08 | -0.008 | 0.009 | 0.336 | 30.157 |
| Cholelithiasis | GERD | rs7552188 | 1 | T | C | 0.041 | 0.007 | 1.07E-08 | 0.007 | 0.009 | 0.430 | 32.903 |
| Cholelithiasis | GERD | rs3072 | 2 | T | C | -0.035 | 0.006 | 1.86E-08 | -0.011 | 0.009 | 0.230 | 31.987 |
| Cholelithiasis | GERD | rs1858828 | 3 | T | G | 0.033 | 0.006 | 3.23E-08 | 0.024 | 0.009 | 0.005 | 30.718 |
| Cholelithiasis | GERD | rs6762606 | 3 | T | C | -0.036 | 0.007 | 4.18E-08 | -0.001 | 0.010 | 0.900 | 30.504 |
| Cholelithiasis | GERD | rs6809836 | 3 | A | G | 0.038 | 0.007 | 3.70E-09 | -0.004 | 0.009 | 0.650 | 34.719 |
| Cholelithiasis | GERD | rs769671 | 4 | T | C | -0.039 | 0.006 | 9.28E-10 | -0.003 | 0.009 | 0.702 | 37.346 |
| Cholelithiasis | GERD | rs13167137 | 5 | T | G | -0.033 | 0.006 | 2.82E-08 | -0.015 | 0.009 | 0.079 | 30.906 |
| Cholelithiasis | GERD | rs15071 | 5 | T | C | -0.043 | 0.008 | 1.01E-08 | -0.010 | 0.012 | 0.376 | 32.566 |
| Cholelithiasis | GERD | rs72704785 | 5 | A | G | 0.046 | 0.008 | 6.81E-09 | -0.025 | 0.014 | 0.075 | 33.758 |
| Cholelithiasis | GERD | rs10228350 | 7 | A | T | -0.033 | 0.006 | 2.55E-08 | -0.012 | 0.009 | 0.156 | 30.988 |
| Cholelithiasis | GERD | rs12706746 | 7 | A | G | 0.037 | 0.006 | 4.24E-09 | -0.006 | 0.010 | 0.513 | 34.306 |
| Cholelithiasis | GERD | rs62442944 | 7 | T | G | 0.042 | 0.008 | 3.49E-08 | 0.013 | 0.010 | 0.177 | 30.250 |
| Cholelithiasis | GERD | rs6991878 | 8 | T | C | -0.034 | 0.006 | 2.71E-08 | -0.001 | 0.009 | 0.905 | 30.702 |
| Cholelithiasis | GERD | rs11171710 | 12 | A | G | 0.035 | 0.006 | 4.17E-09 | 0.015 | 0.009 | 0.088 | 34.223 |
| Cholelithiasis | GERD | rs111472920 | 13 | T | G | 0.099 | 0.018 | 4.47E-08 | 0.033 | 0.031 | 0.290 | 30.067 |
| Cholelithiasis | GERD | rs62046253 | 16 | T | C | -0.035 | 0.006 | 1.30E-08 | -0.013 | 0.009 | 0.142 | 32.233 |
| Cholelithiasis | GERD | rs12939066 | 17 | T | C | 0.042 | 0.006 | 3.13E-11 | 0.016 | 0.010 | 0.121 | 44.804 |
| Cholelithiasis | GERD | rs2108959 | 17 | C | G | 0.035 | 0.006 | 3.92E-08 | -0.002 | 0.009 | 0.830 | 30.337 |
| Cholelithiasis | GERD | rs1473115 | 19 | T | C | 0.040 | 0.006 | 3.95E-10 | -0.006 | 0.009 | 0.479 | 39.510 |
| GERD | Cholelithiasis | rs1127313 | 1 | A | G | 0.054 | 0.008 | 1.39E-10 | -0.007 | 0.006 | 0.237 | 41.178 |
| GERD | Cholelithiasis | rs7538216 | 1 | C | T | 0.052 | 0.010 | 4.76E-08 | 0.009 | 0.006 | 0.138 | 29.813 |
| GERD | Cholelithiasis | rs11356814 | 2 | A | G | 0.076 | 0.010 | 5.18E-14 | 0.003 | 0.008 | 0.756 | 56.661 |
| GERD | Cholelithiasis | rs115383258 | 2 | G | A | 0.489 | 0.023 | 7.96E-98 | -0.012 | 0.025 | 0.643 | 440.614 |
| GERD | Cholelithiasis | rs11554825 | 2 | T | C | 0.063 | 0.009 | 2.06E-12 | -0.001 | 0.006 | 0.879 | 49.423 |
| GERD | Cholelithiasis | rs17406264 | 2 | T | C | 0.326 | 0.026 | 5.08E-36 | 0.011 | 0.021 | 0.621 | 157.015 |
| GERD | Cholelithiasis | rs34997129 | 2 | T | C | 0.182 | 0.025 | 2.22E-13 | 0.004 | 0.020 | 0.826 | 53.796 |
| GERD | Cholelithiasis | rs7596134 | 2 | A | C | 0.281 | 0.009 | 1.00E-200 | 0.018 | 0.007 | 0.007 | 961.551 |
| GERD | Cholelithiasis | rs76710528 | 2 | C | T | -0.203 | 0.015 | 1.79E-43 | -0.008 | 0.012 | 0.520 | 191.144 |
| GERD | Cholelithiasis | rs786406 | 2 | G | A | 0.100 | 0.009 | 4.85E-29 | 0.001 | 0.006 | 0.834 | 125.094 |
| GERD | Cholelithiasis | rs887829 | 2 | T | C | 0.085 | 0.009 | 1.07E-22 | 0.009 | 0.006 | 0.163 | 96.149 |
| GERD | Cholelithiasis | rs12633863 | 3 | A | G | -0.129 | 0.008 | 1.08E-52 | -0.023 | 0.006 | 0.000 | 233.400 |
| GERD | Cholelithiasis | rs73152606 | 3 | A | G | -0.088 | 0.012 | 7.16E-13 | -0.015 | 0.010 | 0.144 | 51.500 |
| GERD | Cholelithiasis | rs9790035 | 3 | C | T | -0.078 | 0.009 | 5.49E-18 | -0.001 | 0.007 | 0.887 | 74.696 |
|  |  |  |  |  |  |  |  |  |  |  |  |  |

| GERD | Cholelithiasis | rs10031136 | 4 | G | A | 0.057 | 0.009 | 2.08E-09 | -0.007 | 0.007 | 0.302 | 35.894 |
| --- | --- | --- | --- | --- | --- | --- | --- | --- | --- | --- | --- | --- |
| GERD | Cholelithiasis | rs11736427 | 4 | T | A | -0.056 | 0.009 | 1.94E-09 | -0.004 | 0.006 | 0.565 | 36.029 |
| GERD | Cholelithiasis | rs2290846 | 4 | A | G | 0.113 | 0.010 | 1.49E-29 | 0.020 | 0.007 | 0.002 | 127.435 |
| GERD | Cholelithiasis | rs362307 | 4 | T | C | 0.100 | 0.016 | 1.03E-09 | 0.022 | 0.011 | 0.051 | 37.272 |
| GERD | Cholelithiasis | rs78956178 | 6 | G | T | -0.111 | 0.019 | 2.60E-09 | -0.007 | 0.014 | 0.603 | 35.465 |
| GERD | Cholelithiasis | rs9396793 | 6 | C | T | -0.061 | 0.009 | 3.55E-12 | -0.015 | 0.006 | 0.014 | 48.361 |
| GERD | Cholelithiasis | rs12673662 | 7 | G | C | -0.195 | 0.013 | 2.24E-52 | -0.027 | 0.009 | 0.002 | 231.952 |
| GERD | Cholelithiasis | rs17154498 | 7 | A | C | -0.096 | 0.009 | 3.88E-24 | -0.003 | 0.007 | 0.651 | 102.709 |
| GERD | Cholelithiasis | rs45561635 | 7 | C | A | -0.133 | 0.022 | 7.33E-10 | -0.021 | 0.015 | 0.155 | 37.932 |
| GERD | Cholelithiasis | rs79949326 | 7 | T | C | 0.059 | 0.010 | 4.75E-09 | 0.015 | 0.007 | 0.024 | 34.290 |
| GERD | Cholelithiasis | rs28601761 | 8 | G | C | 0.066 | 0.009 | 9.67E-15 | 0.000 | 0.006 | 0.983 | 59.963 |
| GERD | Cholelithiasis | rs62490888 | 8 | A | G | -0.075 | 0.012 | 1.95E-10 | -0.003 | 0.009 | 0.698 | 40.512 |
| GERD | Cholelithiasis | rs72606682 | 8 | C | T | 0.087 | 0.012 | 2.49E-12 | 0.015 | 0.009 | 0.090 | 49.056 |
| GERD | Cholelithiasis | rs635634 | 9 | C | T | -0.082 | 0.010 | 3.49E-15 | 0.003 | 0.008 | 0.732 | 61.969 |
| GERD | Cholelithiasis | rs686030 | 9 | A | C | 0.127 | 0.013 | 4.24E-21 | 0.013 | 0.008 | 0.122 | 88.859 |
| GERD | Cholelithiasis | rs11239549 | 10 | G | A | 0.093 | 0.009 | 1.73E-23 | 0.004 | 0.007 | 0.521 | 99.752 |
| GERD | Cholelithiasis | rs174592 | 11 | G | A | 0.068 | 0.009 | 1.66E-15 | 0.005 | 0.006 | 0.388 | 63.431 |
| GERD | Cholelithiasis | rs56363382 | 11 | T | C | 0.100 | 0.016 | 1.16E-10 | 0.016 | 0.011 | 0.142 | 41.532 |
| GERD | Cholelithiasis | rs9633835 | 11 | A | G | -0.061 | 0.009 | 2.88E-12 | -0.019 | 0.006 | 0.002 | 48.767 |
| GERD | Cholelithiasis | rs10772624 | 12 | A | G | -0.049 | 0.008 | 9.03E-09 | 0.009 | 0.006 | 0.129 | 33.039 |
| GERD | Cholelithiasis | rs1169286 | 12 | C | T | -0.065 | 0.009 | 4.54E-14 | 0.007 | 0.006 | 0.252 | 56.919 |
| GERD | Cholelithiasis | rs16961277 | 13 | G | A | -0.115 | 0.012 | 1.66E-20 | -0.008 | 0.009 | 0.386 | 86.155 |
| GERD | Cholelithiasis | rs2016073 | 13 | A | G | -0.084 | 0.010 | 4.44E-16 | -0.007 | 0.008 | 0.377 | 66.030 |
| GERD | Cholelithiasis | rs9909593 | 17 | G | A | -0.047 | 0.008 | 3.57E-08 | -0.002 | 0.006 | 0.799 | 30.369 |
| GERD | Cholelithiasis | rs8097764 | 18 | A | G | -0.078 | 0.013 | 3.56E-09 | 0.002 | 0.009 | 0.815 | 34.853 |
| GERD | Cholelithiasis | rs2733738 | 19 | A | G | -0.050 | 0.009 | 6.17E-09 | -0.003 | 0.006 | 0.573 | 33.781 |
| GERD | Cholelithiasis | rs62128824 | 19 | C | T | -0.145 | 0.012 | 1.19E-32 | -0.015 | 0.008 | 0.053 | 141.592 |
| GERD | Cholelithiasis | rs67165745 | 19 | A | C | -0.143 | 0.024 | 1.29E-09 | 0.007 | 0.027 | 0.791 | 36.831 |
| GERD | Cholelithiasis | rs708686 | 19 | T | C | 0.090 | 0.009 | 3.22E-24 | 0.010 | 0.007 | 0.122 | 103.081 |
| GERD | Cholelithiasis | rs1800961 | 20 | T | C | 0.306 | 0.019 | 1.14E-58 | 0.017 | 0.017 | 0.305 | 260.814 |
| GERD | Cholelithiasis | rs4821943 | 22 | G | A | 0.052 | 0.009 | 1.47E-09 | 0.018 | 0.006 | 0.004 | 36.567 |
| GERD | Cholelithiasis | rs738409 | 22 | G | C | -0.058 | 0.010 | 1.32E-08 | -0.012 | 0.007 | 0.091 | 32.295 |

Abbreviations: SNP, single nucleotide polymorphisms; CHR, chromosome, GERD, gastroesophageal reflux disease; SE, standard error.

**Supplementary Table 3. Summarized results of bi-directional Mendelian randomization analysis and sensitive analysis about cholelithiasis and gastroesophageal reflux disease in the primary datasets**

|  | | | | | **OR(95% CI)** | ***P*** | **Q** | ***P*** | **Intercept(MR-Egger)** | ***P*** |
| --- | --- | --- | --- | --- | --- | --- | --- | --- | --- | --- |
| GERD | Cholelithiasis | 104.2509 | 46 | Inverse variance weighted | 1.08(1.05,1.11) | **3.70E-10** | 59.547 | 0.072 |  |  |
| GERD | Cholelithiasis |  | 46 | MR Egger | 1.07(1.02,1.13) | 6.50E-03 | 59.377 | 0.061 | 8.89E-04 | 0.724 |
| GERD | Cholelithiasis |  | 46 | Weighted median | 1.07(1.03,1.11) | 3.78E-04 |  |  |  |  |
| GERD | Cholelithiasis |  | 46 | Weighted mode | 1.06(1.02,1.11) | 5.75E-03 |  |  |  |  |
| Cholelithiasis | GERD | 33.4332 | 20 | Inverse variance weighted | 1.15(1.02,1.31) | **2.67E-02** | 25.225 | 0.153 |  |  |
| Cholelithiasis | GERD |  | 20 | MR Egger | 0.92(0.38,2.21) | 8.56E-01 | 24.867 | 0.129 | 8.55E-03 | 0.618 |
| Cholelithiasis | GERD |  | 20 | Weighted median | 1.19(1.00,1.40) | 4.08E-02 |  |  |  |  |
| Cholelithiasis | GERD |  | 20 | Weighted mode | 1.37(1.00,1.87) | 6.56E-02 |  |  |  |  |

**Outcome Exposure F statistics* nSNPs Method Mendelian randomization Heterogeneity Pleiotropy**

* Averaged F statistics of instrument variables of cholelithiasis or gastroesophageal reflux disease. Abbreviations: nSNPs, number of single nucleotide polymorphisms used in the analysis; GERD, gastroesophageal reflux disease; OR, odds ratio; *P* , p value; 95% CI, 95% confidence interval.

# Supplementary Table 4. Summarized results of bi-directional Mendelian randomization analysis and sensitive analysis about cholelithiasis and gastroesophageal reflux disease in the replication datasets

|  | | | | **OR(95% CI)** | ***P*** | **Q** | ***P*** | **Intercept(MR-Egger)** | ***P*** |
| --- | --- | --- | --- | --- | --- | --- | --- | --- | --- |
| GERD | Cholelithiasis | 18 | Inverse variance weighted | 1.07(1.04,1.11) | **8.13E-06** | 20.789 | 0.236 |  |  |
| GERD | Cholelithiasis | 18 | MR Egger | 1.07(0.99,1.14) | 9.47E-02 | 20.722 | 0.189 | 7.45E-04 | 0.822 |
| GERD | Cholelithiasis | 18 | Weighted median | 1.10(1.05,1.14) | 2.12E-05 |  |  |  |  |
| GERD | Cholelithiasis | 18 | Weighted mode | 1.10(1.04,1.16) | 1.86E-03 |  |  |  |  |
| Cholelithiasis | GERD | 60 | Inverse variance weighted | 1.28(1.18,1.38) | **1.91E-09** | 73.584 | 0.096 |  |  |
| Cholelithiasis | GERD | 60 | MR Egger | 1.48(0.92,2.33) | 9.85E-02 | 73.072 | 0.088 | -4.82E-03 | 0.526 |
| Cholelithiasis | GERD | 60 | Weighted median | 1.29(1.16,1.43) | 3.35E-06 |  |  |  |  |
| Cholelithiasis | GERD | 60 | Weighted mode | 1.37(1.05,1.78) | 2.35E-02 |  |  |  |  |

**Outcome Exposure nSNPs Method Mendelian randomization Heterogeneity Pleiotropy**

Abbreviations: nSNPs, number of single nucleotide polymorphisms used in the analysis; GERD, gastroesophageal reflux disease; OR, odds ratio; 95% CI, 95% confidence interval; *P*, p value.

# Supplementary Table 5. Characteristics of the Regions of the Genome Where Genetic Correlation Between Cholelithiasis and Gastroesophageal Reflux Disease Was Detected by ρ_HESS and GWAS-PW

| CHR | start | end | local_rhog | se | z | ρ_HESS  pval | GWAS-PW  PPA_3 | ρ_HESS | GWAS-PW |
| --- | --- | --- | --- | --- | --- | --- | --- | --- | --- |
| 2 | 40281483 | 43309590 | 1.58E-04 | 7.40E-05 | 2.140 | 0.032 | 0.028 | **+** | **-** |
| 2 | 43309590 | 44313803 | 3.86E-04 | 1.53E-04 | 2.522 | 0.012 | 0.973 | **+** | **+** |
| 3 | 149043237 | 150252004 | 2.43E-04 | 8.31E-05 | 2.928 | 0.003 | 0.939 | **+** | **+** |
| 6 | 25684587 | 26791233 | 1.04E-04 | 5.16E-05 | 2.017 | 0.044 | 0.115 | **+** | **-** |
| 6 | 26791233 | 28017819 | 3.96E-05 | 4.31E-05 | 0.920 | 0.358 | 0.972 | **-** | **+** |
| 7 | 85572182 | 87825004 | 2.11E-04 | 7.66E-05 | 2.753 | 0.006 | 0.212 | **+** | **-** |
| 8 | 59068651 | 59728100 | 2.02E-04 | 6.09E-05 | 3.319 | 0.001 | 0.959 | **+** | **+** |
| 10 | 19716878 | 22772283 | 8.74E-05 | 5.97E-05 | 1.465 | 0.143 | 0.679 | **-** | **+** |
| 16 | 11520161 | 12613098 | 8.46E-05 | 5.70E-05 | 1.484 | 0.138 | 0.932 | **-** | **+** |
| 19 | 46102697 | 47150082 | 1.68E-04 | 5.87E-05 | 2.863 | 0.004 | 0.893 | **+** | **+** |
| 22 | 37570269 | 39307894 | 5.72E-05 | 6.16E-05 | 0.929 | 0.353 | 0.695 | **-** | **+** |

Abbreviations: se, standard error; pval, p value; ρ_HESS, rho Heritability Estimator from Summary Statistics; GWAS-PW, Pairwise-GWAS

# Supplementary Table 6. Independent genome-wide significant loci shared between cholelithiasis and gastroesophageal reflux disease in MTAG

SNP CHR BP

*P* _GWAS

*P* _MTAG *P* _CPASSOC

Gene

*Genes that interact the SNP through 3D chromatin loops in different cell types.

|  | | | Cholelithiasis | GERD | Cholelithiasis | GERD |  |
| --- | --- | --- | --- | --- | --- | --- | --- |
| rs146812426 | 2 | 43909666 | 1.88E-113 | 7.51E-02 | 1.26E-93 | 2.48E-08 | 3.68E-121 PLEKHH2 |
| rs4299376 | 2 | 44072576 | 6.82E-148 | 6.57E-03 | 5.88E-124 | 2.12E-12 | 2.10E-158 ABCG8 |
| rs6733452 | 2 | 44094845 | 1.05E-141 | 1.16E-03 | 1.63E-120 | 9.66E-14 | 1.04E-151 ABCG8 |
| rs7596134 | 2 | 44052833 | 4.08E-211 | 7.47E-03 | 8.81E-175 | 3.78E-15 | 8.31E-227 DYNC2LI1, ABCG5 |
| rs10935762 | 3 | 149216298 | 2.83E-45 | 1.51E-04 | 5.12E-42 | 1.33E-09 | 1.55E-47 TM4SF4 |
| rs4681515 | 3 | 149212076 | 2.39E-52 | 4.52E-05 | 1.35E-48 | 5.58E-11 | 3.67E-55 TM4SF4 |
| rs3922717 | 6 | 27030924 | 1.70E-05 | 5.55E-10 | 2.16E-08 | 1.99E-11 | 1.07E-13 LOC100270746* |
| rs9297994 | 8 | 59392324 | 1.21E-42 | 3.66E-05 | 2.25E-40 | 2.40E-10 | 1.06E-44 UBXN2B, CYP7A1 |

Abbreviations: SNP, single nucleotide polymorphism; CHR, chromosome; GERD, gastroesophageal reflux disease; MTAG, multi-trait

analysis of GWAS; CPASSOC, Cross-phenotype association test

# Supplementary Table 7. Independent genome-wide significant loci shared between cholelithiasis and gastroesophageal reflux disease in CPASSOC

SNP CHR BP *P* _GWAS *P* _MTAG *P* _CPASSOC

|  |  |  | Cholelithiasis | GERD | Cholelithiasis | GERD |  |
| --- | --- | --- | --- | --- | --- | --- | --- |
| rs10167227 | 2 | 56004781 | 4.00E-04 | 6.17E-06 | 9.28E-06 | 7.90E-07 | 2.93E-08 PNPT1* |
| rs6742945 | 2 | 53201324 | 1.34E-04 | 9.53E-06 | 3.38E-06 | 1.10E-06 | 1.43E-08 LOC105369165 |
| rs335208 | 5 | 122503245 | 1.23E-04 | 7.04E-06 | 2.58E-06 | 5.66E-07 | 6.47E-09 PRDM6 |
| rs72664027 | 8 | 62948007 | 1.42E-04 | 3.43E-05 | 5.03E-06 | 3.49E-06 | 3.89E-08 LINC02842 |
| rs11537754 | 16 | 570557 | 2.53E-05 | 3.36E-05 | 8.29E-07 | 2.31E-06 | 5.95E-09 RAB11FIP3 |
| rs802036 | 7 | 86977894 | 4.20E-20 | 1.05E-05 | 5.58E-21 | 5.84E-09 | 1.52E-22 CROT |
| rs12633863 | 3 | 149211512 | 1.08E-52 | 1.23E-04 | 2.03E-48 | 2.63E-10 | 1.56E-55 TM4SF4 |
| rs10935762 | 3 | 149216298 | 2.83E-45 | 1.51E-04 | 5.12E-42 | 1.33E-09 | 1.55E-47 TM4SF4 |
| rs3922717 | 6 | 27030924 | 1.70E-05 | 5.55E-10 | 2.16E-08 | 1.99E-11 | 1.07E-13 LOC100270746* |
| rs9297994 | 8 | 59392324 | 1.21E-42 | 3.66E-05 | 2.25E-40 | 2.40E-10 | 1.06E-44 UBXN2B, CYP7A1 |
| rs3738030 | 1 | 154575315 | 2.38E-06 | 8.13E-04 | 1.34E-03 | 1.65E-02 | 2.85E-08 ADAR |
| rs146439726 | 3 | 148449967 | 1.01E-09 | 9.39E-04 | 2.07E-10 | 2.42E-05 | 4.49E-11 AGTR1 |
| rs78927161 | 3 | 149208266 | 9.99E-15 | 2.32E-04 | - | - | 4.71E-16 TM4SF4 |
| rs1870514 | 4 | 151180679 | 5.01E-07 | 9.55E-04 | - | - | 6.61E-09 - |
| rs274641 | 7 | 86257939 | 1.10E-10 | 3.29E-05 | 5.08E-12 | 3.65E-07 | 7.59E-14 GRM3* |
| rs12770228 | 10 | 21783634 | 2.09E-07 | 1.42E-04 | 1.33E-08 | 6.23E-06 | 4.16E-10 MIR1915HG |
| rs7195277 | 16 | 11631291 | 6.48E-08 | 3.72E-05 | 2.52E-09 | 1.22E-06 | 3.08E-11 LITAF* |
| rs139063750 | 19 | 46312068 | 1.68E-04 | 2.19E-05 | 5.18E-06 | 2.20E-06 | 3.30E-08 RSPH6A |
| rs1800437 | 19 | 46181392 | 1.23E-07 | 1.12E-04 | - | - | 1.50E-10 GIPR |
| rs36073277 | 19 | 46298677 | 1.48E-05 | 3.16E-04 | 1.13E-06 | 2.79E-05 | 4.47E-08 RSPH6A |
| rs36120341 | 19 | 46224971 | 3.06E-15 | 6.28E-05 | 4.66E-16 | 2.50E-07 | 3.70E-17 FBXO46 |
| rs8112559 | 19 | 46390455 | 1.46E-11 | 4.51E-04 | - | - | 4.07E-13 IRF2BP1, MYPOP |
| rs5757251 | 22 | 39100128 | 5.84E-07 | 7.92E-05 | 2.79E-08 | 3.60E-06 | 5.08E-10 GTPBP1 |

Gene

*Genes that interact the SNP through 3D chromatin loops in different cell types.

Abbreviations: SNP, single nucleotide polymorphism; CHR, chromsome; GERD, gastroesophageal reflux disease; MTAG, multi-trait analysis of GWAS; CPASSOC, Cross-phenotype association test

| **Supplementary Table 8. Enriched pathways of genes associated with pleiotropic variants based on KEGG database.** | | | | | | | | | | | | | | | | | | | | |  |  |  |
| --- | --- | --- | --- | --- | --- | --- | --- | --- | --- | --- | --- | --- | --- | --- | --- | --- | --- | --- | --- | --- | --- | --- | --- |
|  |  |  |  |  |  |  |  |  |  |  |  |  |  |  |  |  |  |  |  |  |  |  |  |
| category | subcategory | ID | | Description | | GeneRatio | BgRatio | | pvalue | | p.adjust | | qvalue | | | geneID | | Count |  | |  |  |  |
| Organismal Systems | Digestive system | hsa04979 | | Cholesterol metabolism | | 3/8 | 51/8848 | | 9.90E-06 | | 0.000139 | | 8.34E-05 | | | ABCG8/ABCG5/CYP7A1 | | 3 |  | |  |  |  |
| Organismal Systems | Digestive system | hsa04976 | | Bile secretion | | 3/8 | 90/8848 | | 5.49E-05 | | 0.000385 | | 0.000231 | | | ABCG8/ABCG5/CYP7A1 | | 3 |  | |  |  |  |
| Organismal Systems | Digestive system | hsa04975 | | Fat digestion and absorption | | 2/8 | 43/8848 | | 0.000634 | | 0.002431 | | 0.001462 | | | ABCG8/ABCG5 | | 2 |  | |  |  |  |
| Environmental Information Processing | Membrane transport | hsa02010 | | ABC transporters | | 2/8 | 45/8848 | | 0.000695 | | 0.002431 | | 0.001462 | | | ABCG8/ABCG5 | | 2 |  | |  |  |  |
| Metabolism | Lipid metabolism | hsa00120 | | Primary bile acid biosynthesis | | 1/8 | 17/8848 | | 0.015274 | | 0.042766 | | 0.025724 | | | CYP7A1 | | 1 |  | |  |  |  |
| Organismal Systems | Excretory system | hsa04962 | | Vasopressin-regulated water reabsorption | | 1/8 | 44/8848 | | 0.039113 | | 0.091263 | | 0.054895 | | | DYNC2LI1 | | 1 |  | |  |  |  |
| Metabolism | Lipid metabolism | hsa00140 | | Steroid hormone biosynthesis | | 1/8 | 62/8848 | | 0.054723 | | 0.09247 | | 0.055621 | | | CYP7A1 | | 1 |  | |  |  |  |
| Metabolism | Amino acid metabolism | hsa00310 | | Lysine degradation | | 1/8 | 63/8848 | | 0.055584 | | 0.09247 | | 0.055621 | | | PRDM6 | | 1 |  | |  |  |  |
| Organismal Systems | Endocrine system | hsa03320 | | PPAR signaling pathway | | 1/8 | 76/8848 | | 0.066711 | | 0.09247 | | 0.055621 | | | CYP7A1 | | 1 |  | |  |  |  |
| Genetic Information Processing | Folding, sorting and degradation | hsa03018 | | RNA degradation | | 1/8 | 78/8848 | | 0.068413 | | 0.09247 | | 0.055621 | | | PNPT1 | | 1 |  | |  |  |  |
| Cellular Processes | Transport and catabolism | hsa04146 | | Peroxisome | | 1/8 | 83/8848 | | 0.072655 | | 0.09247 | | 0.055621 | | | CROT | | 1 |  | |  |  |  |
| Cellular Processes | Cell motility | hsa04814 | | Motor proteins | | 1/8 | 197/8848 | | 0.1649 | | 0.192384 | | 0.11572 | | | DYNC2LI1 | | 1 |  | |  |  |  |
| Human Diseases | Infectious disease: bacterial | hsa05132 | | Salmonella infection | | 1/8 | 251/8848 | | 0.205719 | | 0.206458 | | 0.124185 | | | DYNC2LI1 | | 1 |  | |  |  |  |
| Cellular Processes | Transport and catabolism | hsa04144 | | Endocytosis | | 1/8 | 252/8848 | | 0.206458 | | 0.206458 | | 0.124185 | | | RAB11FIP3 | | 1 |  | |  |  |  |
| KEGG, Kyoto Encyclopedia of Genes and Genomes | | |  | |  | | |  | |  | |  | |  |  | |  | | |  | | |  |

| **Supplementary Table 9. Significantly enriched pathways of genes associated with pleiotropic variants based on GO database.** | | | | | | | | | | | |  |  |  |  |  |  |  |
| --- | --- | --- | --- | --- | --- | --- | --- | --- | --- | --- | --- | --- | --- | --- | --- | --- | --- | --- |
|  |  |  |  |  |  |  |  |  |  |  |  |  | | | | | | |
| ID | Description | GeneRatio | BgRatio | pvalue | p.adjust | qvalue | geneID | Count | Type | | |  | |  |  |  |  |  |
| GO:0120020 | cholesterol transfer activity | 2/11 | 22/18496 | 7.38E-05 | 0.002182 | 9.36E-04 | ABCG8/ABCG5 | 2 | MF | | |  | |  |  |  |  |  |
| GO:0120015 | sterol transfer activity | 2/11 | 23/18496 | 8.08E-05 | 0.002182 | 9.36E-04 | ABCG8/ABCG5 | 2 | MF | | |  | |  |  |  |  |  |
| GO:0015248 | sterol transporter activity | 2/11 | 36/18496 | 2.00E-04 | 0.003606 | 0.001547 | ABCG8/ABCG5 | 2 | MF | | |  | |  |  |  |  |  |
| GO:0140359 | ABC-type transporter activity | 2/11 | 48/18496 | 3.57E-04 | 0.004824 | 0.002069 | ABCG8/ABCG5 | 2 | MF | | |  | |  |  |  |  |  |
| GO:0120013 | lipid transfer activity | 2/11 | 54/18496 | 4.52E-04 | 0.004886 | 0.002096 | ABCG8/ABCG5 | 2 | MF | | |  | |  |  |  |  |  |
| GO:0042626 | ATPase-coupled transmembrane transporter activity | 2/11 | 96/18496 | 0.0014223 | 0.0128 | 0.005489 | ABCG8/ABCG5 | 2 | MF | | |  | |  |  |  |  |  |
| GO:0015399 | primary active transmembrane transporter activity | 2/11 | 164/18496 | 0.0040779 | 0.030207 | 0.012954 | ABCG8/ABCG5 | 2 | MF | | |  | |  |  |  |  |  |
| GO:0005319 | lipid transporter activity | 2/11 | 172/18496 | 0.0044751 | 0.030207 | 0.012954 | ABCG8/ABCG5 | 2 | MF | | |  | |  |  |  |  |  |
| GO:0098533 | ATPase dependent transmembrane transport complex | 2/11 | 25/19886 | 8.29E-05 | 0.00431 | 0.002443 | ABCG8/ABCG5 | 2 | CC | | |  | |  |  |  |  |  |
| GO:0045177 | apical part of cell | 3/11 | 469/19886 | 0.0018676 | 0.048557 | 0.027522 | ABCG8/DYNC2LI1/ABCG5 | 3 | CC | | |  | |  |  |  |  |  |
| GO:0038183 | bile acid signaling pathway | 3/11 | 11/18870 | 2.43E-08 | 7.83E-06 | 4.39E-06 | ABCG8/ABCG5/CYP7A1 | 3 | BP | | |  | |  |  |  |  |  |
| GO:1904729 | regulation of intestinal lipid absorption | 2/11 | 10/18870 | 1.39E-05 | 0.001281 | 7.18E-04 | ABCG8/ABCG5 | 2 | BP | | |  | |  |  |  |  |  |
| GO:1904478 | regulation of intestinal absorption | 2/11 | 12/18870 | 2.03E-05 | 0.001281 | 7.18E-04 | ABCG8/ABCG5 | 2 | BP | | |  | |  |  |  |  |  |
| GO:1904779 | regulation of protein localization to centrosome | 2/11 | 12/18870 | 2.03E-05 | 0.001281 | 7.18E-04 | UBXN2B/RAB11FIP3 | 2 | BP | | |  | |  |  |  |  |  |
| GO:0042632 | cholesterol homeostasis | 3/11 | 100/18870 | 2.31E-05 | 0.001281 | 7.18E-04 | ABCG8/ABCG5/CYP7A1 | 3 | BP | | |  | |  |  |  |  |  |
| GO:0055092 | sterol homeostasis | 3/11 | 101/18870 | 2.38E-05 | 0.001281 | 7.18E-04 | ABCG8/ABCG5/CYP7A1 | 3 | BP | | |  | |  |  |  |  |  |
| GO:0060457 | negative regulation of digestive system process | 2/11 | 14/18870 | 2.80E-05 | 0.001292 | 7.24E-04 | ABCG8/ABCG5 | 2 | BP | | |  | |  |  |  |  |  |
| GO:0030299 | intestinal cholesterol absorption | 2/11 | 18/18870 | 4.70E-05 | 0.001899 | 0.001064 | ABCG8/ABCG5 | 2 | BP | | |  | |  |  |  |  |  |
| GO:0044241 | lipid digestion | 2/11 | 22/18870 | 7.09E-05 | 0.00229 | 0.001284 | ABCG8/ABCG5 | 2 | BP | | |  | |  |  |  |  |  |
| GO:0098856 | intestinal lipid absorption | 2/11 | 22/18870 | 7.09E-05 | 0.00229 | 0.001284 | ABCG8/ABCG5 | 2 | BP | | |  | |  |  |  |  |  |
| GO:0006869 | lipid transport | 4/11 | 453/18870 | 9.46E-05 | 0.002778 | 0.001557 | ABCG8/ABCG5/CROT/CYP7A1 | 4 | BP | | |  | |  |  |  |  |  |
| GO:0014850 | response to muscle activity | 2/11 | 28/18870 | 1.16E-04 | 0.003043 | 0.001706 | ABCG8/ABCG5 | 2 | BP | | |  | |  |  |  |  |  |
| GO:0055088 | lipid homeostasis | 3/11 | 175/18870 | 1.22E-04 | 0.003043 | 0.001706 | ABCG8/ABCG5/CYP7A1 | 3 | BP | | |  | |  |  |  |  |  |
| GO:0032372 | negative regulation of sterol transport | 2/11 | 31/18870 | 1.42E-04 | 0.003065 | 0.001718 | ABCG8/ABCG5 | 2 | BP | | |  | |  |  |  |  |  |
| GO:0032375 | negative regulation of cholesterol transport | 2/11 | 31/18870 | 1.42E-04 | 0.003065 | 0.001718 | ABCG8/ABCG5 | 2 | BP | | |  | |  |  |  |  |  |
| GO:0071539 | protein localization to centrosome | 2/11 | 34/18870 | 1.72E-04 | 0.003456 | 0.001937 | UBXN2B/RAB11FIP3 | 2 | BP | | |  | |  |  |  |  |  |
| GO:1905508 | protein localization to microtubule organizing center | 2/11 | 35/18870 | 1.82E-04 | 0.003456 | 0.001937 | UBXN2B/RAB11FIP3 | 2 | BP | | |  | |  |  |  |  |  |
| GO:0044058 | regulation of digestive system process | 2/11 | 37/18870 | 2.03E-04 | 0.003651 | 0.002047 | ABCG8/ABCG5 | 2 | BP | | |  | |  |  |  |  |  |
| GO:0050892 | intestinal absorption | 2/11 | 41/18870 | 2.50E-04 | 0.004235 | 0.002374 | ABCG8/ABCG5 | 2 | BP | | |  | |  |  |  |  |  |
| GO:0055090 | acylglycerol homeostasis | 2/11 | 43/18870 | 2.75E-04 | 0.004235 | 0.002374 | ABCG8/ABCG5 | 2 | BP | | |  | |  |  |  |  |  |
| GO:0070328 | triglyceride homeostasis | 2/11 | 43/18870 | 2.75E-04 | 0.004235 | 0.002374 | ABCG8/ABCG5 | 2 | BP | | |  | |  |  |  |  |  |
| GO:0032369 | negative regulation of lipid transport | 2/11 | 48/18870 | 3.43E-04 | 0.005042 | 0.002826 | ABCG8/ABCG5 | 2 | BP | | |  | |  |  |  |  |  |
| GO:0120009 | intermembrane lipid transfer | 2/11 | 54/18870 | 4.35E-04 | 0.006071 | 0.003403 | ABCG8/ABCG5 | 2 | BP | | |  | |  |  |  |  |  |
| GO:0072698 | protein localization to microtubule cytoskeleton | 2/11 | 55/18870 | 4.51E-04 | 0.006071 | 0.003403 | UBXN2B/RAB11FIP3 | 2 | BP | | |  | |  |  |  |  |  |
| GO:0044380 | protein localization to cytoskeleton | 2/11 | 59/18870 | 5.19E-04 | 0.006707 | 0.003759 | UBXN2B/RAB11FIP3 | 2 | BP | | |  | |  |  |  |  |  |
| GO:0015850 | organic hydroxy compound transport | 3/11 | 308/18870 | 6.45E-04 | 0.007768 | 0.004354 | ABCG8/ABCG5/CYP7A1 | 3 | BP | | |  | |  |  |  |  |  |
| GO:1905953 | negative regulation of lipid localization | 2/11 | 66/18870 | 6.49E-04 | 0.007768 | 0.004354 | ABCG8/ABCG5 | 2 | BP | | |  | |  |  |  |  |  |
| GO:1902017 | regulation of cilium assembly | 2/11 | 71/18870 | 7.51E-04 | 0.008546 | 0.00479 | DYNC2LI1/RAB11FIP3 | 2 | BP | | |  | |  |  |  |  |  |
| GO:0014823 | response to activity | 2/11 | 72/18870 | 7.72E-04 | 0.008546 | 0.00479 | ABCG8/ABCG5 | 2 | BP | | |  | |  |  |  |  |  |
| GO:0033344 | cholesterol efflux | 2/11 | 73/18870 | 7.94E-04 | 0.008546 | 0.00479 | ABCG8/ABCG5 | 2 | BP | | |  | |  |  |  |  |  |
| GO:0032371 | regulation of sterol transport | 2/11 | 80/18870 | 9.52E-04 | 0.009613 | 0.005388 | ABCG8/ABCG5 | 2 | BP | | |  | |  |  |  |  |  |
| GO:0032374 | regulation of cholesterol transport | 2/11 | 80/18870 | 9.52E-04 | 0.009613 | 0.005388 | ABCG8/ABCG5 | 2 | BP | | |  | |  |  |  |  |  |
| GO:0022600 | digestive system process | 2/11 | 103/18870 | 0.0015716 | 0.015382 | 0.008622 | ABCG8/ABCG5 | 2 | BP | | |  | |  |  |  |  |  |
| GO:0051051 | negative regulation of transport | 3/11 | 469/18870 | 0.0021692 | 0.020607 | 0.011551 | ABCG8/ABCG5/RAB11FIP3 | 3 | BP | | |  | |  |  |  |  |  |
| GO:0030301 | cholesterol transport | 2/11 | 128/18870 | 0.0024124 | 0.022263 | 0.012479 | ABCG8/ABCG5 | 2 | BP | | |  | |  |  |  |  |  |
| GO:0007586 | digestion | 2/11 | 134/18870 | 0.0026398 | 0.023685 | 0.013276 | ABCG8/ABCG5 | 2 | BP | | |  | |  |  |  |  |  |
| GO:0015918 | sterol transport | 2/11 | 141/18870 | 0.0029174 | 0.025468 | 0.014276 | ABCG8/ABCG5 | 2 | BP | | |  | |  |  |  |  |  |
| GO:0032368 | regulation of lipid transport | 2/11 | 153/18870 | 0.0034239 | 0.029087 | 0.016304 | ABCG8/ABCG5 | 2 | BP | | |  | |  |  |  |  |  |
| GO:0007584 | response to nutrient | 2/11 | 155/18870 | 0.0035121 | 0.029087 | 0.016304 | ABCG8/ABCG5 | 2 | BP | | |  | |  |  |  |  |  |
| GO:0015718 | monocarboxylic acid transport | 2/11 | 179/18870 | 0.0046523 | 0.037567 | 0.021058 | CROT/CYP7A1 | 2 | BP | | |  | |  |  |  |  |  |
| GO:1905952 | regulation of lipid localization | 2/11 | 183/18870 | 0.004857 | 0.038263 | 0.021448 | ABCG8/ABCG5 | 2 | BP | | |  | |  |  |  |  |  |
| GO:0031099 | regeneration | 2/11 | 191/18870 | 0.0052787 | 0.040595 | 0.022755 | TM4SF4/PNPT1 | 2 | BP | | |  | |  |  |  |  |  |
| GO:0045542 | positive regulation of cholesterol biosynthetic process | 1/11 | 10/18870 | 0.0058155 | 0.040962 | 0.02296 | CYP7A1 | 1 | BP | | |  | |  |  |  |  |  |
| GO:0106120 | positive regulation of sterol biosynthetic process | 1/11 | 10/18870 | 0.0058155 | 0.040962 | 0.02296 | CYP7A1 | 1 | BP | | |  | |  |  |  |  |  |
| GO:2000627 | positive regulation of miRNA catabolic process | 1/11 | 10/18870 | 0.0058155 | 0.040962 | 0.02296 | PNPT1 | 1 | BP | | |  | |  |  |  |  |  |
| GO:0120032 | regulation of plasma membrane bounded cell projection assembly | 2/11 | 203/18870 | 0.0059419 | 0.040962 | 0.02296 | DYNC2LI1/RAB11FIP3 | 2 | BP | | |  | |  |  |  |  |  |
| GO:0060491 | regulation of cell projection assembly | 2/11 | 205/18870 | 0.006056 | 0.040962 | 0.02296 | DYNC2LI1/RAB11FIP3 | 2 | BP | | |  | |  |  |  |  |  |
| GO:0032966 | negative regulation of collagen biosynthetic process | 1/11 | 11/18870 | 0.0063953 | 0.040962 | 0.02296 | CYP7A1 | 1 | BP | | |  | |  |  |  |  |  |
| GO:2000625 | regulation of miRNA catabolic process | 1/11 | 11/18870 | 0.0063953 | 0.040962 | 0.02296 | PNPT1 | 1 | BP | | |  | |  |  |  |  |  |
| GO:1902115 | regulation of organelle assembly | 2/11 | 218/18870 | 0.0068222 | 0.040962 | 0.02296 | DYNC2LI1/RAB11FIP3 | 2 | BP | | |  | |  |  |  |  |  |
| GO:1903828 | negative regulation of protein localization | 2/11 | 218/18870 | 0.0068222 | 0.040962 | 0.02296 | UBXN2B/RAB11FIP3 | 2 | BP | | |  | |  |  |  |  |  |
| GO:0009437 | carnitine metabolic process | 1/11 | 12/18870 | 0.0069749 | 0.040962 | 0.02296 | CROT | 1 | BP | | |  | |  |  |  |  |  |
| GO:0010713 | negative regulation of collagen metabolic process | 1/11 | 12/18870 | 0.0069749 | 0.040962 | 0.02296 | CYP7A1 | 1 | BP | | |  | |  |  |  |  |  |
| GO:0033540 | fatty acid beta-oxidation using acyl-CoA oxidase | 1/11 | 12/18870 | 0.0069749 | 0.040962 | 0.02296 | CROT | 1 | BP | | |  | |  |  |  |  |  |
| GO:0070857 | regulation of bile acid biosynthetic process | 1/11 | 12/18870 | 0.0069749 | 0.040962 | 0.02296 | CYP7A1 | 1 | BP | | |  | |  |  |  |  |  |
| GO:0007100 | mitotic centrosome separation | 1/11 | 14/18870 | 0.008133 | 0.044665 | 0.025036 | UBXN2B | 1 | BP | | |  | |  |  |  |  |  |
| GO:0035721 | intraciliary retrograde transport | 1/11 | 14/18870 | 0.008133 | 0.044665 | 0.025036 | DYNC2LI1 | 1 | BP | | |  | |  |  |  |  |  |
| GO:0090646 | mitochondrial tRNA processing | 1/11 | 14/18870 | 0.008133 | 0.044665 | 0.025036 | PNPT1 | 1 | BP | | |  | |  |  |  |  |  |
| GO:0051299 | centrosome separation | 1/11 | 15/18870 | 0.0087117 | 0.044665 | 0.025036 | UBXN2B | 1 | BP | | |  | |  |  |  |  |  |
| GO:0051791 | medium-chain fatty acid metabolic process | 1/11 | 15/18870 | 0.0087117 | 0.044665 | 0.025036 | CROT | 1 | BP | | |  | |  |  |  |  |  |
| GO:0070207 | protein homotrimerization | 1/11 | 15/18870 | 0.0087117 | 0.044665 | 0.025036 | PNPT1 | 1 | BP | | |  | |  |  |  |  |  |
| GO:0090205 | positive regulation of cholesterol metabolic process | 1/11 | 15/18870 | 0.0087117 | 0.044665 | 0.025036 | CYP7A1 | 1 | BP | | |  | |  |  |  |  |  |
| GO:1904251 | regulation of bile acid metabolic process | 1/11 | 15/18870 | 0.0087117 | 0.044665 | 0.025036 | CYP7A1 | 1 | BP | | |  | |  |  |  |  |  |
| GO:0006577 | amino-acid betaine metabolic process | 1/11 | 16/18870 | 0.00929 | 0.046885 | 0.026281 | CROT | 1 | BP | | |  | |  |  |  |  |  |
| GO:0070206 | protein trimerization | 1/11 | 17/18870 | 0.009868 | 0.049036 | 0.027487 | PNPT1 | 1 | BP | | |  | |  |  |  |  |  |
| GO, Gene Ontology Biological Process. BP, biological processes; CC, cellular components; MF, molecular functions. | | | | | | | | | |  |  | |  | |  |  |  |  |

**Supplementary Table 10. TWAS-prioritized genes associated with cholelithiasis and gastroesophageal reflux disease.**

GTEx_Tissue Ensembl gene ID Gene symbol CHR

Cholelithiasis GERD

BEST.GWAS.ID TWAS.Z TWAS.*P* TWAS.FDR BEST.GWAS.ID TWAS.Z TWAS.*P* TWAS.FDR

| Whole_Blood | ENSG00000100242.15 | SUN2 | 22 | rs1980455 | -3.629 | 2.85E-04 | 2.27E-02 | rs6001173 | -3.654 | 2.58E-04 | 3.41E-02 |
| --- | --- | --- | --- | --- | --- | --- | --- | --- | --- | --- | --- |
| Whole_Blood | ENSG00000176490.4 | DIRAS1 | 19 | rs12459507 | -3.513 | 4.44E-04 | 3.00E-02 | rs2159917 | -4.528 | 5.96E-06 | 3.21E-03 |
| Whole_Blood | ENSG00000273076.1 | - | 22 | rs1980455 | 4.346 | 1.39E-05 | 2.17E-03 | rs6001173 | 3.787 | 1.52E-04 | 2.56E-02 |
| Liver | ENSG00000100211.10 | CBY1 | 22 | rs1980455 | 3.843 | 1.22E-04 | 6.78E-03 | rs6001173 | 3.620 | 2.99E-04 | 2.02E-02 |
| Liver | ENSG00000100221.10 | JOSD1 | 22 | rs1980455 | 4.613 | 3.96E-06 | 4.10E-04 | rs6001173 | 3.850 | 1.16E-04 | 1.03E-02 |
| Liver | ENSG00000182179.12 | UBA7 | 3 | rs11712056 | -3.550 | 3.80E-04 | 1.75E-02 | rs1858828 | -4.885 | 1.04E-06 | 4.83E-04 |
| Liver | ENSG00000189067.12 | LITAF | 16 | rs3784924 | -5.487 | 4.10E-08 | 7.27E-06 | rs408665 | -3.531 | 4.15E-04 | 2.37E-02 |
| Liver | ENSG00000228274.3 | - | 22 | rs1980455 | -3.864 | 1.11E-04 | 6.36E-03 | rs6001173 | -3.590 | 3.33E-04 | 2.10E-02 |
| Esophagus_Mucosa | ENSG00000100201.20 | DDX17 | 22 | rs1980455 | 3.875 | 1.07E-04 | 1.26E-02 | rs6001173 | 3.617 | 2.98E-04 | 3.13E-02 |
| Esophagus_Mucosa | ENSG00000100221.10 | JOSD1 | 22 | rs1980455 | -3.682 | 2.31E-04 | 2.27E-02 | rs6001173 | -3.712 | 2.06E-04 | 2.60E-02 |
| Esophagus_Mucosa | ENSG00000100242.15 | SUN2 | 22 | rs1980455 | -4.222 | 2.42E-05 | 4.26E-03 | rs6001173 | -3.889 | 1.01E-04 | 1.71E-02 |
| Esophagus_Mucosa | ENSG00000189067.12 | LITAF | 16 | rs3784924 | -5.503 | 3.73E-08 | 1.80E-05 | rs408665 | -3.759 | 1.71E-04 | 2.30E-02 |
| Esophagus_Mucosa | ENSG00000228274.3 | - | 22 | rs1980455 | -3.939 | 8.19E-05 | 1.06E-02 | rs6001173 | -3.854 | 1.16E-04 | 1.84E-02 |
| Esophagus_Mucosa | ENSG00000273076.1 | - | 22 | rs1980455 | 4.527 | 5.99E-06 | 1.31E-03 | rs6001173 | 3.707 | 2.10E-04 | 2.60E-02 |
| Esophagus_Muscularis | ENSG00000100201.20 | DDX17 | 22 | rs1980455 | 3.965 | 7.34E-05 | 9.26E-03 | rs6001173 | 3.640 | 2.72E-04 | 2.59E-02 |
| Esophagus_Muscularis | ENSG00000100211.10 | CBY1 | 22 | rs1980455 | -3.894 | 9.86E-05 | 1.10E-02 | rs6001173 | -3.983 | 6.81E-05 | 1.11E-02 |
| Esophagus_Muscularis | ENSG00000127124.14 | HIVEP3 | 1 | rs702225 | -3.437 | 5.87E-04 | 3.50E-02 | rs7541348 | 3.470 | 5.20E-04 | 3.69E-02 |
| Esophagus_Muscularis | ENSG00000184949.15 | FAM227A | 22 | rs1980455 | 4.234 | 2.30E-05 | 3.61E-03 | rs6001173 | 4.058 | 4.95E-05 | 8.51E-03 |
| Esophagus_Muscularis | ENSG00000224843.6 | LINC00240 | 6 | rs6940638 | -3.495 | 4.74E-04 | 3.03E-02 | rs7763910 | -5.473 | 4.42E-08 | 1.32E-04 |
| Esophagus_Muscularis | ENSG00000228274.3 | - | 22 | rs1980455 | -3.902 | 9.53E-05 | 1.09E-02 | rs6001173 | -3.972 | 7.12E-05 | 1.14E-02 |
| Esophagus_Gastroesophageal_Junction | ENSG00000089094.18 | KDM2B | 12 | rs28728162 | 3.782 | 1.55E-04 | 1.46E-02 | rs7134248 | 3.374 | 7.41E-04 | 4.19E-02 |
| Esophagus_Gastroesophageal_Junction | ENSG00000100211.10 | CBY1 | 22 | rs1980455 | -3.594 | 3.25E-04 | 2.61E-02 | rs6001173 | -3.697 | 2.18E-04 | 2.31E-02 |
| Esophagus_Gastroesophageal_Junction | ENSG00000184949.15 | FAM227A | 22 | rs1980455 | 4.061 | 4.88E-05 | 6.77E-03 | rs6001173 | 3.926 | 8.65E-05 | 1.42E-02 |
| Esophagus_Gastroesophageal_Junction | ENSG00000204304.11 | PBX2 | 6 | rs3130342 | -3.372 | 7.46E-04 | 4.61E-02 | rs589428 | 3.921 | 8.81E-05 | 1.42E-02 |
| Esophagus_Gastroesophageal_Junction | ENSG00000224843.6 | LINC00240 | 6 | rs6940638 | -4.175 | 2.98E-05 | 4.50E-03 | rs7763910 | -4.466 | 7.95E-06 | 3.60E-03 |
| Esophagus_Gastroesophageal_Junction | ENSG00000228274.3 | - | 22 | rs1980455 | -3.902 | 9.55E-05 | 1.11E-02 | rs6001173 | -3.654 | 2.58E-04 | 2.38E-02 |
| Stomach | ENSG00000100201.20 | DDX17 | 22 | rs1980455 | 3.916 | 9.01E-05 | 8.84E-03 | rs6001173 | 3.688 | 2.26E-04 | 1.90E-02 |
| Stomach | ENSG00000101460.12 | MAP1LC3A | 20 | rs6141465 | 3.432 | 5.99E-04 | 3.69E-02 | rs6088521 | 3.548 | 3.88E-04 | 2.81E-02 |
| Stomach | ENSG00000184949.15 | FAM227A | 22 | rs1980455 | 4.035 | 5.46E-05 | 5.79E-03 | rs6001173 | 3.883 | 1.03E-04 | 1.33E-02 |
| Stomach | ENSG00000273076.1 | - | 22 | rs1980455 | 4.651 | 3.30E-06 | 5.64E-04 | rs6001173 | 3.915 | 9.04E-05 | 1.20E-02 |

Abbreviations: GERD: gastroesophageal reflux disease; TWAS: Transcriptome-wide association studies; CHR: chromosome; TWAS.FDR: TWAS false discovery rate; TWAS_Z: TWAS Z-score. GTEx: expression quantitative traits loci (eQTL) data from GTEx v8 (Genotype-Tissue Expression, version 8)

**Supplementary Table 11. SMR-prioritized genes associated with cholelithiasis and gastroesophageal reflux disease.**

probeID Disease ProbeChr Gene Probe_bp topSNP topSNP_chr topSNP_bp A1 A2 Freq b_SMR se_SMR *p* _SMR FDR_SMR *p* _HEIDI nsnp_HEIDI b_GWAS se_GWAS *p* _GWAS b_eQTL se_eQTL *p* _eQTL

| *GTEx (Esophagus Muscularis)* |  | | | | | | | | | | | | | | | | | | | | |
| --- | --- | --- | --- | --- | --- | --- | --- | --- | --- | --- | --- | --- | --- | --- | --- | --- | --- | --- | --- | --- | --- |
| ENSG00000100211 Cholelithiasis | 22 | **CBY1** | 39052641 | rs4821810 | 22 | 39072098 | G | C | 0.389 | -0.094 | 0.025 | **1.85E-04** | **0.035** | **0.853** | 20 | 0.026 | 0.006 | 6.24E-05 | -0.271 | 0.026 | 6.58E-26 |
| ENSG00000100211 GERD | 22 | **CBY1** | 39052641 | rs4821810 | 22 | 39072098 | G | C | 0.389 | -0.128 | 0.035 | **2.55E-04** | **0.043** | **0.074** | 20 | 0.035 | 0.009 | 9.63E-05 | -0.271 | 0.026 | 6.58E-26 |
| *GTEx (Esophagus Mucosa)* |  |  |  |  |  |  |  |  |  |  |  |  |  |  |  |  |  |  |  |  |  |
| ENSG00000100221 Cholelithiasis | 22 | **JOSD1** | 39081548 | rs5750629 | 22 | 38985065 | C | T | 0.362 | -0.053 | 0.013 | **8.04E-05** | **0.045** | **0.977** | 20 | 0.026 | 0.006 | 4.57E-05 | -0.494 | 0.030 | 2.60E-60 |
| ENSG00000100221 GERD | 22 | **JOSD1** | 39081548 | rs5750629 | 22 | 38985065 | C | T | 0.362 | -0.069 | 0.019 | **1.97E-04** | **0.032** | **0.303** | 20 | 0.034 | 0.009 | 1.32E-04 | -0.494 | 0.030 | 2.60E-60 |
| ENSG00000125755 Cholelithiasis | 19 | SYMPK 46318668 rs111386720 | | | 19 | 46302145 | A | G | 0.069 | 0.098 | 0.027 | **2.12E-04** | **0.048** | **0.140** | 18 | -0.040 | 0.010 | 6.47E-05 | -0.406 | 0.041 | 2.26E-23 |
| ENSG00000125755 GERD | 19 | SYMPK 46318668 rs111386720 | | | 19 | 46302145 | A | G | 0.069 | 0.108 | 0.030 | **3.72E-04** | **0.046** | **0.029** | 17 | -0.044 | 0.012 | 1.39E-04 | -0.406 | 0.041 | 2.26E-23 |
| *eQTLGen* |  |  | | |  |  |  |  |  |  |  |  |  |  |  |  |  |  |  |  |  |
| ENSG00000187837 Cholelithiasis | 6 | H1-2 | 26056333 | rs807214 | 6 | 26061769 | G | C | 0.230 | -0.202 | 0.057 | **3.80E-04** | **0.039** | **0.024** | 20 | 0.032 | 0.009 | 3.00E-04 | -0.160 | 0.008 | 2.46E-83 |
| ENSG00000187837 GERD | 6 | H1-2 | 26056333 | rs807214 | 6 | 26061769 | G | C | 0.230 | -0.162 | 0.040 | **5.43E-05** | **0.020** | **0.183** | 20 | 0.026 | 0.006 | 3.58E-05 | -0.160 | 0.008 | 2.46E-83 |
| ENSG00000100242 Cholelithiasis | 22 | **SUN2** | 39160439 | rs9607557 | 22 | 39040279 | A | G | 0.397 | -0.094 | 0.021 | **6.27E-06** | **0.002** | **0.548** | 20 | 0.040 | 0.009 | 5.76E-06 | -0.427 | 0.008 | 0 |
| ENSG00000100242 GERD | 22 | **SUN2** | 39160439 | rs9607557 | 22 | 39040279 | A | G | 0.397 | -0.059 | 0.015 | **6.25E-05** | **0.022** | **0.430** | 20 | 0.025 | 0.006 | 5.45E-05 | -0.427 | 0.008 | 0 |

Abbreviations: GERD: gastroesophageal reflux disease; SMR: Summary data-based Mendelian Randomisation; chr: chromosome; Freq: frequency of A1; FDR_SMR: false discovery rate; p_HEIDI: P value after heterogeneity in dependent instruments (HEIDI) test; nsnp: number of SNPs; b_GWAS: beta in the GWAS summary data; se_GWAS: standard error in the GWAS summary data; b_eQTL: beta in the eQTL data; se_GWAS: standard error in the eQTL data; GTEx: expression quantitative traits loci (eQTL) data from GTEx v8 (Genotype-Tissue Expression, version 8). eQTLGen: cis-eQTL data of whole blood from eQTLGen consortium.

| **Supplementary Table 12. Enriched pathways of shared genes based on KEGG database.** | | | | | | | | | | |  |
| --- | --- | --- | --- | --- | --- | --- | --- | --- | --- | --- | --- |
|  |  |  |  |  |  |  |  |  |  |  |  |
| category | subcategory | ID | Description | GeneRatio | BgRatio | pvalue | p.adjust | qvalue | geneID | Count |  |
| Environmental Information Processing | Signal transduction | hsa04310 | Wnt signaling pathway | 1/2 | 174/8848 | 0.038946 | 0.051757 | 0.02724 | CBY1 | 1 |  |
| NA | NA | hsa04820 | Cytoskeleton in muscle cells | 1/2 | 232/8848 | 0.051757 | 0.051757 | 0.02724 | SUN2 | 1 |  |
| KEGG, Kyoto Encyclopedia of Genes and Genomes | | |  |  |  |  |  |  |  |  |  |

| **Supplementary Table 13. Significantly enriched pathways of shared genes based on GO database.** | | | | | | | | | |  |
| --- | --- | --- | --- | --- | --- | --- | --- | --- | --- | --- |
|  |  |  |  |  |  |  |  |  |  |  |
| ID | Description | GeneRatio | BgRatio | pvalue | p.adjust | qvalue | geneID | Count | Type |  |
| GO:0005521 | lamin binding | 1/3 | 15/18496 | 0.002431 | 0.024311 | 0.005118 | SUN2 | 1 | MF |  |
| GO:0043495 | protein-membrane adaptor activity | 1/3 | 37/18496 | 0.00599 | 0.029948 | 0.006305 | SUN2 | 1 | MF |  |
| GO:0008013 | beta-catenin binding | 1/3 | 85/18496 | 0.013724 | 0.033565 | 0.007066 | CBY1 | 1 | MF |  |
| GO:0004843 | cysteine-type deubiquitinase activity | 1/3 | 108/18496 | 0.017416 | 0.033565 | 0.007066 | JOSD1 | 1 | MF |  |
| GO:0101005 | deubiquitinase activity | 1/3 | 114/18496 | 0.018378 | 0.033565 | 0.007066 | JOSD1 | 1 | MF |  |
| GO:0019783 | ubiquitin-like protein peptidase activity | 1/3 | 125/18496 | 0.020139 | 0.033565 | 0.007066 | JOSD1 | 1 | MF |  |
| GO:0008234 | cysteine-type peptidase activity | 1/3 | 185/18496 | 0.029709 | 0.042441 | 0.008935 | JOSD1 | 1 | MF |  |
| GO:0034992 | microtubule organizing center attachment site | 1/3 | 11/19886 | 0.001659 | 0.007049 | 0.001746 | SUN2 | 1 | CC |  |
| GO:0034993 | meiotic nuclear membrane microtubule tethering complex | 1/3 | 11/19886 | 0.001659 | 0.007049 | 0.001746 | SUN2 | 1 | CC |  |
| GO:0106083 | nuclear membrane protein complex | 1/3 | 11/19886 | 0.001659 | 0.007049 | 0.001746 | SUN2 | 1 | CC |  |
| GO:0106094 | nuclear membrane microtubule tethering complex | 1/3 | 11/19886 | 0.001659 | 0.007049 | 0.001746 | SUN2 | 1 | CC |  |
| GO:0005637 | nuclear inner membrane | 1/3 | 57/19886 | 0.008575 | 0.029154 | 0.007221 | SUN2 | 1 | CC |  |
| GO:0000794 | condensed nuclear chromosome | 1/3 | 81/19886 | 0.012171 | 0.034483 | 0.008541 | SUN2 | 1 | CC |  |
| GO:0005814 | centriole | 1/3 | 157/19886 | 0.0235 | 0.048592 | 0.012035 | CBY1 | 1 | CC |  |
| GO:0000781 | chromosome, telomeric region | 1/3 | 171/19886 | 0.025577 | 0.048592 | 0.012035 | SUN2 | 1 | CC |  |
| GO:0036064 | ciliary basal body | 1/3 | 172/19886 | 0.025725 | 0.048592 | 0.012035 | CBY1 | 1 | CC |  |
| GO:0033504 | floor plate development | 1/3 | 11/18870 | 0.001748 | 0.034848 | 0.007216 | CBY1 | 1 | BP |  |
| GO:0099515 | actin filament-based transport | 1/3 | 21/18870 | 0.003335 | 0.034848 | 0.007216 | SUN2 | 1 | BP |  |
| GO:0051457 | maintenance of protein location in nucleus | 1/3 | 23/18870 | 0.003652 | 0.034848 | 0.007216 | SUN2 | 1 | BP |  |
| GO:0007097 | nuclear migration | 1/3 | 25/18870 | 0.00397 | 0.034848 | 0.007216 | SUN2 | 1 | BP |  |
| GO:0021801 | cerebral cortex radial glia-guided migration | 1/3 | 27/18870 | 0.004287 | 0.034848 | 0.007216 | SUN2 | 1 | BP |  |
| GO:0022030 | telencephalon glial cell migration | 1/3 | 27/18870 | 0.004287 | 0.034848 | 0.007216 | SUN2 | 1 | BP |  |
| GO:0051642 | centrosome localization | 1/3 | 32/18870 | 0.005079 | 0.034848 | 0.007216 | SUN2 | 1 | BP |  |
| GO:0061842 | microtubule organizing center localization | 1/3 | 32/18870 | 0.005079 | 0.034848 | 0.007216 | SUN2 | 1 | BP |  |
| GO:0051647 | nucleus localization | 1/3 | 33/18870 | 0.005238 | 0.034848 | 0.007216 | SUN2 | 1 | BP |  |
| GO:0021799 | cerebral cortex radially oriented cell migration | 1/3 | 36/18870 | 0.005713 | 0.034848 | 0.007216 | SUN2 | 1 | BP |  |
| GO:0072595 | maintenance of protein localization in organelle | 1/3 | 41/18870 | 0.006504 | 0.03607 | 0.007469 | SUN2 | 1 | BP |  |
| GO:0021795 | cerebral cortex cell migration | 1/3 | 48/18870 | 0.007612 | 0.036955 | 0.007652 | SUN2 | 1 | BP |  |
| GO:0051289 | protein homotetramerization | 1/3 | 59/18870 | 0.009351 | 0.036955 | 0.007652 | CBY1 | 1 | BP |  |
| GO:0008347 | glial cell migration | 1/3 | 60/18870 | 0.009509 | 0.036955 | 0.007652 | SUN2 | 1 | BP |  |
| GO:0022029 | telencephalon cell migration | 1/3 | 61/18870 | 0.009667 | 0.036955 | 0.007652 | SUN2 | 1 | BP |  |
| GO:0021885 | forebrain cell migration | 1/3 | 64/18870 | 0.010141 | 0.036955 | 0.007652 | SUN2 | 1 | BP |  |
| GO:0032507 | maintenance of protein location in cell | 1/3 | 65/18870 | 0.010299 | 0.036955 | 0.007652 | SUN2 | 1 | BP |  |
| GO:0072384 | organelle transport along microtubule | 1/3 | 89/18870 | 0.014084 | 0.044876 | 0.009293 | SUN2 | 1 | BP |  |
| GO:0051262 | protein tetramerization | 1/3 | 90/18870 | 0.014241 | 0.044876 | 0.009293 | CBY1 | 1 | BP |  |
| GO:0045185 | maintenance of protein location | 1/3 | 93/18870 | 0.014713 | 0.044876 | 0.009293 | SUN2 | 1 | BP |  |
| GO:0016579 | protein deubiquitination | 1/3 | 114/18870 | 0.018016 | 0.049262 | 0.010201 | JOSD1 | 1 | BP |  |
| GO:0021987 | cerebral cortex development | 1/3 | 127/18870 | 0.020056 | 0.049262 | 0.010201 | SUN2 | 1 | BP |  |
| GO:0007052 | mitotic spindle organization | 1/3 | 130/18870 | 0.020527 | 0.049262 | 0.010201 | SUN2 | 1 | BP |  |
| GO:0055007 | cardiac muscle cell differentiation | 1/3 | 131/18870 | 0.020684 | 0.049262 | 0.010201 | CBY1 | 1 | BP |  |
| GO:0030048 | actin filament-based movement | 1/3 | 133/18870 | 0.020997 | 0.049262 | 0.010201 | SUN2 | 1 | BP |  |
| GO:0070646 | protein modification by small protein removal | 1/3 | 133/18870 | 0.020997 | 0.049262 | 0.010201 | JOSD1 | 1 | BP |  |
| GO:0090090 | negative regulation of canonical Wnt signaling pathway | 1/3 | 140/18870 | 0.022094 | 0.049498 | 0.01025 | CBY1 | 1 | BP |  |
| GO:0006997 | nucleus organization | 1/3 | 144/18870 | 0.02272 | 0.049498 | 0.01025 | SUN2 | 1 | BP |  |
| GO, Gene Ontology Biological Process. BP, biological processes; CC, cellular components; MF, molecular functions. | | | | | |  |  |  |  |  |
