# Supplementary material for: Observational, causal relationship and shared genetic basis between cholelithiasis and gastroesophageal reflux disease: evidence from a cohort study and comprehensive genetic analysis
Source: Gigascience. 2025 Mar 26;14:giaf023. doi: 10.1093/gigascience/giaf023 (PMC11943489; doi:10.1093/gigascience/giaf023)

GigaScience

Observational, Causal Relationship and Shared Genetic Basis Between Cholelithiasis and Gastroesophageal Reflux Disease: Evidence from a Cohort Study and Comprehensive Genetic Analysis

--Manuscript Draft--

**Manuscript Number:**	GIGA-D-24-00123
**Full Title:**	Observational, Causal Relationship and Shared Genetic Basis Between Cholelithiasis and Gastroesophageal Reflux Disease: Evidence from a Cohort Study and Comprehensive Genetic Analysis
**Article Type:**	Research

Funding Information:

National Natural Science Foundation of China (82171698, 81300279, 81741067)	Professor Hao Chen
National Natural Science Foundation of China (82170561)	Professor Weihong Sha
Science Fund for Distinguished Young Scholars of Jiangsu Province (2021B1515020003)	Professor Hao Chen
Natural Science Foundation of Guangdong Province (2022A1515012081)	Professor Weihong Sha
High-level Hospital Construction Project of Guangdong Provincial People's Hospital (KJ012019099, KJ012021143, KY012021183)	Professor Hao Chen
High-level Hospital Construction Project of Guangdong Provincial People's Hospital (DFJH201803)	Professor Weihong Sha

Abstract:

Objective

Cholelithiasis and gastroesophageal reflux disease (GERD) contributed to significant health concern. We aimed to investigate the potential observational, causal and genetic relationships between cholelithiasis and GERD.

Design

The observational correlations were assessed based on the prospective cohort study from UK Biobank. Then, by leveraging the genome-wide summary statistics of cholelithiasis (N = 334,277) and GERD (N = 332,601), the bidirectional causal associations was evaluated using Mendelian randomization (MR) analysis. Subsequently, a series of genetic analyses was used to assess the genetic correlation, shared loci and genes between cholelithiasis and GERD.

Results

The prospective cohort analyses revealed a significant increased risk of GERD in individuals with cholelithiasis (Hazard ratio (HR) = 1.99, 95% confidence interval (CI) = 1.89-2.10) and a higher risk of cholelithiasis among GERD patients (HR = 2.30, 95% CI = 2.18-2.44). The MR study indicated the causal effect of genetic liability to cholelithiasis on the incidence of GERD (Odds ratio (OR) = 1.07, 95% CI = 1.05-1.10) and the causal effect of genetic predicted GERD on cholelithiasis (IVW OR = 1.15, 95% CI = 1.02-1.30). Besides, cholelithiasis and GERD exhibited a strong genetic association. Cross-trait meta-analyses identified 5 novel independent loci shared between cholelithiasis and GERD. Three shared genes including SUN2, CBY1, and JOSD1 were further identified as novel risk genes.

Conclusion

The elucidation of the shared genetic basis underlying the phenotypic relationship of

Powered by Editorial Manager® and ProduXion Manager® from Aries Systems Corporation

	these two complex phenotypes offers new insights into the intrinsic linkage between cholelithiasis and GERD, providing a novel research direction for future therapeutic strategy and risk prediction.
**Corresponding Author:**	Hao Chen<br>Guangdong Provincial People's Hospital Affiliated to Southern Medical University: Guangdong Provincial People's Hospital<br>Guangzhou, Guangdong CHINA
**Corresponding Author Secondary Information:**	
**Corresponding Author's Institution:**	Guangdong Provincial People's Hospital Affiliated to Southern Medical University: Guangdong Provincial People's Hospital
**Corresponding Author's Secondary Institution:**	
**First Author:**	Yanlin Lyu
**First Author Secondary Information:**	
**Order of Authors:**	Yanlin Lyu
	Shuangshuang Tong
	Wentao Huang
	Yuying Ma
	Ruijie Zeng
	Rui Jiang
	Ruibang Luo
	Felix W Leung
	Qizhou Lian
	Weihong Sha
	Hao Chen
**Order of Authors Secondary Information:**	
**Additional Information:**	
**Question**	**Response**
Are you submitting this manuscript to a special series or article collection?	No
**Experimental design and statistics**<br><br>Full details of the experimental design and statistical methods used should be given in the Methods section, as detailed in our Minimum Standards Reporting Checklist. Information essential to interpreting the data presented should be made available in the figure legends.<br><br>Have you included all the information requested in your manuscript?	Yes

**Resources**<br><br>A description of all resources used, including antibodies, cell lines, animals and software tools, with enough information to allow them to be uniquely identified, should be included in the Methods section. Authors are strongly encouraged to cite Research Resource Identifiers (RRIDs) for antibodies, model organisms and tools, where possible.<br><br>Have you included the information requested as detailed in our Minimum Standards Reporting Checklist?	Yes
**Availability of data and materials**<br><br>All datasets and code on which the conclusions of the paper rely must be either included in your submission or deposited in publicly available repositories (where available and ethically appropriate), referencing such data using a unique identifier in the references and in the “Availability of Data and Materials” section of your manuscript.<br><br>Have you have met the above requirement as detailed in our Minimum Standards Reporting Checklist?	Yes

Click here to access/download;Manuscript;cholelithiasis-GERD_manuscript.docx

Observational, Causal Relationship and Shared Genetic Basis Between Cholelithiasis and Gastroesophageal Reflux Disease: Evidence from a Cohort Study and Comprehensive Genetic Analysis

Yanlin Lyu[1,2,3†], Shuangshuang Tong[1, 3†], Wentao Huang[1,2†], Yuying Ma[1,2†], Ruijie Zeng[1,3], Rui Jiang[1,4], Ruibang Luo[5], Felix W Leung[6,7*], Qizhou Lian[8,9,10*], Weihong Sha[1,2,3,4*], Hao Chen[1,2,3,4*]

[1]Department of Gastroenterology, Guangdong Provincial People's Hospital (Guangdong Academy of Medical Sciences), Southern Medical University, Guangzhou 510080, China

[2]The Second School of Clinical Medicine, Southern Medical University, Guangzhou 510515, China

[3]Shantou University Medical College, Shantou University, Shantou 515041, China

[4]School of Medicine, South China University of Technology, Guangzhou 510006, China

[5]Department of Computer Science, The University of Hong Kong, Hong Kong

[6]Sepulveda Ambulatory Care Center, VA Greater Los Angeles Healthcare System, Los Angeles 91343, California, USA.

[7]University of California Los Angeles David Geffen School of Medicine, Los Angeles 90095, California, USA.

[8]Faculty of Synthetic Biology, Shenzhen Institute of Advanced Technology, Chinese Academy of Sciences, Shenzhen, China

[9]Cord Blood Bank, Guangzhou Institute of Eugenics and Perinatology, Guangzhou

Women and Children's Medical Center, Guangzhou Medical University, Guangzhou, China

[10]State Key Laboratory of Pharmaceutical Biotechnology, The University of Hong Kong, SAR, China

***Corresponding authors:**

Prof. Felix W Leung,
University of California Los Angeles David Geffen School of Medicine, Los Angeles 90095, California, USA.
E-mail: felixleung@socal.rr.com

Prof. Qizhou Lian,
State Key Laboratory of Pharmaceutical Biotechnology, The University of Hong Kong, SAR, China
E-mail: qzlian@hku.hk

Prof. Weihong Sha,
Department of Gastroenterology, Guangdong Provincial People's Hospital (Guangdong Academy of Medical Sciences), Southern Medical University, Guangzhou 510080, China.
E-mail: shaweihong@gdph.org.cn

Prof. Hao Chen,
Department of Gastroenterology, Guangdong Provincial People's Hospital (Guangdong Academy of Medical Sciences), Southern Medical University, Guangzhou 510080, China.
E-mail: chenhao@gdph.org.cn

†: These authors contributed equally to this work

Abstract

Objective

Cholelithiasis and gastroesophageal reflux disease (GERD) contributed to significant health concern. We aimed to investigate the potential observational, causal and genetic relationships between cholelithiasis and GERD.

Design

The observational correlations were assessed based on the prospective cohort study from UK Biobank. Then, by leveraging the genome-wide summary statistics of cholelithiasis (N = 334,277) and GERD (N = 332,601), the bidirectional causal associations was evaluated using Mendelian randomization (MR) analysis. Subsequently, a series of genetic analyses was used to assess the genetic correlation, shared loci and genes between cholelithiasis and GERD.

Results

The prospective cohort analyses revealed a significant increased risk of GERD in individuals with cholelithiasis (Hazard ratio (HR) = 1.99, 95% confidence interval (CI) = 1.89-2.10) and a higher risk of cholelithiasis among GERD patients (HR = 2.30, 95% CI = 2.18-2.44). The MR study indicated the causal effect of genetic liability to cholelithiasis on the incidence of GERD (Odds ratio (OR) = 1.07, 95% CI = 1.05-1.10) and the causal effect of genetic predicted GERD on cholelithiasis (IVW OR = 1.15, 95% CI = 1.02-1.30). Besides, cholelithiasis and GERD exhibited a strong genetic association. Cross-trait meta-analyses identified 5 novel independent loci shared between cholelithiasis and GERD. Three shared genes including *SUN2*, *CBY1*, and *JOSD1* were further identified as novel risk genes.

Conclusion

The elucidation of the shared genetic basis underlying the phenotypic relationship of these two complex phenotypes offers new insights into the intrinsic linkage between cholelithiasis and GERD, providing a novel research direction for future therapeutic strategy and risk prediction.

Keywords: cholelithiasis, gastroesophageal reflux disease, cohort study, Mendelian randomization, genetic analyses, causal association, shared genetic basis

Introduction

Cholelithiasis, a condition characterized by lithic deposits of either cholesterol or bilirubin in the gallbladder or the bile ducts, is one of the most prevalent digestive disorders, imposing significant socioeconomic burden [1]. Cholelithiasis affects nearly 20% of the adult population worldwide, with a continuously rising incidence rate [1, 2]. The development of cholelithiasis involves intricate mechanisms, encompassing genetic and environmental factors, and their interactions. [1, 3] The gastrointestinal defects in patients with cholelithiasis have raised widespread concerns and require further exploration [1, 4].

Gastroesophageal reflux disease (GERD) is a common gastrointestinal disorder typically characterized by recurrent heartburn and regurgitation [5, 6]. This condition could pose a substantial public health challenge, owing to its association with a spectrum of subsequent severe complications, including Barrett's esophagus, esophageal stenosis, and esophageal adenocarcinoma [7]. Therefore, early identification and vigilant monitoring of individuals at high risk for GERD can facilitate timely intervention, potentially mitigating the severity of the disease and decreasing the risk of GERD and GERD-related complications.

Several studies have investigated the correlation between cholelithiasis and the risk of GERD [8-11]. Nonetheless, the existing findings have been inconsistent and insufficient, lacking support from prospective studies. For instance, a retrospective, observational study involving 1,381,004 individuals with gallstone disease found that 40% of the patients had concurrent GERD [11]. On the contrary, a case-control study, comprising 790 cases and 407 controls, demonstrated no associations between the presence of cholelithiasis and GERD [9]. Most of the previous studies are outdated and statistically underpowered due to small sample sizes. In addition, these observational studies are prone to some inevitable defects such as potential reverse causality and confounding [12]. The causal association between cholelithiasis and GERD remains obscure. Therefore, large datasets and updated methodologies are warranted to

disentangle the conflicting relationship between them and to further reveal the underlying genetic underpinnings.

The evolution of genetic statistical methods has facilitated the understanding of the interconnected genetic basis of complex diseases, providing novel perspectives on the potential biological mechanisms behind the epidemiologic correlations. In our study, we initiated a comprehensive evaluation of the correlations and the shared genetic basis between cholelithiasis and GERD via prospective cohort study, Mendelian randomization (MR) analyses and a range of genetic analyses (**Figure 1**).

Methods

Data summary

Prospective Data from the UK Biobank

UK Biobank (UKB) is a large-scale prospective cohort study with 502,368 participants aged 37–73 years who were recruited between 2006 and 2010 [13]. Participants visited one of 22 assessment centers across England, Scotland, and Wales to complete touch-screen questionnaires, verbal interviews and physical measurements at recruitment.

Data on hospital admissions were collected regularly through linkages to the Scottish Morbidity Records, the Patient Episode Database, and Health Episode Statistics. Information on death was obtained from the National Health Service Central Register and National Health Service Digital. This study was conducted under the UK Biobank project 83339. The UK Biobank received ethical approval from the North West Multi-Centre Research Ethics Committee (21/NW/0157, 16/NW/0274, and 11/NW/0382).

Diagnostic information was sourced from primary care data, hospital admission data and death registry records. We defined diagnoses according to the International Classification of Diseases edition 10 (ICD-10) code: K80 for cholelithiasis and K21 for GERD, respectively.

As shown in the flow chart (**Supplementary Figure**), participants with self-reported cholelithiasis or GERD (N = 13,320) or without follow-up data (N = 1,298) were excluded, leaving 487,750 individuals. To ensure a similar distribution of follow-up time between groups, the index date of participants in the control group were manually assigned based on the distribution of the first diagnosis date of those patients with diseases of interest when conducting corresponding analyses. After excluding 69,862 participants with a history of GERD before the index date, 417,888 participants were finally included to analyze the association between cholelithiasis and GERD. After excluding 62,031 participants with a history of cholelithiasis before the index date, 425,719 participants were finally included to analyze the association between GERD and cholelithiasis.

Follow-up time was calculated from the index date to the time to diagnosis of outcome of interest or the censoring date (October 30th, 2022) or death, whichever occurred first.

Genome-wide association study datasets

Genome-wide association study (GWAS) summary data for cholelithiasis was obtained from the FinnGen databases comprising 32,894 cholelithiasis cases and 301,383 controls of European ancestry [14]. The cholelithiasis dataset was defined with the ICD-10 code K80, ICD-9 code 574 and ICD-8 code 574.

GWAS summary data for GERD was obtained from a meta-analysis of 332,601 individuals including 71,522 cases and 261,079 controls of European ancestry combining the two largest existing genetic studies of GERD (UKB and the QSkin study) [15]. The phenotypes ranged from self-reported GERD, ICD10, and use of GERD medication. For the replication dataset of GERD, we utilized the summary data with 129,080 European ancestry cases and 473,524 European ancestry controls from UK and Australia population [16]. The detailed information of sample collection, quality control, and imputation process for these datasets has been explained in the original articles [14-16]. There is no population overlap between the datasets for cholelithiasis

and GERD. The GWAS summary datasets utilized in this research are publicly available, and the ethical statements can be found in the original publications corresponding to the data. Patients or the public were not involved in the design, or conduct, or reporting, or dissemination plans of our research.

Statistical analysis

Observational analysis

To handle the missing data of the covariates, we applied multiple imputation by chained equations (MICE packages in R) [17] with predictive mean matching method that combining regression models and nearest-neighbor matching. Five imputations and 50 iterations were performed, and one of the five imputations was selected randomly as the final imputed data set.

We constructed a Cox proportional hazards regression model with exposure to cholelithiasis to calculate the hazard ratios (HRs) and 95% CIs. The proportional hazards assumption was tested by Schoenfeld residuals tests, and no evidence of violation was found. Three sets of adjustments were established to minimize the role of confounding. Model 1 was without any adjustments. Model 2 was adjusted only for age and sex. Model 3 was further adjusted for ethnicity, average total annual household income, Deprivation Index, body mass index, alcohol consumption, smoking status, physical activity, education, fresh fruit consumption, raw vegetable consumption, tea consumption, coffee consumption, hypertension, diabetes, renal failure, myocardial infarction, stroke, chronic obstructive pulmonary disease, asthma, anxiety, depression, peptic ulcer. All analyses were performed using RStudio and R 4.2.1 software. Statistical significance was set at a two-tailed P value of less than 0.05.

Mendelian randomization analysis

Two sample MR analysis, using genetic variants as instruments, were performed with R packages "*TwoSampleMR*" [18], and "*MR-PRESSO*" [19] in R software (version

4.2.1). Ensuring the validity of causal inference relies on three critical assumptions of independence, relevance, and exclusion restriction [20]. These assumptions are indispensable for mitigating bias and establishing causality. Only significant single nucleotide polymorphisms (SNPs) independently associated with the exposure at a *P*-threshold of 5×10^{-8} and satisfying the linkage disequilibrium (LD) criteria: $r^2 < 0.001$ and kb > 10,000 were identified as instruments in MR studies.

We employed inverse variance weighting (IVW) [21] as the main MR approach, complemented by three additional sensitivity analysis methods, including MR-Egger [22], weighted median [23] and weighted mode [24], to detect the causal relationships between cholelithiasis and GERD. Different methods were based on different assumptions concerning the influence of horizontal pleiotropy. The IVW MR model, assuming balanced pleiotropy, applies multiplicative random effects to meta-analyze the Wald estimates of each SNP [21]. The MR-Egger model allows the uncorrelated directional pleiotropy by adding a nonzero intercept which relaxes the assumption of relevance of selected genetic variants [22]. The weighted median and weighted mode models remain robust when up to 50% or more of genetic variants are valid, which exhibit greater resilience to pleiotropy [24, 25].

We conducted MR-Egger intercept test, Cochran's Q statistic, MR-PRESSO and leave-one-out analysis to evaluate the heterogeneity, pleiotropy, and potential outliers of the MR results. If heterogeneity is detected in the MR analysis ($P < 0.05$), we would recalculate the MR estimates after the removal of outliers identified with a *P* value of less than 1 in the MR-PRESSO outlier test to ensure the robustness of the MR results. The MR analysis in this research has been documented in accordance with the Strengthening the Reporting of Observational Studies in Epidemiology (STROBE) guideline specific for MR study.

Global genetic correlation analysis

To quantify the heritability of each trait and the global genetic correlation between cholelithiasis and GERD, we applied linkage disequilibrium score regression (LDSC)

method with Python 2.7 [26]. Based on pre-computed LD scores derived from 1000 Genomes reference data of European population, we selected SNPs that matched the reference panel (minor allele frequency (MAF) > 0.01 and INFO score > 0.9) in the GWAS datasets [27]. We used univariate LDSC to estimate SNP heritability for each trait and bivariate LDSC to calculate the genetic correlations between cholelithiasis and GERD with and without constraining the intercept. The genetic correlation with *P*-value less than 0.05 was considered significant [28, 29].

Additionally, we employed the genetic covariance analyzer (GNOVA) as a supplementary method to validate the genetic correlations. The steps of quality control on GWAS datasets are similar with the LDSC method [30]. More detailed descriptions are in the original study [31]. Based on the framework of the annotation-stratified genetic covariance estimation, GNOVA provides more powerful statistical inference of the shared genetic basis between complex traits and shows higher estimation accuracy. Threshold of $P < 0.05$ was regarded as strong evidence for MAF-stratified genetic correlation [31].

Local genetic correlation analysis

To identify whether cholelithiasis and GERD have genetic correlation in local genomic region, we further applied Heritability Estimator from Summary Statistics (ρ-HESS) with Python 2.7 [32]. We firstly calculated the LD block and eigenvalues by referring to the 1000 Genomes Project of Europeans. Then, we explored the local SNP heritability for each trait and estimated the local genetic correlation in 1,613 potential LD-independent regions [33]. Suggestive genetic associations with *P* value less than 0.05 was noted.

Similarly, Pairwise-GWAS (GWAS-PW) was supplemented to explore the significant shared local regions [34]. Based on the Bayesian statistical framework, GWAS-PW calculated the posterior probabilities of association (PPA) for each genomic region across 4 models. Genomic regions with PPA of model 3 larger than 0.5 were

considered to be significantly associated with both traits, in accordance with a previous article [35, 36].

Cross-trait meta-analysis

To detect the shared genetic variants in cholelithiasis and GERD, we performed multi-trait analysis of GWAS (MTAG) [37]. MTAG is based on a fundamental assumption that all SNPs exhibit the same variance-covariance matrix of effect sizes and heritability across traits. To meet the assumption, we rigorously filtered the MTAG SNP with MAF $\geq 1\%$ and sample size $\geq 75\%$ of the 90th percentile and dropped the outliers [37]. By joint analysis of multiple traits, MTAG substantially enhances the statistical power to detect the genetic associations for each trait and generate trait-specific estimates for each SNP. To identify the significant and independent loci, we utilized the threshold $P_{\mathrm{MTAG}} < 5 \times 10^{-8}$ and the "clumping" function of PLINK (settings: clump_p1 = $5e^{-8}$, clump_p2 = $1e^{-5}$, clump_r^2 = 0.2, clump_kb = 500) [38].

Cross-phenotype association test (CPASSOC) is a complementary method to deduce the shared risk SNPs between complex traits [39]. Compared with the single trait analysis, CPASSOC improves statistical power and reasonably controlled type I error rate. Considering the heterogeneity effects for different phenotypes, we primarily used the heterogonous version of cross-phenotype statistic (Shet) method to integrate association evidence of different but correlated traits [40]. Given the inherent variability induced by the random sampling analysis embedded in this method, we set a random seed to 123 to ensure a reproducible result. After getting the estimates, we identified the independent loci using the "clumping" function of PLINK (settings as before). The variant in each locus with the smallest *P*-value was regarded as the index SNP. Index SNPs that met the criteria of $P_{\mathrm{CPASSOC}} < 5 \times 10^{-8}$ and $P_{\mathrm{each\ trait}} < 1 \times 10^{-3}$ were deemed significant pleiotropic SNPs. Newly discovered pleiotropic SNPs were defined as those significant pleiotropic SNPs which were not genome-wide significant ($5 \times 10^{-8} < P_{\mathrm{each\ trait}} < 1 \times 10^{-3}$), were independent ($r^2 < 0.20$) of earlier identified trait-related genome-

wide significant SNPs, and all their adjacent SNPs (± 500 kb) didn't reach $P < 5 \times 10^{-8}$ in each GWAS dataset.

We used dbSNP (https://www.ncbi.nlm.nih.gov/snp/) and 3DSNP (https://omic.tech/3dsnpv2/) for detailed functional annotation of the identified pleiotropic SNPs.

Transcriptome-wide association analysis

Numerous genetic variants impact intricate traits through the regulation of gene expression. To identify significant gene-trait associations, we implemented a transcriptome wide association scan (TWAS) leveraging FUSION software [41]. Based on the LD reference data of European 1000 Genome, we converted the GWASs of cholelithiasis and GERD into LD-score format. We prioritized the trait-related tissues; thus, we prepared the expression quantitative traits loci (eQTL) data of whole blood, liver, stomach, and esophagus-related tissues from GTEx v8 (Genotype-Tissue Expression, version 8, http://gusevlab.org/projects/fusion/). By integrating the precomputed phenotypic summary data and corresponding eQTL data, we identified significant tissue-specific genes with false discovery rate (FDR) < 0.05 for each trait and selected genes that overlapped between cholelithiasis and GERD in the same tissue.

Summary data-based Mendelian Randomization (SMR) analysis is a complementary method to deduce the causative genes underlying cholelithiasis and GERD [42]. We used the eQTL data of whole blood, liver, stomach, and esophagus-related tissues from GTEx v8 [43] and cis-eQTL data of whole blood from eQTLGen consortium [44]. The heterogeneity in dependent instruments (HEIDI) test was conducted to distinguish pleiotropy or causality from linkage. We primarily focused on the genes with FDR < 0.05 and passed the *P*-value thresholds for HEIDI test ($P_{\text{HEIDI}} > 0.05$) [42].

Results

Observational association between cholelithiasis and GERD

Baseline characteristics of the study cohort by cholelithiasis are presented in **Supplementary Table 1**. In total, participants were followed for 2,736,451 person-years, during which 1,628 cholelithiasis patients and 20,780 non-cholelithiasis individuals developed GERD (**Table 1A**). In the age/sex-adjusted model, the risk of GERD was 2.28 times higher in cholelithiasis patients compared to those without cholelithiasis. In the fully-adjusted model, the risk of GERD remained statistically significant in cholelithiasis patients (HR = 1.99, 95% CI = 1.89 - 2.10, $P < 0.001$).

Moreover, we also observed the association between baseline GERD and incident cholelithiasis as shown in **Table 1B**. In the age/sex-adjusted model, the HR for cholelithiasis was 2.69 (95% CI = 2.54-2.84, $P < 0.001$) for GERD patients. In the fully adjusted model, the GERD group also displayed a significantly increased risk of developing cholelithiasis (HR = 2.30, 95% CI = 2.18-2.44, $P < 0.001$).

Causal association between cholelithiasis and GERD

Using forty-six cholelithiasis-associated and twenty-one GERD-associated genetic instruments (**Supplementary Table 2**), respectively, our study provided evidence for the causal association between cholelithiasis and GERD. Genetically determined cholelithiasis has the possibility to increase the risk of GERD by 7% (IVW OR = 1.07, 95%CI = 1.05-1.10, $P = 3.16 \times 10^{-9}$, **Figure 2A, Supplementary Table 3**), which was further validated by other three MR methods and the analyses with a supplementary dataset (**Supplementary Table 4**). Besides, genetically predicted GERD could increase the risk of cholelithiasis by 15% (OR = 1.15, 95%CI = 1.02-1.30, $P = 0.025$) according to IVW method (**Figure 2B, Supplementary Table 3**). This association was further substantiated through analyses using an additional dataset (**Supplementary Table 4**).

The F statistic of each SNP related to cholelithiasis and GERD was found to be larger than the empirical threshold of 10, suggesting little possibility of weak instrument

bias (**Supplementary Table 2**). We also performed several sensitive analyses to validate the causal association between cholelithiasis and GERD. The Cochran's Q test in the IVW model and MR Egger model suggested a lack of evidence for the existence of heterogeneity in effects across the instrumental variables. *P* value of the MR Egger intercept test was larger than 0.05, which indicated that there was lower possibility of horizontal pleiotropy in the causal estimates (**Supplementary Table 3-4**).

Global and local genetic correlations between cholelithiasis and GERD

SNP-based liability-scale heritability h^2 for cholelithiasis and GERD were 7.21% and 7.61% when utilizing the univariate LDSC with constraining the intercept, as well as 6.59% and 7.68% utilizing GNOVA. The cross-trait LDSC suggested that cholelithiasis had a relatively strong positive genetic correlation with GERD, exhibiting a genetic correlation (r_g) of 0.31 and a *P* value of 2.77×10^{-27}. After constraining the intercept, the genetic correlation was decreased but remained significant ($r_g = 0.25$, $P = 3.90 \times 10^{-56}$). This finding was consistent with the GNOVA analysis, reflecting a genetic correlation (r_g) of 0.26 and a *P* value of 2.50×10^{-32} (**Table 2**).

We also tested the local genetic correlation by ρ-HESS and GWAS-PW (**Supplementary Table 5**). Seven suggestively significant regions were identified by ρ-HESS and eight significant regions were identified by GWAS-PW. Four regions were overlapped according to ρ-HESS and GWAS-PW. These findings suggested a potential shared genetic foundation, necessitating further exploration to elucidate the underlying biological mechanisms.

Identification of shared risk loci for cholelithiasis and GERD

MTAG identified eight independent pleiotropic loci (rs146812426, rs4299376, rs6733452, rs7596134, rs4681515, rs9297994, rs10935762, rs3922717), which were also significant in CPASSOC (**Table 3, Supplementary Table 6**). CPASSOC found twenty-three pleiotropic loci, five of which were found significant in MTAG including rs9297994, rs10935762, rs3922717, rs12633863 and rs802036 (**Table 3, Supplementary Table 7**). Overall, ten independently significant loci have been

identified as shared between cholelithiasis and GERD by both MTAG and CPASSOC, namely rs146812426, rs4299376, rs6733452, rs7596134, rs10935762, rs12633863, rs4681515, rs3922717, rs802036, rs9297994, which mapped to nine genes including *PLEKHH2, ABCG8, DYNC2LI1, ABCG5, TM4SF4, LOC100270746, CROT, UBXN2B, CYP7A1* (**Table 3**). It is worth noting that five novel pleiotropic loci were identified in CPASSOC analysis, including rs10167227, rs6742945, rs335208, rs72664027 and rs11537754, which mapped to genes *PNPT1*, *LOC105369165*, *PRDM6*, *LINC02842*, and *RAB11FIP3*, respectively (**Table 3, Supplementary Table 7**). Other SNPs-associated genes are listed in **Supplementary Table 6-7**.

Identification of shared genes for cholelithiasis and GERD

Results from tissue-specific TWAS and SMR revealed gene-level genetic overlap. After FDR corrections, a total of fifteen genes were shared by cholelithiasis and GERD, enriched in six tissues including blood, liver, esophagus mucosa, esophagus muscularis, esophagus gastroesophageal junction, stomach in TWAS analysis (**Supplementary Table 8**). Among them, seven genes significantly overlapped in two or more tissues. Five of seven genes (*SUN2*, *CBY1*, *JOSD1*, *DDX17*, *FAM227A*) were located in 22q13.1. The TWAS analysis showed that over expression of *SUN2*, *JOSD1* and *CBY1* were negatively associated with the risk of cholelithiasis and GERD in the blood and esophagus-related tissues, while over expression of *JOSD1* and *CBY1* were positively associated with these two diseases in the liver tissue. *SUN2*, *JOSD1* and *CBY1* also displayed a significant SMR association signal with $FDR < 0.05$ and passed the HEIDI-outlier test in blood, esophagus mucosa, and esophagus muscularis (**Supplementary Table 9**). No significant shared causal gene was found in other tissues namely liver, esophagus gastroesophageal junction, and stomach according to SMR results.

Discussion

To our knowledge, this is the first study to comprehensively explore the observational, causal and genetic relationships between cholelithiasis and GERD. By leveraging UK

Biobank data and GWAS data, we found the bidirectional causal relationship between cholelithiasis and GERD. The subsequent genetic analyses provided new insights into their shared genetic basis and related biological mechanism, which may contribute to the prediction, diagnosis, and treatment of these diseases.

Previous research has reported that cholelithiasis and GERD shared numerous common etiological risk factors such as obesity [45], type 2 diabetes mellitus [46], depression [47], and smoking [48]. We conducted a Cox proportional hazards regression model analysis using the UKB cohort, with adjustments for a wide range of established and potential confounders associated with these two conditions. Although the HRs were slightly attenuated after controlling the covariates, the bidirectional association between cholelithiasis and GERD remained statistical significance. This is consistent with the findings by Unalp-Arida A et al. and Portincasa et al., which reported a statistically significant association between cholelithiasis and GERD [8, 11]. Subsequently, using the MR approach, we identified bidirectional causality between cholelithiasis and GERD, while the pathophysiologic mechanisms underlying the causal relationship remains unclear. Previous studies suggested that patients with gallstones showed impaired gastric motility [4, 8], which might be related to the pathogenesis of GERD. Meanwhile, patients with GERD presented a higher incidence of gallbladder dyskinesia [49, 50], which may be attributed to the routine use of proton pump inhibitors (PPIs) in GERD treatment. It has been reported that PPIs could reduce the release of cholecystokinin, which might diminish gallbladder motility, thereby causing the formation of gallstones [51]. The current evidence indicated the potential shared pathogenesis or genetic basis between cholelithiasis and GERD, warranting further exploration.

Findings from LDSC and GNOVA revealed a significant genetic correlation between cholelithiasis and GERD, which supported the hypothesis that genetic factors play an important role in the co-occurrence of cholelithiasis and GERD. Subsequently, we identified four regions that exhibited a suggestively significant local genetic association, as evidenced by ρ-HESS < 0.05 and GWAS-PW > 0.5. The majority of loci

identified by MTAG and CPASSOC were situated within these regions. Moreover, we found that 22q13.1 might be a shared region between gallstone disease and GERD by combing analyses of local genetic correlation, shared loci and shared genes: First, this region showed suggestively significant local genetic association between cholelithiasis and GERD using GWAS-PW. Second, the shared loci rs1056661, identified by CPASSOC, was located within this region. Third, five and three overlapped genes, identified from TWAS analysis and SMR respectively, were situated within this region. Previous studies have reported that several significant loci related to gallstone disease including rs12004, rs41281265, rs1946990, were in this region [52, 53]. However, currently, there is no research linking this region to GERD. Future research is warranted to delve deeper into this specific region to elucidate the genetic correlation between gallstone disease and GERD.

Given the significant genetic correlation observed, we conducted cross-trait GWAS meta-analyses to detect risk SNPs underlying the joint phenotypes cholelithiasis-GERD. We identified 10 shared independently significant loci through MTAG and CPASSOC. Among the genes related to these loci, *ABCG5* (index SNP: rs7596134), *ABCG8* (index SNP: rs4299376 and rs6733452) and *CYP7A1* (index SNP: rs9297994) are associated with lipid metabolism. Numerous investigations have suggested the involvement of these genes in the development of gallstone disease [54-56]. Although several studies have reported that obesity [45] and dyslipidemia [57, 58] are risk factors for GERD, no research has investigated these genes in relation to GERD. Therefore, the relationship between these genes and GERD warrants further investigation. Additionally, five new loci associated with cholelithiasis and GERD was identified via CPASSOC analysis. *PNPT1* (index SNP: rs10167227) is associated with the mitochondrial respiratory chain, and mutations in *PNPT1* can lead to mitochondrial dysfunction, subsequently causing neuromuscular dysfunction which affects the peristaltic function of the gastrointestinal tract [59, 60]. The functions of lncRNA gene *LINC02842* (index SNP: rs72664027) and ncRNA gene *LOC105369165* (index SNP: rs6742945) remain unclear but a research has suggested that lncRNAs might have a crucial role in the dysfunction of lower

esophageal sphincter (LES) [61], potentially shedding light on the onset of GERD. Additional research is required to offer more detailed functional annotation of these shared loci.

In addition to detecting shared loci, we also explored whether the cholelithiasis-GERD association can be mediated by shared risk genes through TWAS and SMR analysis. In general, we identified three putatively functional genes shared between cholelithiasis and GERD, including *SUN2*, *CBY1* and *JOSD1*, over expression of which were negatively associated with the risk of cholelithiasis and GERD in the esophagus-related tissues. Prior research has reported the negative effect of *CBY1* and *SUN2* genes on tumorigenesis [62-64], which implied a potential role of them in the pathogenesis of gallstone disease and GERD, given that these two diseases are risk factors for gallbladder and esophageal cancer, respectively [65, 66]. Furthermore, existing studies suggested the involvement of bile acids in GERD progression through the activation of the Wnt/Wingless pathway [67]. *CBY1* may be involved in the linkage between gallstone disease and GERD as it can inhibit the Wnt/Wingless pathway [68]. *JOSD1* is a deubiquitinating enzyme, playing a pivotal role in many cellular biological processes [69]. Our findings imply that *JOSD1* may play a significant role in the associative mechanisms between cholelithiasis and GERD via the deubiquitination processes. In general, our study offers novel insights into the underlying shared genetic basis of cholelithiasis and GERD, and additional research is required for a more profound elucidation.

Strengths and Limitations

In our study, we conducted the largest prospective study assessing the phenotypic association between cholelithiasis and incident GERD. Besides, we performed a series of sensitive analyses and further applied validation datasets in MR estimates to enhance the robustness of our results. Furthermore, genetic correlation, pleiotropic loci and gene detection were fully analyzed by two different approaches. The convergent evidence acquired through these dual approaches reinforces the reliability of our findings.

However, several limitations need to be acknowledged. First, although we adjusted for as many confounders as possible in our observational study, the potential influence of unknown confounders on our results cannot be entirely ruled out. Nevertheless, we performed the MR analysis to deal with this bias and built the causality of cholelithiasis and GERD. Second, all the data used in this study came from European ancestry populations, which limited the extension of our findings to other ethnic populations; thus, future studies involving a broader range of ancestries are warranted. Third, due to limited GWAS data availability at the time of conducting the analysis, we were unable to perform a deeper subgroup analysis based on the stratification information, such as age, gender, severity of the disease, etc.

Conclusion

In sum, our study is the first to establish the bidirectional causal association between cholelithiasis and GERD and reveal the shared genetic basis based on the evidence of the significant genetic correlation, novel shared loci and genes. Our findings provided new insights into the biological mechanisms for cholelithiasis and GERD and suggested promising therapeutic targets, which might provide an innovative research direction for future therapeutic strategy and risk prediction.

Acknowledgements

We sincerely appreciate the UK Biobank for the access of individual data, and this research has been performed under approval (Application Number 83339). We also thank the FinnGen and UK Biobank for providing access to the public GWAS summary statistics.

Funding

This work was funded by the National Natural Science Foundation of China Regional Innovation and Development Joint Foundation (U23A20408), National Natural Science Foundation of China (82171698, 82170561, 81300279, 81741067), the Natural Science Foundation for Distinguished Young Scholars of Guangdong Province (2021B1515020003), Project to Attract Foreign Experts from Minister of Science and Technology of China (G2022030047L), Natural Science Foundation of Guangdong Province (2022A1515012081), the Foreign Distinguished Teacher Program of Guangdong Science and Technology Department (KD0120220129), the Climbing Program of Introduced Talents and High-level Hospital Construction Project of Guangdong Provincial People's Hospital (DFJH201923, DFJH201803, KJ012019099, KJ012021143, KY012021183), and in part by VA Clinical Merit and ASGE clinical research funds (FWL).

Ethical approval statement

UK Biobank has received ethical approval from the UK National Health Service's National Research Ethics Service ((21/NW/0157, 16/NW/0274, and 11/NW/0382), and this research has been performed under the UK Biobank approved project (Application Number 83339). The ethical approval of GWAS statistics had been obtained in the original studies. All data generated during this study are included in this article and supplementary materials.

Declaration of competing interests

The authors declare that they have no competing interests.

Author Contributions

Conceptualization and design: Felix W Leung, Qizhou Lian, Weihong Sha, Hao Chen; Collection and assembly of data: Yanlin Lyu, Shuangshuang Tong, Yuying Ma; Data analysis and interpretation: Yanlin Lyu, Shuangshuang Tong, Wentao Huang, Yuying Ma; Manuscript writing-original draft: Yanlin Lyu, Shuangshuang Tong; Manuscript writing-review & editing: Yanlin Lyu, Shuangshuang Tong, Ruijie Zeng, Rui Jiang, Wentao Huang, Ruibang Luo, Felix W Leung, Qizhou Lian, Weihong Sha, Hao Chen. All authors reviewed and approved the final manuscript. All authors had full access to all the data in the study and had final responsibility for the decision to submit for publication. All authors were not precluded from accessing data in the study, and they accept responsibility to submit for publication.

Data availability statement

Primary data from the UK Biobank resource are accessible upon application (https://www.ukbiobank.ac.uk/). Dataset of cholelithiasis was downloaded at https://www.finngen.fi/en/access_results and datasets of gastroesophageal reflux disease were downloaded at 10.6084/m9.figshare.8986589 and https://cnsgenomics.com/content/data. The analysis code used in this study is available from the corresponding authors upon reasonable request.

Patient and Public Involvement

Patients or the public WERE NOT involved in the design, or conduct, or reporting, or dissemination plans of our research.

References

1. Lammert F, Gurusamy K, Ko CW, Miquel J-F, Méndez-Sánchez N, Portincasa P, van Erpecum KJ, van Laarhoven CJ, Wang DQH: Gallstones. *Nat Rev Dis Primers* 2016, **2**:16024.
2. Wang F, Wang J, Li Y, Yuan J, Yao P, Wei S, Guo H, Zhang X, Yang H, Wu T *et al*: Gallstone Disease and Type 2 Diabetes Risk: A Mendelian Randomization Study. *Hepatology* 2019, **70**(2):610-620.

3. Katsika D, Grjibovski A, Einarsson C, Lammert F, Lichtenstein P, Marschall H-U: Genetic and environmental influences on symptomatic gallstone disease: a Swedish study of 43,141 twin pairs. *Hepatology* 2005, **41**(5):1138-1143.
4. Di Ciaula A, Molina-Molina E, Bonfrate L, Wang DQH, Dumitrascu DL, Portincasa P: Gastrointestinal defects in gallstone and cholecystectomized patients. *Eur J Clin Invest* 2019, **49**(3):e13066.
5. Richter JE, Rubenstein JH: Presentation and Epidemiology of Gastroesophageal Reflux Disease. *Gastroenterology* 2018, **154**(2):267-276.
6. Maret-Ouda J, Markar SR, Lagergren J: Gastroesophageal Reflux Disease. *JAMA* 2020, **324**(24):2565.
7. Katzka DA, Kahrilas PJ: Advances in the diagnosis and management of gastroesophageal reflux disease. *BMJ* 2020, **371**:m3786.
8. Portincasa P, Di Ciaula A, Palmieri V, Velardi A, VanBerge-Henegouwen GP, Palasciano G: Impaired gallbladder and gastric motility and pathological gastro-oesophageal reflux in gallstone patients. *Eur J Clin Invest* 1997, **27**(8):653-661.
9. Avidan B, Sonnenberg A, Schnell TG, Sontag SJ: No association between gallstones and gastroesophageal reflux disease. *Am J Gastroenterol* 2001, **96**(10):2858-2862.
10. Räihä I, Impivaara O, Seppälä M, Knuts LR, Sourander L: Determinants of symptoms suggestive of gastroesophageal reflux disease in the elderly. *Scand J Gastroenterol* 1993, **28**(11):1011-1014.
11. Unalp-Arida A, Der JS, Ruhl CE: Longitudinal Study of Comorbidities and Clinical Outcomes in Persons with Gallstone Disease Using Electronic Health Records. *J Gastrointest Surg* 2023.
12. Zhu Z, Hasegawa K, Camargo CA, Liang L: Investigating asthma heterogeneity through shared and distinct genetics: Insights from genome-wide cross-trait analysis. *J Allergy Clin Immunol* 2021, **147**(3):796-807.
13. Sudlow C, Gallacher J, Allen N, Beral V, Burton P, Danesh J, Downey P, Elliott P, Green J, Landray M *et al*: UK biobank: an open access resource for identifying the causes of a wide range of complex diseases of middle and old age. *PLoS Med* 2015, **12**(3):e1001779.
14. Kurki MI, Karjalainen J, Palta P, Sipilä TP, Kristiansson K, Donner KM, Reeve MP, Laivuori H, Aavikko M, Kaunisto MA *et al*: FinnGen provides genetic insights from a well-phenotyped isolated population. *Nature* 2023, **613**(7944):508-518.
15. An J, Gharahkhani P, Law MH, Ong J-S, Han X, Olsen CM, Neale RE, Lai J, Vaughan TL, Gockel I *et al*: Gastroesophageal reflux GWAS identifies risk loci that also associate with subsequent severe esophageal diseases. *Nat Commun* 2019, **10**(1):4219.
16. Ong JA-OX, An J, Han X, Law MH, Nandakumar P, Schumacher J, Gockel I, Bohmer A, Jankowski J, Palles C *et al*: Multitrait genetic association analysis identifies 50 new risk loci for gastro-oesophageal reflux, seven new loci for Barrett's oesophagus and provides insights into clinical

heterogeneity in reflux diagnosis. (1468-3288 (Electronic)).

17. Jolani S, Debray TPA, Koffijberg H, van Buuren S, Moons KGM: **Imputation of systematically missing predictors in an individual participant data meta-analysis: a generalized approach using MICE.** *Stat Med* 2015, **34**(11):1841-1863.

18. Hemani G, Zheng J, Elsworth B, Wade KH, Haberland V, Baird D, Laurin C, Burgess S, Bowden J, Langdon R *et al*: **The MR-Base platform supports systematic causal inference across the human phenome.** *eLife* 2018, **7**.

19. Verbanck M, Chen C-Y, Neale B, Do R: **Detection of widespread horizontal pleiotropy in causal relationships inferred from Mendelian randomization between complex traits and disea ses.** *Nature genetics*, **50**(5):693-698.

20. Davies NM, Holmes MV, Davey Smith G: **Reading Mendelian randomisation studies: a guide, glossary, and checklist for clinicians.** *BMJ* 2018, **362**:k601.

21. Burgess S, Butterworth A, Thompson SG: **Mendelian randomization analysis with multiple genetic variants using summarized data.** *Genetic epidemiology* 2013, **37**(7):658-665.

22. Burgess S, Thompson SG: **Interpreting findings from Mendelian randomization using the MR-Egger method.** *Eur J Epidemiol* 2017, **32**(5):377-389.

23. Bowden J, Davey Smith G, Haycock PC, Burgess S: **Consistent Estimation in Mendelian Randomization with Some Invalid Instruments Using a Weighted Median Estimator.** *Genetic epidemiology* 2016, **40**(4):304-314.

24. Hartwig FP, Davey Smith G, Bowden J: **Robust inference in summary data Mendelian randomization via the zero modal pleiotropy assumption.** *International journal of epidemiology* 2017, **46**(6):1985-1998.

25. Bowden J, Davey Smith G, Haycock PC, Burgess S: **Consistent Estimation in Mendelian Randomization with Some Invalid Instruments Using a Weighted Median Estimator.** *Genet Epidemiol* 2016, **40**(4):304-314.

26. Finucane HK, Bulik-Sullivan B, Gusev A, Trynka G, Reshef Y, Loh P-R, Anttila V, Xu H, Zang C, Farh K *et al*: **Partitioning heritability by functional annotation using genome-wide association summary statistics.** *Nature genetics* 2015, **47**(11):1228-1235.

27. Auton A, Brooks LD, Durbin RM, Garrison EP, Kang HM, Korbel JO, Marchini JL, McCarthy S, McVean GA, Abecasis GR: **A global reference for human genetic variation.** *Nature* 2015, **526**(7571):68-74.

28. Bulik-Sullivan B, Finucane HK, Anttila V, Gusev A, Day FR, Loh P-R, Duncan L, Perry JRB, Patterson N, Robinson EB *et al*: **An atlas of genetic correlations across human diseases and traits.** *Nature genetics* 2015, **47**(11):1236-1241.

29. Yao Y, Li Ce, Meng P, Cheng B, Cheng S, Liu L, Yang X, Jia Y, Wen Y, Zhang F: **An atlas of genetic correlations between gestational age and common psychiatric disorders.** *Autism Res* 2022, **15**(6):1008-1017.

30. Perry BI, Bowker N, Burgess S, Wareham NJ, Upthegrove R, Jones PB, Langenberg C, Khandaker GM: **Evidence for Shared Genetic Aetiology Between**

Schizophrenia, Cardiometabolic, and Inflammation-Related Traits: Genetic Correlation and Colocalization Analyses. *Schizophr Bull Open* 2022, **3**(1):sgac001.

31. Lu Q, Li B, Ou D, Erlendsdottir M, Powles RL, Jiang T, Hu Y, Chang D, Jin C, Dai W *et al*: **A Powerful Approach to Estimating Annotation-Stratified Genetic Covariance via GWAS Summary Statistics**. *Am J Hum Genet* 2017, **101**(6):939-964.
32. Shi H, Mancuso N, Spendlove S, Pasaniuc B: **Local Genetic Correlation Gives Insights into the Shared Genetic Architecture of Complex Traits**. *Am J Hum Genet* 2017, **101**(5):737-751.
33. Berisa T, Pickrell JK: **Approximately independent linkage disequilibrium blocks in human populations**. *Bioinformatics* 2016, **32**(2):283-285.
34. Pickrell JK, Berisa T, Liu JZ, Ségurel L, Tung JY, Hinds DA: **Detection and interpretation of shared genetic influences on 42 human traits**. *Nature genetics* 2016, **48**(7):709-717.
35. Mortlock S, Corona RI, Kho PF, Pharoah P, Seo J-H, Freedman ML, Gayther SA, Siedhoff MT, Rogers PAW, Leuchter R *et al*: **A multi-level investigation of the genetic relationship between endome triosis and ovarian cancer histotypes**. *Cell Rep Med*, **3**(3):100542.
36. Wu X, Zhang W, Zhao X, Zhang L, Xu M, Hao Y, Xiao J, Zhang B, Li J, Kraft P *et al*: **Investigating the relationship between depression and breast cancer: observational and genetic analyses**. *BMC Med* 2023, **21**(1):170.
37. Turley P, Walters RK, Maghzian O, Okbay A, Lee JJ, Fontana MA, Nguyen-Viet TA, Wedow R, Zacher M, Furlotte NA *et al*: **Multi-trait analysis of genome-wide association summary statistics using MTAG**. *Nature genetics* 2018, **50**(2):229-237.
38. Purcell S, Neale B, Todd-Brown K, Thomas L, Ferreira MAR, Bender D, Maller J, Sklar P, de Bakker PIW, Daly MJ *et al*: **PLINK: a tool set for whole-genome association and population-based linkage analyses**. *Am J Hum Genet* 2007, **81**(3):559-575.
39. Zhu X, Feng T, Tayo BO, Liang J, Young JH, Franceschini N, Smith JA, Yanek LR, Sun YV, Edwards TL *et al*: **Meta-analysis of correlated traits via summary statistics from GWASs with an application in hypertension**. *Am J Hum Genet* 2015, **96**(1):21-36.
40. Li X, Zhu X: **Cross-Phenotype Association Analysis Using Summary Statistics from GWAS**. *Methods Mol Biol* 2017, **1666**:455-467.
41. Gusev A, Ko A, Shi H, Bhatia G, Chung W, Penninx BWJH, Jansen R, de Geus EJC, Boomsma DI, Wright FA *et al*: **Integrative approaches for large-scale transcriptome-wide association studies**. *Nature genetics* 2016, **48**(3):245-252.
42. Zhu Z, Zhang F, Hu H, Bakshi A, Robinson MR, Powell JE, Montgomery GW, Goddard ME, Wray NR, Visscher PM *et al*: **Integration of summary data from GWAS and eQTL studies predicts complex trait gene targets**. *Nature genetics* 2016, **48**(5):481-487.

43. Battle A, Brown CD, Engelhardt BE, Montgomery SB: **Genetic effects on gene expression across human tissues.** *Nature* 2017, **550**(7675):204-213.

44. Võsa U, Claringbould A, Westra H-J, Bonder MJ, Deelen P, Zeng B, Kirsten H, Saha A, Kreuzhuber R, Yazar S *et al*: **Large-scale cis- and trans-eQTL analyses identify thousands of genetic loci and polygenic scores that regulate blood gene expression.** *Nature genetics* 2021, **53**(9):1300-1310.

45. Yuan S, Ruan X, Sun Y, Fu T, Zhao J, Deng M, Chen J, Li X, Larsson SC: **Birth weight, childhood obesity, adulthood obesity and body composition, and gastrointestinal diseases: a Mendelian randomization study.** *Obesity (Silver Spring)* 2023, **31**(10):2603-2614.

46. Chen J, Yuan S, Fu T, Ruan X, Qiao J, Wang X, Li X, Gill D, Burgess S, Giovannucci EL *et al*: **Gastrointestinal Consequences of Type 2 Diabetes Mellitus and Impaired Glycemic Homeostasis: A Mendelian Randomization Study.** *Diabetes Care* 2023, **46**(4):828-835.

47. Ruan X, Chen J, Sun Y, Zhang Y, Zhao J, Wang X, Li X, Yuan S, Larsson SC: **Depression and 24 gastrointestinal diseases: a Mendelian randomization study.** *Transl Psychiatry* 2023, **13**(1):146.

48. Yuan S, Chen J, Ruan X, Sun Y, Zhang K, Wang X, Li X, Gill D, Burgess S, Giovannucci E *et al*: **Smoking, alcohol consumption, and 24 gastrointestinal diseases: Mendelian randomization analysis.** *eLife* 2023, **12**.

49. Li Y, Duan Z: **Updates in interaction of gastroesophageal reflux disease and extragastroesophageal digestive diseases.** (1747-4132 (Electronic)).

50. Izbéki F, Rosztóczy Ai Fau - Yobuta JS, Yobuta Js Fau - Róka R, Róka R Fau - Lonovics J, Lonovics J Fau - Wittmann T, Wittmann T: **Increased prevalence of gallstone disease and impaired gallbladder motility in patients with Barrett's esophagus.** (0163-2116 (Print)).

51. Cahan MA, Balduf L Fau - Colton K, Colton K Fau - Palacioz B, Palacioz B Fau - McCartney W, McCartney W Fau - Farrell TM, Farrell TM: **Proton pump inhibitors reduce gallbladder function.** (1432-2218 (Electronic)).

52. Ferkingstad E, Oddsson A, Gretarsdottir S, Benonisdottir S, Thorleifsson G, Deaton AM, Jonsson S, Stefansson OA, Norddahl GL, Zink F *et al*: **Genome-wide association meta-analysis yields 20 loci associated with gallstone disease.** *Nat Commun* 2018, **9**(1):5101.

53. Fairfield CJ, Drake TM, Pius R, Bretherick AD, Campbell A, Clark DW, Fallowfield JA, Hayward C, Henderson NC, Iakovliev A *et al*: **Genome-wide analysis identifies gallstone-susceptibility loci including genes regulating gastrointestinal motility.** *Hepatology* 2022, **75**(5):1081-1094.

54. Kuo KK, Shin SJ, Chen ZC, Yang YHC, Yang JF, Hsiao PJ: **Significant association of ABCG5 604Q and ABCG8 D19H polymorphisms with gallstone disease.** *Br J Surg* 2008, **95**(8):1005-1011.

55. Jiang Z-Y, Han T-Q, Suo G-J, Feng D-X, Chen S, Cai X-X, Jiang Z-H, Shang J, Zhang Y, Jiang Y *et al*: **Polymorphisms at cholesterol 7alpha-hydroxylase, apolipoproteins B and E and low density lipoprotein receptor genes in patients with gallbladder stone disease.** *World J Gastroenterol* 2004,

10(10):1508-1512.

56. Qayyum F, Lauridsen BK, Frikke-Schmidt R, Kofoed KF, Nordestgaard BG, Tybjærg-Hansen A: **Genetic variants in CYP7A1 and risk of myocardial infarction and symptomatic gallstone disease**. *Eur Heart J* 2018, **39**(22):2106-2116.

57. Fujikawa Y, Tominaga K, Fujii H, Machida H, Okazaki H, Yamagami H, Tanigawa T, Watanabe K, Watanabe T, Fujiwara Y *et al*: **High prevalence of gastroesophageal reflux symptoms in patients with non-alcoholic fatty liver disease associated with serum levels of triglyceride and cholesterol but not simple visceral obesity**. *Digestion* 2012, **86**(3):228-237.

58. Asekritova AS, Kylbanova ES, Emelyanova EA, Borisova EP: **GASTROESOPHAGEAL REFLUX DISEASE IN ASSOCIATION WITH LIPID-METABOLIC INDICATORS AT THE YAKUTSK**. *Wiad Lek* 2015, **68**(4):449-453.

59. Hom XB, Lavine JE: **Gastrointestinal complications of mitochondrial disease**. *Mitochondrion* 2004, **4**(5-6):601-607.

60. Vedrenne V, Gowher A, De Lonlay P, Nitschke P, Serre V, Boddaert N, Altuzarra C, Mager-Heckel A-M, Chretien F, Entelis N *et al*: **Mutation in PNPT1, which encodes a polyribonucleotide nucleotidyltransferase, impairs RNA import into mitochondria and causes respiratory-chain deficiency**. *Am J Hum Genet* 2012, **91**(5):912-918.

61. Lu C, Wei F, He X, Yao X, Yu C: **LncRNA expression in idiopathic achalasia: New insight and preliminary exploration into pathogenesis**. *Open Med (Wars)* 2022, **17**(1):732-740.

62. Xu M, Jiang B, Man Z, Zhu H: **TRIM37 promotes gallbladder cancer proliferation by activating the Wnt/β-catenin pathway via ubiquitination of Axin1**. *Transl Oncol* 2023, **35**:101732.

63. Wang J, Xu C, Cheng Q, Zhao J, Wu S, Li W, Ma W, Liu C, Jiang X: **RNA Sequencing Revealed Signals of Evolution From Gallbladder Stone to Gallbladder Carcinoma**. *Front Oncol* 2020, **10**:823.

64. Chen X, Chen Y, Huang H-M, Li H-D, Bu F-T, Pan X-Y, Yang Y, Li W-X, Li X-F, Huang C *et al*: **SUN2: A potential therapeutic target in cancer**. *Oncol Lett* 2019, **17**(2):1401-1408.

65. Barahona Ponce C, Scherer D, Brinster R, Boekstegers F, Marcelain K, Gárate-Calderón V, Müller B, de Toro G, Retamales J, Barajas O *et al*: **Gallstones, Body Mass Index, C-Reactive Protein, and Gallbladder Cancer: Mendelian Randomization Analysis of Chilean and European Genotype Data**. *Hepatology* 2021, **73**(5):1783-1796.

66. Maslenkina K, Mikhaleva L, Naumenko M, Vandysheva R, Gushchin M, Atiakshin D, Buchwalow I, Tiemann M: **Signaling Pathways in the Pathogenesis of Barrett's Esophagus and Esophageal Adenocarcinoma**. *International journal of molecular sciences* 2023, **24**(11).

67. Ghatak S, Reveiller M, Toia L, Ivanov AI, Zhou Z, Redmond EM, Godfrey TE, Peters JH: **Bile Salts at Low pH Cause Dilation of Intercellular Spaces in In Vitro Stratified Primary Esophageal Cells, Possibly by Modulating Wnt**

Signaling. *J Gastrointest Surg* 2016, **20**(3):500-509.

68. Takemaru K-I, Yamaguchi S, Lee YS, Zhang Y, Carthew RW, Moon RT: **Chibby, a nuclear beta-catenin-associated antagonist of the Wnt/Wingless pathway**. *Nature* 2003, **422**(6934):905-909.

69. Seki T, Gong L, Williams AJ, Sakai N, Todi SV, Paulson HL: **JosD1, a membrane-targeted deubiquitinating enzyme, is activated by ubiquitination and regulates membrane dynamics, cell motility, and endocytosis**. *J Biol Chem* 2013, **288**(24):17145-17155.

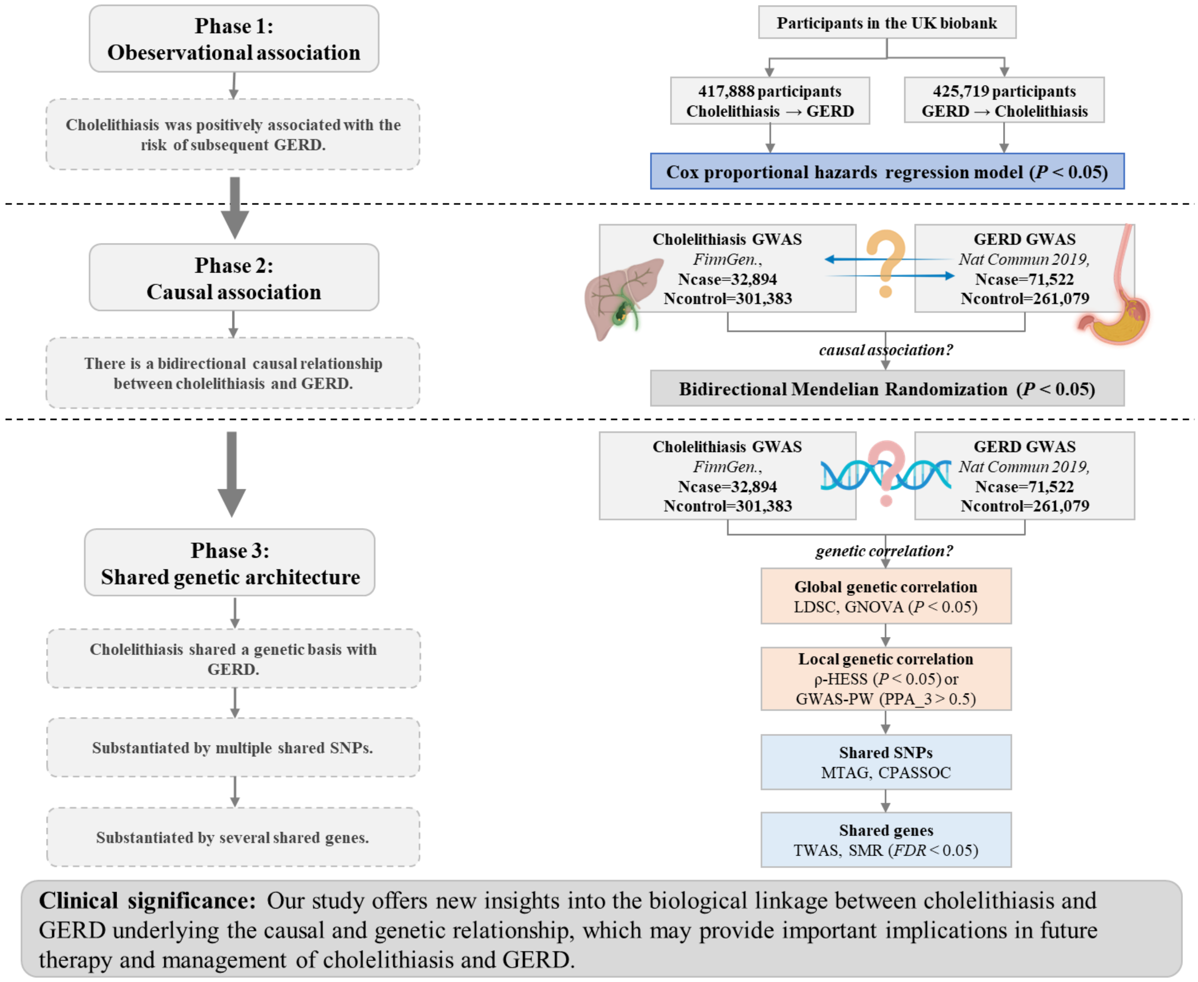

Figure 1. Flowchart of the overall study design. First, we assessed the phenotypic correlations between cholelithiasis and GERD based on the prospective cohort data from UK Biobank. Second, we conducted bidirectional two-sample Mendelian Randomization analysis to investigate the causality by using large-scale GWAS data. Third, we utilized a variety of approaches to dissect the genetic correlations and shared genetic architecture. LDSC and GNOVA methods were applied to detect the global genetic correlation. ρ-HESS and GWAS-PW methods were used to further explore the local genetic correlation. Then, MTAG and CPASSOC methods were employed to find out the shared risk SNPs. Finally, TWAS and SMR methods were utilized to study the shared genes between cholelithiasis and GERD.

GERD: gastroesophageal reflux disease; FDR: false discovery rate; GWAS: genome-wide association study; LDSC: linkage disequilibrium score regression; GNOVA: Genetic covariance analyzer; ρ-HESS: Heritability Estimator from Summary Statistics; GWAS-PW: Pairwise-GWAS; MTAG: multi-trait analysis of GWAS; CPASSOC: Cross-phenotype association test; SMR: Summary data-based Mendelian Randomisation; TWAS: Transcriptome-wide association studies. SNP: single nucleotide polymorphism

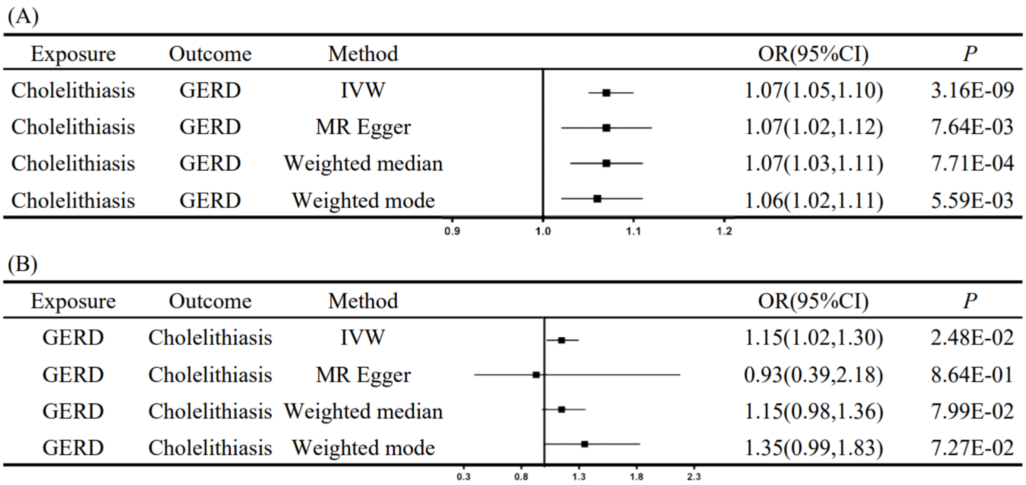

Figure 2. The causal associations between cholelithiasis and GERD. (A). The causal effect of cholelithiasis on GERD; (B). The causal effect of GERD on cholelithiasis. Error bars represent the 95% confidence intervals (CIs) for the estimates. GERD: gastroesophageal reflux disease; CI: confidence interval; IVW: inverse variance weighted.

Table 1. Observational association between cholelithiasis and GERD.

(A)

	Case/person-years	Model 1		Model 2		Model 3	
		**HR (95% CI)**	***P* value**	**HR (95% CI)**	***P* value**	**HR (95% CI)**	***P* value**
**Non-Cholelithiasis**	20,780/2,649,797	1.00 (reference)		1.00 (reference)		1.00 (reference)	
**Cholelithiasis**	1,628/86,654	2.40 (2.30-2.52)	**<0.001**	2.28 (2.17-2.40)	**<0.001**	1.99 (1.89-2.10)	**<0.001**

(B)

	Case/person-years	Model 1		Model 2		Model 3	
		**HR (95% CI)**	***P* value**	**HR (95% CI)**	***P* value**	**HR (95% CI)**	***P* value**
**Non-GERD**	5,883/2,372,960	1.00 (reference)		1.00 (reference)		1.00 (reference)	
**GERD**	1,066/220,497	2.86 (2.70-3.02)	**<0.001**	2.69 (2.54-2.84)	**<0.001**	2.30 (2.18-2.44)	**<0.001**

Table 1. Observational association between cholelithiasis and GERD. (A). Associations of cholelithiasis with the risk of GERD; (B). Associations of GERD with the risk of cholelithiasis.

Model 1: without any adjustments.

Model 2: adjusted for age and sex.

Model 3: adjusted for age, sex, ethnicity, average total annual household income, Deprivation Index, body mass index, alcohol consumption, smoking status, physical activity, education, fresh fruit consumption, raw vegetable consumption, tea consumption, coffee consumption, hypertension, diabetes, renal failure, myocardial infarction, stroke, chronic obstructive pulmonary disease, asthma, anxiety, depression, peptic ulcer.

GERD: gastroesophageal reflux disease; HR: hazard ratio; CI: confidence interval.

Table 2. Heritability and genetic correlation between cholelithiasis and GERD.

		Cholelithiasis	GERD
Heritability (h^2)	LDSC without constraining the intercept	0.0459	0.0679
Heritability (h^2)	LDSC with constraining the intercept	0.0721	0.0761
Heritability (h^2)	GNOVA	0.0659	0.0768
Genetic correlation (r_g), *P*	LDSC without constraining the intercept	0.3053, 2.77×10^{-27}	
Genetic correlation (r_g), *P*	LDSC with constraining the intercept	0.2499, 3.90×10^{-56}	
Genetic correlation (r_g), *P*	GNOVA	0.2625, 2.50×10^{-32}	

GERD: gastroesophageal reflux disease; LDSC: linkage disequilibrium score regression; GNOVA: Genetic covariance analyzer.

Table 3. Genome-wide significant loci shared between cholelithiasis and GERD in cross-trait meta-analyses.

SNP	CHR	BP	Cross-trait meta-analyses		*P*_MTAG		*P*_CPASSOC	Gene
			MTAG	CPASSOC	Cholelithiasis	GERD		
**rs10167227**	2	56004781	-	+	9.28E-06	7.90E-07	**2.93E-08**	*PNPT1**
**rs6742945**	2	53201324	-	+	3.38E-06	1.10E-06	**1.43E-08**	*LOC105369165*
**rs335208**	5	122503245	-	+	2.58E-06	5.66E-07	**6.47E-09**	*PRDM6*
**rs72664027**	8	62948007	-	+	5.03E-06	3.49E-06	**3.89E-08**	*LINC02842*
**rs11537754**	16	570557	-	+	8.29E-07	2.31E-06	**5.95E-09**	*RAB11FIP3*
rs146812426 [a]	2	43909666	+	+	1.26E-93	2.48E-08	3.68E-121	*PLEKHH2*
rs4299376 [a]	2	44072576	+	+	5.88E-124	2.12E-12	2.10E-158	*ABCG8*
rs6733452 [a]	2	44094845	+	+	1.63E-120	9.66E-14	1.04E-151	*ABCG8*
rs7596134 [a]	2	44052833	+	+	8.81E-175	3.78E-15	8.31E-227	*DYNC2LI1, ABCG5*
rs4681515 [a]	3	149212076	+	+	1.35E-48	5.58E-11	3.67E-55	*TM4SF4*
rs9297994 [ab]	8	59392324	+	+	2.25E-40	2.40E-10	1.06E-44	*UBXN2B*、*CYP7A1*
rs10935762 [ab]	3	149216298	+	+	5.12E-42	1.33E-09	1.55E-47	*TM4SF4*
rs3922717 [ab]	6	27030924	+	+	2.16E-08	1.99E-11	1.07E-13	*LOC100270746**
rs12633863 [b]	3	149211512	+	+	2.03E-48	2.63E-10	1.56E-55	*TM4SF4*
rs802036 [b]	7	86977894	+	+	5.58E-21	5.84E-09	1.52E-22	*CROT*

*Genes that interact the SNP through 3D chromatin loops in different cell types.

[a] Independent pleiotropic loci in MTAG and significant in CPASSOC.

[b] Independent pleiotropic loci in CPASSOC and significant in MTAG.

The bolded SNPs represent the independent new loci shared between cholelithiasis and GERD identified in the CPASSOC method.

GERD: gastroesophageal reflux disease; SNP: single nucleotide polymorphism; CHR: chromosome; BP: base pair; MTAG: multi-trait analysis of GWAS; CPASSOC: Cross-phenotype association test.

Figure Legends

Figure 1. Flowchart of the overall study design. First, we assessed the phenotypic correlations between cholelithiasis and GERD based on the prospective cohort data from UK Biobank. Second, we conducted bidirectional two-sample Mendelian Randomization analysis to investigate the causality by using large-scale GWAS data. Third, we utilized a variety of approaches to dissect the genetic correlations and shared genetic architecture. LDSC and GNOVA methods were applied to detect the global genetic correlation. ρ-HESS and GWAS-PW methods were used to further explore the local genetic correlation. Then, MTAG and CPASSOC methods were employed to find out the shared risk SNPs. Finally, TWAS and SMR methods were utilized to study the shared genes between cholelithiasis and GERD.

GERD: gastroesophageal reflux disease; *FDR*: false discovery rate; GWAS: genome-wide association study; LDSC: linkage disequilibrium score regression; GNOVA: Genetic covariance analyzer; ρ-HESS: Heritability Estimator from Summary Statistics; GWAS-PW: Pairwise-GWAS; MTAG: multi-trait analysis of GWAS; CPASSOC: Cross-phenotype association test; SMR: Summary data-based Mendelian Randomisation; TWAS: Transcriptome-wide association studies. SNP: single nucleotide polymorphism

Figure 2. The causal associations between cholelithiasis and GERD. (A). The causal effect of cholelithiasis on GERD; (B). The causal effect of GERD on cholelithiasis. Error bars represent the 95% confidence intervals (CIs) for the estimates. GERD: gastroesophageal reflux disease; CI: confidence interval; IVW: inverse variance weighted.

Supplementary Tables

Supplementary Table 1. Baseline characteristics of UK Biobank participants

Supplementary Table 2. Instrumental variables employed in the primary GWAS data for bidirectional Mendelian Randomization analysis

Supplementary Table 3. Summarized results of bi-directional Mendelian randomization analysis and sensitive analysis about cholelithiasis and gastroesophageal reflux disease in the primary datasets

Supplementary Table 4. Summarized results of bi-directional Mendelian randomization analysis and sensitive analysis about cholelithiasis and gastroesophageal reflux disease in the replication datasets

Supplementary Table 5. Characteristics of the regions of the genome where genetic correlation between cholelithiasis and gastroesophageal reflux disease detected by ρ_HESS and GWAS-PW

Supplementary Table 6. Independent genome-wide significant loci shared between cholelithiasis and gastroesophageal reflux disease in MTAG

Supplementary Table 7. Independent genome-wide significant loci shared between cholelithiasis and gastroesophageal reflux disease in CPASSOC

Supplementary Table 8. TWAS-prioritized genes associated with cholelithiasis and gastroesophageal reflux disease

Supplementary Table 9. SMR-prioritized genes associated with cholelithiasis and gastroesophageal reflux disease

Supplementary Figure The flowchart of the prospective cohort study

Figure 2

Click here to access/download;Figure;Figure 2.jpg

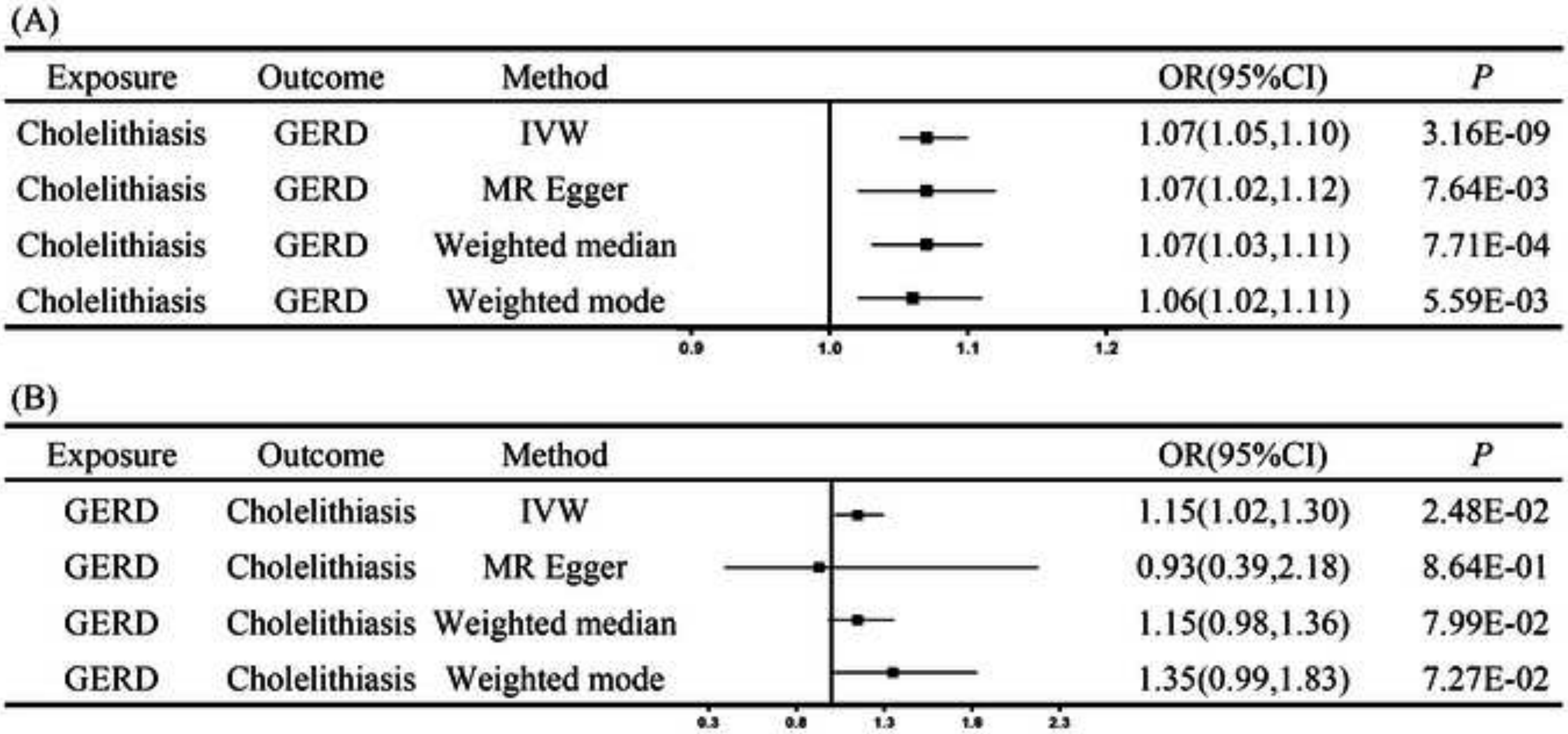

Figure 1 Click here to access/download;Figure;Figure 1.jpg

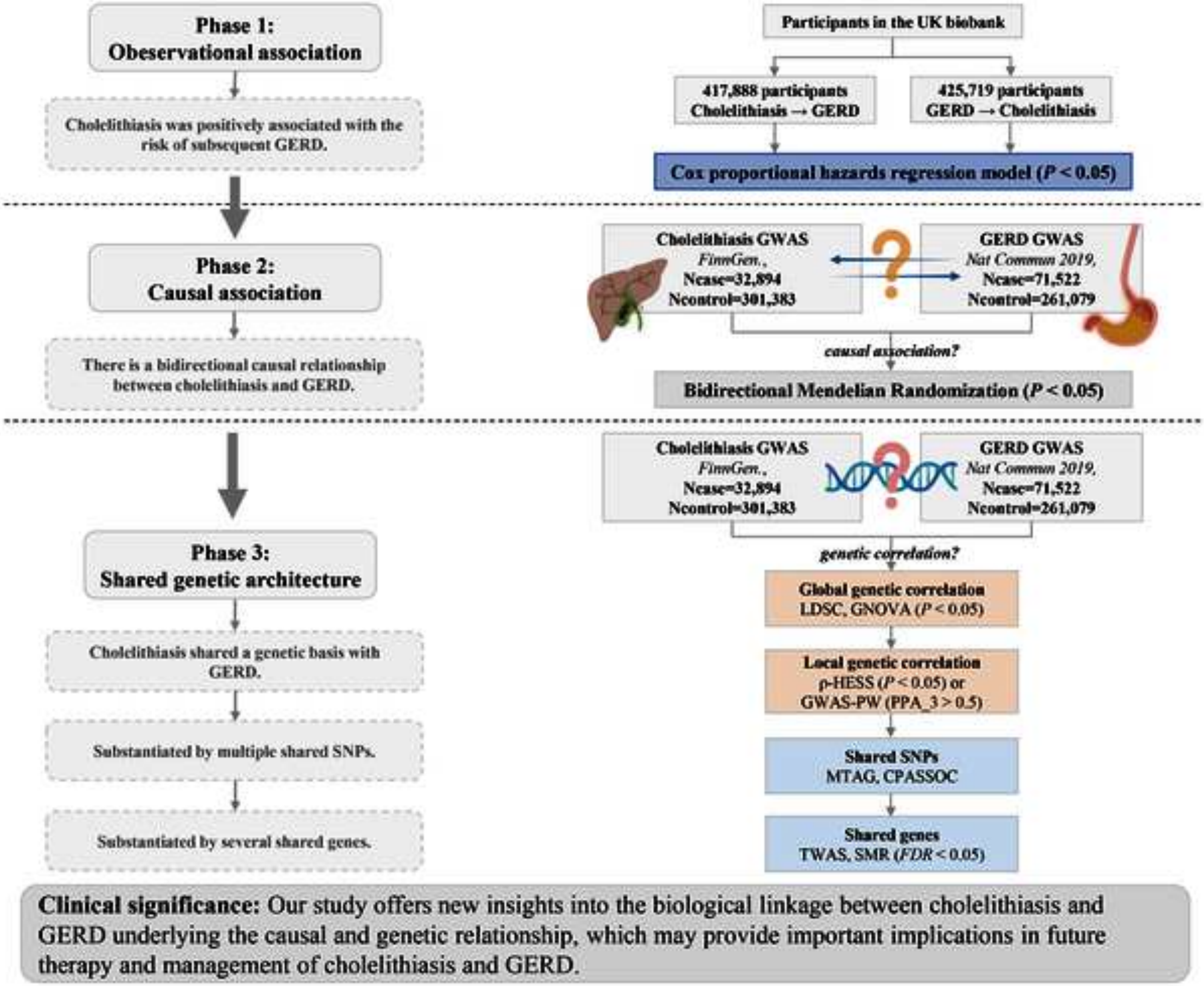

Click here to access/download
Supplementary Material
Supplementary tables.docx

Click here to access/download
Supplementary Material
Supplementary Figure.pdf

Personal Cover

Click here to access/download;Personal Cover;cholelithiasis-GERD_cover letter_V2.docx

Department of Gastroenterology,
Guangdong Academy of Medical Sciences/Guangdong Provincial People's Hospital
Guangzhou, Guangdong, 510000

Prof. Hao Chen
Email: chenhao@gdph.org.cn

Dear Editors of ***GigaScience***,

We would like to submit the enclosed manuscript entitled "**Observational, Causal Relationship and Shared Genetic Basis Between Cholelithiasis and Gastroesophageal Reflux Disease: Evidence from a Cohort Study and Comprehensive Genetic Analysis**" in ***GigaScience***, and we sincerely wish that our manuscript would have the opportunity to in-depth review.

Graphical abstract of the manuscript

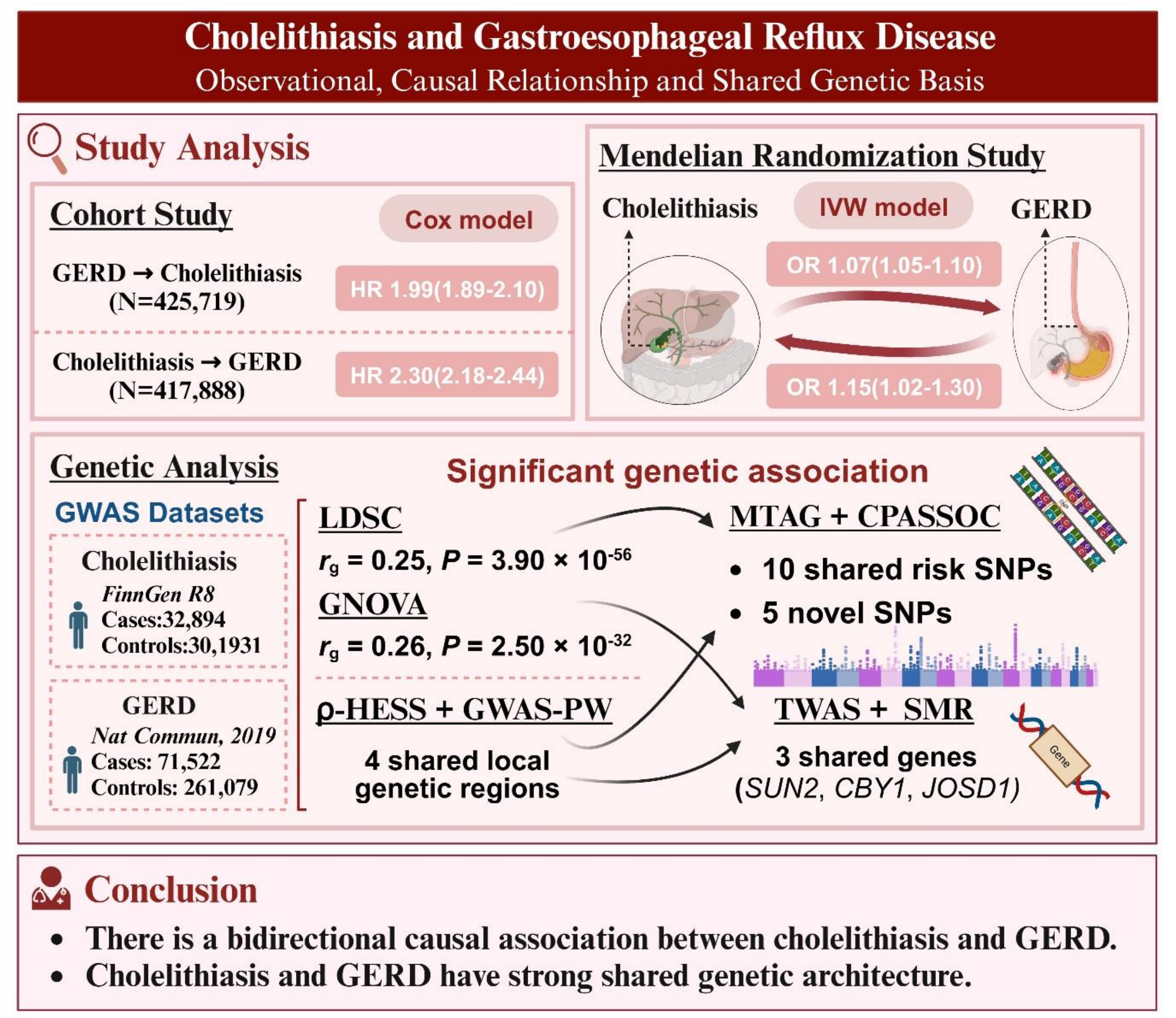

1. **The priority area:** Both cholelithiasis and gastroesophageal reflux disease (GERD) are prevalent diseases, sharing numerous common risk factors and being linked to

Department of Gastroenterology, **Prof. Hao Chen**
Guangdong Academy of Medical Sciences/Guangdong Provincial People's Hospital
Guangzhou, Guangdong, 510000 **Email: chenhao@gdph.org.cn**

abnormalities in gastrointestinal motility. However, the associations between these two diseases are poorly understood. A comprehensive understanding of this connection is vital for the enhanced management of patients afflicted with cholelithiasis and/or GERD.

2. **Critical gaps in knowledge:** Previous studies on the relationship between cholelithiasis and GERD often suffered from small sample size as well as outdated data, leading to inconsistent conclusions (**Table 1**). Besides, **no research has evaluated the causal and genetic correlation between these two diseases**.

Table 1: Published observational studies about cholelithiasis and GERD

First author, year	Journal	Correlation estimates	Risk
Unalp-Arida A, 2023	*J Gastrointest Surg*	40% cholelithiasis patients had concurrent GERD	↑
Avidan B, 2001	*Am J Gastroenterol*	Cholelithiasis → GERD 1.02 (0.68-1.51)	-
Portincasa P, 1997	*Eur J Clin Invest*	75% GERD in cholelithiasis cases vs 15% GERD in controls ($P < 0.05$)	↑
Morton JM, 2002	*J Gastrointest Surg*	Gallbladder dysfunction 58% in cases with GERD vs 3% in healthy individuals	↑

3. **New understanding:**

1) Our study is **the first** to identify cholelithiasis and GERD as **mutual risk factors** using a large-scale prospective cohort study and further established **a bidirectional causal relationship** between them using the GWAS datasets for Mendelian randomization analysis.

2) We found **a significant genetic correlation** (LDSC: $r_g = 0.31$, $P = 2.77 \times 10^{-27}$) between cholelithiasis and GERD **for the first time**, providing evidence that genetic factors play an important role in the pathogenesis of the association of these two diseases.

Department of Gastroenterology, **Prof. Hao Chen**
Guangdong Academy of Medical Sciences/Guangdong Provincial People's Hospital
Guangzhou, Guangdong, 510000 **Email: chenhao@gdph.org.cn**

3) A total of **5 novel shared risk SNPs** (rs10167227, rs6742945, rs335208, rs72664027, rs11537754) and **3 critical shared genes** (*CBY1*, *SUN2*, *JOSD1*) were identified.

4. Critical impacts on clinical practice:

1) The bidirectional causal relationship between cholelithiasis and GERD highlights the need to delve into underlying mechanisms and reiterate the importance for clinicians to consider the cooccurrence of cholelithiasis and GERD.
2) The significant genetic association, shared SNPs and shared genes offer novel insights into the genetic architecture shared between cholelithiasis and GERD, **suggesting promising therapeutic targets** for cholelithiasis and GERD.
3) Our research could stimulate further studies on their linkage and might provide an innovative research direction for future therapeutic strategies and management of cholelithiasis and GERD.

5. Declaration of Interest statement:

The authors declare that they have no competing interests.

6. Suggested reviewers:

We suggest the following researchers to be reviewers for our manuscript, who are experts in the related field and could give fair judgment on our work.

1. Prof. Jiande D. Z. Chen (jiandedzchen@gmail.com) from Division of Gastroenterology and Hepatology, School of Medicine, University of Michigan, USA.

2. Prof. Waheed-Ul-Rahman Ahmed (waheed.ahmed@lmh.ox.ac.uk) from Botnar Research Centre, Nuffield Department of Orthopaedics, Rheumatology and Musculoskeletal Sciences, University of Oxford, UK.

Department of Gastroenterology, **Prof. Hao Chen**
Guangdong Academy of Medical Sciences/Guangdong Provincial People's Hospital
Guangzhou, Guangdong, 510000 **Email: chenhao@gdph.org.cn**

3. Prof. Bang Zheng (Bang.Zheng@lshtm.ac.uk) from Usher Institute, University of Edinburgh, UK.

4. Prof. Feng Liu (feng.liu@wolfson.ox.ac.uk) from Botnar Research Centre, Nuffield Department of Orthopaedics, Rheumatology and Musculoskeletal Sciences, University of Oxford, UK.

5. Prof. Jianrong He (jianrong.he@bigcs.org) from Division of Birth Cohort Study, Guangzhou Women and Children's Medical Center, Guangzhou Medical University, China.

6. Prof. Yuliang Feng (yuliang.feng@seh.ox.ac.uk) from Department of Genome Sciences, Jackson Laboratory, USA.

7. Prof. Liuyang Cai (caily@sustech.edu.cn) from Department of Pharmacology, School of Medicine, Southern University of Science and Technology, Shenzhen, Guangdong Province 518055, China

8. Prof. Anil Jegga (Anil.Jegga@cchmc.org) from Biomedical Informatics, Cincinnati Children Hospital Medical Centre, Cincinnati, Ohio, United States of America

Our team is comprised of researchers in gastroenterology, hepatology, and epidemiology, dedicated to uncovering the risk factors associated with digestive disorders like cholelithiasis and gastroesophageal reflux disease, while also delving into the shared genetic foundations of these conditions. Our works have been published in ***Nature, BMJ, Gut, Hepatology, Nature Machine Intelligence, Nature Communications, EClinicalMedicine, EBioMedicine, Brief Bioinform, BMJ Ment Health*** [1-10], etc.

We confirm that this manuscript has not been published and is not under consideration for publication elsewhere. We would be deeply appreciative if you could offer an opportunity for in-depth review of our manuscript in ***GigaScience.***

Thank you for your consideration.

Department of Gastroenterology, **Prof. Hao Chen**
Guangdong Academy of Medical Sciences/Guangdong Provincial People's Hospital
Guangzhou, Guangdong, 510000 **Email: chenhao@gdph.org.cn**

Sincerely,

Felix W Leung

David Geffen School of Medicine, University of California Los Angeles

Los Angeles 90095

California, USA

Email: felixleung@socal.rr.com

Hao Chen

Guangdong Provincial People's Hospital (Guangdong Academy of Medical Sciences), Southern Medical University

Guangzhou 510080, China

E-mail: chenhao@gdph.org.cn

Publications (*Correspondence)

1. G. Zhang*, X. Fang, X. Guo*, L. Li, **R. Luo***, …, Ji. Wang, Y. Yin*, Ju. Wang*. "The oyster genome reveals stress adaptation and complexity of shell formation." ***Nature***. vol. 490,7418 (2012): 49-54. doi:10.1038/nature1141
2. Luo D, Cheng Y, Zhang H, Ba M, Chen P, Li H, Chen K, Sha W*, Zhang C*, **Chen H***. Association between high blood pressure and long-term cardiovascular events in young adults: systematic review and meta-analysis. ***BMJ***. 2020 Sep 9;370:m3222. doi: 10.1136/bmj.m3222.
3. Zeng R, Sha W, Wang J, Zhuo Z, Wu H, Leung FW*, **Chen H***. Evaluation of proton pump inhibitors and risks of gastric cancer. ***Gut***. 2021 Nov 26: gutjnl-2021-326291. doi: 10.1136/gutjnl-2021-326291.
4. Zeng R, Sha W, Leung FW*, **Chen H***. Proton Pump Inhibitors and Biliary Tract Cancer Risk:

Department of Gastroenterology, **Prof. Hao Chen**
Guangdong Academy of Medical Sciences/Guangdong Provincial People's Hospital
Guangzhou, Guangdong, 510000 **Email: chenhao@gdph.org.cn**

Causality or Reverse Causality. ***Hepatology***. 2021 Aug 5. doi: 10.1002/hep.32090.

5. **Luo, R.**, Wong, CL., Wong, YS. et al. Exploring the limit of using a deep neural network on pileup data for germline variant calling. ***Nat Mach Intell*** 2, 220–227 (2020). doi.org/10.1038/s42256-020-0167-4
6. **Luo R**, Sedlazeck FJ, Lam TW, Schatz MC. A multi-task convolutional deep neural network for variant calling in single molecule sequencing. ***Nat Commun***. 2019 Mar 1;10(1):998. doi: 10.1038/s41467-019-09025-z.
7. Zhang C, Cheng Y, Luo D, Wang J, Liu J, Luo Y, Zhou Y, Zhuo Z, Guo K, Zeng R, Yang J, Sha W*, **Chen H***. Association between cardiovascular risk factors and colorectal cancer: a systematic review and meta-analysis of prospective cohort studies. ***EClinicalMedicine***. Apr 2021. Doi: 10.1016/j.eclinm.2021.100794.
8. Ruiie Zeng, Rui Jiang, Wentao Huang, Jiaxuan Wang, Lijun Zhang, Yuying Ma, Yanjun Wu, Mejun Meng, Hekui Lan, Qizhou Lian, Felix W. Leung*, Weihong Sha*, **Hao Chen*.** Dissecting shared genetic architecture between obesity and multiple sclerosis. ***EBioMedicine***. 2023 Jul; 93:104647. doi: 10.1016/j.ebiom.2023.104647.
9. Su J, Zheng Z, Ahmed SS, Lam TW, **Luo R**. Clair3-trio: high-performance Nanopore long-read variant calling in family trios with trio-to-trio deep neural networks. ***Brief Bioinform***. 2022 Sep 20;23(5):bbac301. doi: 10.1093/bib/bbac301.
10. Tong S, Lyu Y, Huang W, Zeng R, Jiang R, Lian Q, Leung FW*, Sha W*, **Chen H***. Genetically predicted causal associations between periodontitis and psychiatric disorders. ***BMJ Ment Health.*** 2023 Nov 22;26(1):e300864. doi: 10.1136/bmjment-2023-300864.

Graphical Abstract

Click here to access/download;Graphical Abstract;graph abstract.jpg

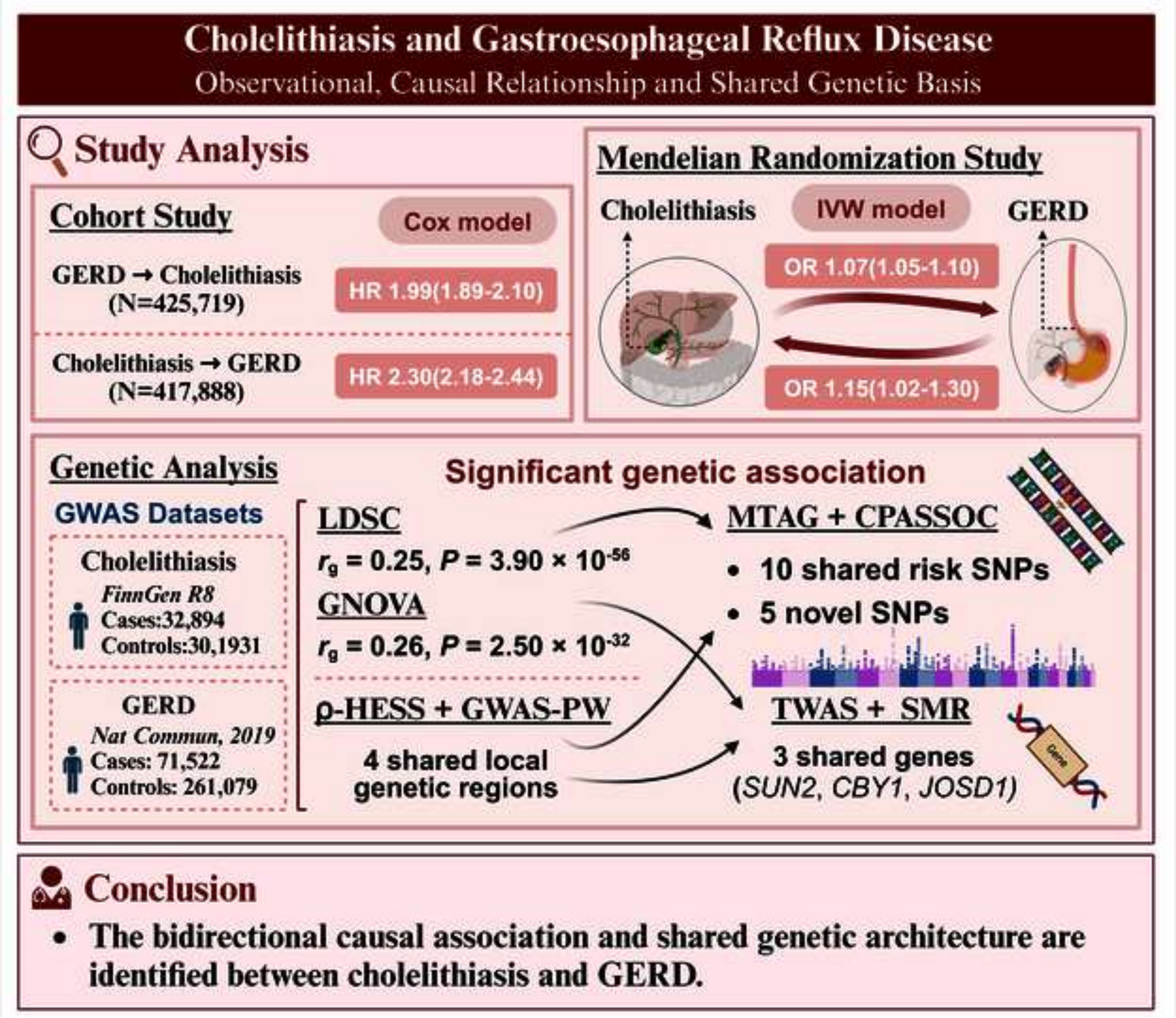

Supplement: giaf023_GIGA-D-24-00123_Original_Submission [file giaf023_giga-d-24-00123_original_submission.pdf]
