# Supplementary material for: Observational, causal relationship and shared genetic basis between cholelithiasis and gastroesophageal reflux disease: evidence from a cohort study and comprehensive genetic analysis
Source: Gigascience. 2025 Mar 26;14:giaf023. doi: 10.1093/gigascience/giaf023 (PMC11943489; doi:10.1093/gigascience/giaf023)
Supplement: giaf023_Response_to_Reviewer_Comments_Original_Submission [file giaf023_response_to_reviewer_comments_original_submission.pdf]

Author's Response To Reviewer Comments

Dear Editors and Reviewers of GigaScience,

We are grateful for being provided with the opportunity to revise our manuscript, and we sincerely appreciate all the comments and recognitions from the editors and reviewers, which help us improve our manuscript, “Observational, Causal Relationship and Shared Genetic Basis Between Cholelithiasis and Gastroesophageal Reflux Disease: Evidence from a Cohort Study and Comprehensive Genetic Analysis” (GIGA-D-24-00123).

With careful analysis and revision to the manuscript, we hereby provide our responses point-by-point (marked in blue font) after taking all the critiques into account. For highlighting the revisions that we have made, all the revised parts for point-to-point responses are highlighted with Track Changes in the manuscript (Manuscript - Marked.docx). The detailed response letter, including tables and figures, can be found in the file (gigascience_response letter1.docx).

Once again, thank you very much for your careful evaluation and kind suggestions for our work. We sincerely hope that our manuscript in its present form is suitable for being given the precious opportunity for publication in GigaScience.

Yours sincerely,
Felix W. Leung
David Geffen School of Medicine, University of California Los Angeles, Los Angeles, California, USA; Email: Felix.Leung@va.gov
Hao Chen
Department of Gastroenterology and Hepatology, Guangdong Academy of Medical Sciences/Guangdong Provincial People’s Hospital, Guangzhou, China. Email: chenhao@gdph.org.cn
On behalf of co-authors.

Reviewer #1 (Comments to the Author):
Lyu and coworkers have investigated both observational and genetic links between a gallstone known as cholelithiasis and gastroesophageal reflux disease (GERD), leveraging UK Biobank datasets. The authors observed a significant, modest positive genetic correlation between these two traits. In addition, authors have identified some shared loci between two traits via cross-trait meta-analysis. The causal relationship was assessed by Mendelian randomisation (MR). Gene imputation methods TWAS and SMR were also performed, showing shared genes between two traits. Although the presented manuscript is an interesting premise for study, the biological implications of the results underlying cholelithiasis and GERD are lacking.
Authors’ Response:
We sincerely appreciate your recognition of the manuscript as presenting a compelling premise for study. In response to your concerns regarding the absence of biological implications underlying the findings related to cholelithiasis and GERD, we have made significant revisions and provided additional insights. To gain shared biological insights into cholelithiasis and GERD, we conducted functional annotation of the pleiotropic SNPs and shared genes using multiple methods. We utilized the knowledge-based databases Kyoto Encyclopedia of Genes and Genomes (KEGG) and Gene Ontology (GO) to perform pathway enrichment analyses for identifying pathways associated with these genes. In addition, we utilized the STRING database (https://cn.string-db.org/) to find the interactions mapped to the pleiotropic SNPs and shared functional genes. Based on the above analysis, we found that the shared loci are enriched in bile acid and lipid metabolism pathways, while the shared genes are enriched in the Wnt signaling pathway. According to your kind comments, we have revised our manuscript point-to-point as follows (marked in blue font) and highlighted the revised parts with tracked changes in the manuscript (Manuscript - Marked.docx).

Some comments are provided to consider:
Page 14. "genetically predicted GERD could increase the risk of cholelithiasis by 15% (OR = 1.15, 95%CI =

1.02-1.30, P = 0.025) according to IVW method (Figure 2B)". This is likely cherry picking, as this association signal is not picked up by the other methods.
Authors' Response:
Thank you for your reminder. The association signal from GERD to cholelithiasis was statistically significant using the IVW and weighted median methods after excluding the confounding SNP, as per your suggestions, but it could not be confirmed using the MR-Egger and the weighted mode methods. In addition, the results were replicated in an independent GERD dataset, enhancing the robustness of our findings. We have to acknowledge this as a limitation of our study and have revised the results and limitation sections accordingly, adopting a more conservative way in drawing conclusions.
"After excluding the confounding IVs, we used forty-six cholelithiasis-associated and twenty GERD-associated genetic instruments (Supplementary Table 2), respectively in the analyses and provided evidence for the causal association between cholelithiasis and GERD. …… Besides, we also conducted reverse MR analysis and found that genetically predicted GERD could increase the risk of cholelithiasis by 15% (OR = 1.15, 95%CI = 1.02-1.31, P = 0.027) according to IVW method (Figure 2B, Supplementary Table 3). This association was further validated through analyses employing the weighted median method and an additional dataset (Supplementary Tables 3-4), although it could not be confirmed using the MR-Egger and the weighted mode methods." (Page 15, Line 337-349)
"First, the causal relationship from GERD to cholelithiasis was not significant in all sensitivity analyses, which may be attributed to the limitations of GWAS statistics. Therefore, larger and more powerful GWAS data for cholelithiasis and GERD will be needed to definitively establish the causal relationships from GERD to cholelithiasis." (Page 21, Line 522-526)
"In summary, we found a bidirectional association between cholelithiasis and GERD, which may be attributed to a bidirectional causal relationship and a shared genetic basis including the significant genetic correlation, novel shared loci and genes." (Page 22, Line 533-535)

The genetic correlation using LDSC and/or the local correlation-based method p-HESS reported in this study seems modest. The liability scale of reported heritability (presented in Table 2) for both cholelithiasis and GERD was not shown. The local genetic correlation method identified eight correlated genomic loci between two traits. How was the MHC region (the most complicated genomic region) handled? Did the authors use LD-based clumping? Further discussion is necessary about genetic correlation and to what extent these correlated genetic factors might influence these two traits.
Authors' Response:
Thank you for the insightful comments. We have added the liability scale of reported heritability for cholelithiasis and GERD in Table 2 and have revised the methods and results sections accordingly.
"Based on the prevalence rates of 20% [1] for cholelithiasis and 17.1% [6] for GERD, we calculated the liability scale of the reported heritability for both traits." (Page 11, Line 233-235)
"SNP-based liability-scale heritability h^2 for cholelithiasis and GERD were 26.65% and 14.01% when utilizing the univariate LDSC with constraining the intercept." (Page 15, Line 362-363)
We included the MHC region (chromosome 6: 28,477,797-33,448,354) in our analysis; however, as no significant local heritability was detected in this area, we did not conduct further analysis on this region (Supplementary Table 5).
Regarding LD-based clumping, the original article describing the method states that the selected regions are already approximately LD-independent [doi: 10.1016/j.ajhg.2017.09.022]. Therefore, we did not apply additional LD-based clumping.
"Then, we explored the local SNP heritability for each trait and estimated the local genetic correlation in 1,613 approximately LD-independent regions [33]." (Page 11, Line 249-251)
Further discussion on the genetic correlation and the extent to which these shared genetic factors may influence the two traits has been added as follows.
"In the analysis of heritability and genetic correlation, the heritability of cholelithiasis and GERD was estimated to be 17% and 13%, respectively, indicating a significant genetic contribution to the etiology of both diseases, consistent with previous studies [15, 54]. The global genetic correlation between cholelithiasis and GERD was found to be 0.31, suggesting a moderate to strong genetic association between these conditions. The finding supports the hypothesis that genetic factors, such as local genetic correlations, shared loci, and common functional genes, play an important role in the co-occurrence of cholelithiasis and GERD." (Page 19, Line 451-458)

Regarding the causality test by MR, I wonder how the confounders of these two traits were considered. Are

there other sources of pleiotropy in the selected variants, as instruments can act via different pathways such as obesity, smoking, or dietary preferences? Is there any evidence to support that the confounders were minimised by the selection of instruments?
Authors’ Response:
Thank you for your detailed review and valuable comments. We primarily considered the confounding factors between the two traits through sensitivity analyses, including heterogeneity and pleiotropy tests. The p-values for both sensitivity analyses were greater than 0.05, suggesting a low likelihood of heterogeneity and pleiotropy among the IVs. Based on your suggestion, we conducted a search for the selected IVs in the GWAS catalog and identified a few IVs associated with BMI. We identified one SNP (rs6762606) in GERD GWAS data associated with BMI-adjusted waist circumference [doi: 10.1038/s41598-021-89176-6] and one SNP (rs28929474) in cholelithiasis GWAS data associated with BMI-adjusted hip circumference [doi: 10.1038/s41598-021-89176-6] and body fat percentage [doi: 10.3389/fendo.2023.1274791]. After excluding these IVs and reanalyzing the data, the causal relationships estimated by the IVW method from cholelithiasis to GERD and GERD to cholelithiasis remained significant. The revised methods, results and updated figures are presented as follows.
“The MR study indicated the causal effect of genetic liability to cholelithiasis on the incidence of GERD (Odds ratio (OR) = 1.08, 95% CI = 1.05-1.11) and the causal effect of genetic predicted GERD on cholelithiasis (IVW OR = 1.15, 95% CI = 1.02-1.31).” (Page 4, Line 69-72)
“Additionally, we searched the instrumental variables in the GWAS catalog (https://www.ebi.ac.uk/gwas/) to identify potential confounders like BMI, smoking and certain dietary habits, and excluded the confounding variants from further analyses.” (Page 10, Line 202-205)
“After excluding the confounding IVs, we used forty-six cholelithiasis-associated and twenty GERD-associated genetic instruments (Supplementary Table 2), respectively in the analyses and provided evidence for the causal association between cholelithiasis and GERD. Genetically determined cholelithiasis has the possibility to increase the risk of GERD by 8% (IVW OR = 1.08, 95%CI = 1.05-1.11, P = 3.70 × 10-10, Figure 2A, Supplementary Table 3), which was further validated by other three MR methods and the analyses with a supplementary dataset (Supplementary Table 4). Besides, we also conducted reverse MR analysis and found that genetically predicted GERD could increase the risk of cholelithiasis by 15% (OR = 1.15, 95%CI = 1.02-1.31, P = 0.027) according to IVW method (Figure 2B, Supplementary Table 3). This association was further validated through analyses employing the weighted median method and an additional dataset (Supplementary Tables 3-4), although it could not be confirmed using the MR-Egger and the weighted mode methods.” (Page 15, Line 337-349)

According to the author's statement in the introduction (page 6), causality can be reversed. While doing MR analyses, did the authors perform reverse MR? The authors mentioned bidirectional causal relations were detected (in the conclusion section), but that was not clear from reading the method or results section. According to supplementary Table 3, a two-way causal relationship between cholelithiasis and GERD was observed. However, a sensitivity analysis revealed no significant causal association between them. This aspect of the results should be acknowledged in the results and discussion section.
Authors’ Response:
Thank you for your reminder. We conducted a bidirectional MR analysis and have revised the methods and results sections to better clarify this issue.
The causal relationship from cholelithiasis to GERD remained significant across all sensitivity analyses. The causal relationship from GERD to cholelithiasis became significant in both the IVW and weighted median methods after excluding SNPs potentially influenced by confounding factors, while other sensitivity analyses did not detect significant associations. Therefore, the causal relationship from GERD to cholelithiasis requires further investigation with better GWAS datasets. Accordingly, we have revised the results and discussion sections as follows.
“We performed the bidirectional MR analysis to explore the potential causal relationship between cholelithiasis and GERD, using R packages “TwoSampleMR” [18], and “MR-PRESSO” [19] in R software (version 4.2.1).” (Page 9-10, Line 194-197)
“Besides, we also conducted reverse MR analysis and found that genetically predicted GERD could increase the risk of cholelithiasis by 15% (OR = 1.15, 95%CI = 1.02-1.31, P = 0.027) according to IVW method (Figure 2B, Supplementary Table 3). This association was further validated through analyses employing the weighted median method and an additional dataset (Supplementary Tables 3-4), although it could not be confirmed using the MR-Egger and the weighted mode methods.” (Page 15, Line 343-349)
“First, the causal relationship from GERD to cholelithiasis was not significant in all sensitivity analyses,

which may be attributed to the limitations of GWAS statistics. Therefore, larger and more powerful GWAS data for cholelithiasis and GERD will be needed to establish the causal relationships from GERD to cholelithiasis.” (Page 21, Line 522-526)

Regarding transcriptomic imputation-based analyses (TWAS and SMR), I did not see a follow-up colocalization test was performed. Is there any evidence to explain that the same variant is sufficient to influence gene expression and traits as predicted through TWAS?
Authors’ Response:
Thank you for your question. The colocalization analysis has been included in our study and there is evidence to explain that the same variant is sufficient to influence gene expression and traits. Please allow me to elaborate it as follows:
Two primary methodological approaches have been developed for colocalization: proportional colocalization and enumeration colocalization [doi: 10.1016/j.ajhg.2022.04.001]. In proportional colocalization, the null hypothesis is proportionality of the genetic associations with the two traits. If there is colocalization, we would expect marginal genetic associations with the two traits to be proportional provided that either there is a single causal variant (in which case the genetic associations would be determined by their correlation with the causal variant) or the traits are on the same causal pathway and all variants primarily influence the same upstream trait (which may be one of the traits under analysis, or an unmeasured trait). If there is evidence against the statistical model that the genetic associations are proportional, then we conclude that there is lack of colocalization. Otherwise, we conclude that there is colocalization. The heterogeneity in dependent instruments (HEIDI) test, utilized within the SMR software [doi: 10.1038/ng.3538], belongs to the family of proportional colocalization methods. This test employs multiple SNPs within a cis-eQTL region to differentiate between pleiotropy and linkage. Under the pleiotropy hypothesis, wherein gene expression and a trait share a common causal variant, the bxy values calculated for any SNPs in linkage disequilibrium (LD) with the causal variant are expected to be identical. Therefore, testing the null hypothesis of a single causal variant is equivalent to assessing the heterogeneity of the bxy values estimated for the SNPs within the cis-eQTL region. For each probe that meets the genome-wide significance threshold in the SMR test, the HEIDI method is employed to evaluate the heterogeneity of bxy values for multiple SNPs in the cis-eQTL region. A HEIDI test P-value greater than 0.05 indicates insufficient evidence to reject the null hypothesis of a single causal variant influencing both gene expression and trait variation, suggesting the presence of colocalization. Moreover, the literature concerning the SMR method identifies three possible explanations for positive SMR results: causation, pleiotropy, and linkage (as illustrated in the figure below). The HEIDI test plays a critical role in distinguishing between pleiotropy and linkage. If the HEIDI test yields a P-value greater than 0.05 and the SMR results are significant, this suggests that a single variant is sufficient to influence both gene expression and the trait.
Our results indicate that the p-values for the HEIDI test for the three shared genes are all greater than 0.05 (Supplementary Table 11), suggesting that the same variant is sufficient to influence both gene expression and the traits.

I would encourage authors to avoid using the term 'causal' (as emphasised in the conclusion section). Still larger datasets in the future relevant to cholelithiasis and GERD will be required to establish the causality between the two investigated traits. Furthermore, the conclusion section claims that biological mechanisms were explored between these two traits. There are plenty of bioinformatics analyses, such as biological pathway analyses, gene ontology, and biomolecular network analyses, which might be employed to support that conclusion. Unfortunately, I note this is the very weak part of the current manuscript; it lacks a thorough presentation of the biological implications of the findings underpinning cholelithiasis and GERD. Therefore, instead of presenting a series of statistical analyses, describing to what extent and how the presented results can explain the biology of the disease and its aetiology will strengthen the manuscript.
Authors’ Response:
We sincerely appreciate your valuable insights, which have greatly contributed to improving our manuscript. While Mendelian randomization analysis is a relatively reliable method for identifying potential causal relationships between diseases, we acknowledge that establishing causality remains challenging. Larger datasets and further experimental studies are warranted to explore the causal correlation between cholelithiasis and GERD more thoroughly. In light of this, we have revised our conclusion to state that “a potential causal relationship has been identified.”
“In summary, we found a bidirectional association between cholelithiasis and GERD, which may be attributed to a bidirectional causal relationship and a shared genetic basis including the significant genetic

correlation, novel shared loci and genes.” (Page 22, Line 533-535)
Additionally, in line with your suggestions, we have incorporated KEGG and GO pathway enrichment analyses, as well as biomolecular network analyses, to further elucidate the potential mechanisms underlying the comorbidity between the two diseases. Corresponding revisions have been incorporated into the methods, results, and discussion sections, as outlined below:
“The pathway enrichment analyses and biomolecular network analyses
To gain shared biological insights into cholelithiasis and GERD, we conducted functional annotation of the pleiotropic SNPs and shared genes using multiple methods. We utilized the knowledge-based databases Kyoto Encyclopedia of Genes and Genomes (KEGG) and Gene Ontology (GO) to perform pathway enrichment analyses for identifying pathways associated with these genes, using the ClusterProfiler R package (https://bioconductor.org/packages/release/bioc/html/clusterProfiler.html)[45]. P values from the pathway enrichment analyses were adjusted for multiple comparisons through the FDR approach. In addition, we utilized the STRING database (https://cn.string-db.org/) [46] to find the interactions mapped to the pleiotropic SNPs and shared functional genes. ” (Page 13, Line 310-320)
“After multiple corrections, the pathway enrichment analysis using KEGG database identified 5 pathways according to above genes, including cholesterol metabolism, bile secretion, fat digestion and absorption, ABC transporters, primary bile acid biosynthesis (Figure 3A, Supplementary Table 8). The pathway enrichment analysis using GO database identified 65 biological processes, 2 cellular components and 8 molecular functions, most of these pathways are related to lipid and bile acid metabolism (Figure 3B, Supplementary Table 9). In the network analysis, we observed a close association among TM4SF4, CYP7A1, ABCG5 and ABCG8 (Figure 3C).” (Page 17, Line 393-401)
“Using the KEGG database, we found two significantly enriched pathways, Wnt signaling pathway (CBY1) and cytoskeleton in muscle cells (SUN2) (Figure 4A, Supplementary Table 12). Wnt signaling pathway (CBY1) also enriched significantly in the GO pathway enrichment analysis, which was shown in Figure 4B and Supplementary Table 13. In the network analysis, we did not identify the association between these three shared genes.” (Page 17, Line 417-422)
“According to the results of pathway enrichment analyses, the genes associated with these loci were enriched in pathways related to lipid and bile acid metabolism, including cholesterol metabolism, bile secretion, ABC transporters, primary bile acid biosynthesis. Several studies have reported that aberrant lipid and bile acid metabolism contributes to the development of both cholelithiasis and GERD [1, 57-59].” (Page 19-20, Line 475-479)
“Furthermore, existing studies suggested the involvement of bile acids in GERD progression through the activation of the Wnt/β-catenin pathway [72]. CBY1 might be involved in the linkage between gallstone disease and GERD, as it can inhibit the Wnt/β-catenin pathway [73], which was enriched according to the results of the pathway enrichment analyses.” (Page 20-21, Line 504-508)

I also believe that the transcriptomic imputation-based findings (TWAS, SMR) would also need to be validated, as these methods impute gene expression using GWAS and eQTLs. These eQTL datasets were obtained from normal individuals; therefore, it is uncertain whether the predicted gene expression will correlate with real gene expression in cholelithiasis and GERD patients. Are there any existing gene expression datasets, such as microarrays or RNA sequencing datasets, available for cholelithiasis and GERD? If so, authors could look at how predicted expression correlates with differential gene expression in cholelithiasis and GERD patients compared to controls.
Authors’ Response:
Thank you for your suggestion. There are limited studies and datasets available on gene expression related to cholelithiasis and GERD. We conducted a differential gene expression analysis using the existing datasets for these diseases. As shown below, these three genes do not exhibit differential expression in the corresponding gallbladder or esophagus tissues. The relatively small sample sizes (GSE148381, 7 controls and 6 cases with GERD; GSE66430, 3 healthy controls and 1 case with chronic gallstones) in these expression datasets might limit their statistical power. Therefore, further evidence is needed to establish a potential association between these genes and cholelithiasis or GERD in future studies.
The results of differential gene expression analysis:
GSE148381 GERD-normal

GeneID	padj	pvalue	lfcSE	stat	log2FoldChange	gene_symbol
9929	0.882331	0.368871	0.209281	0.898589	0.188057492	JOSD1
25776	0.899131	0.405207	0.121985	-0.832357	-0.101535494	CBY1
25777	0.602641	0.092314	0.195587	-1.683317	-0.329234529	SUN2

GSE66430 cholelithiasis-normal

GeneID	padj	pvalue	lfcSE	stat	log2FoldChange	gene_symbol
9929	0.3656	0.035094	0.413655	2.107272	0.871683488	JOSD1
25776	0.964659	0.467666	0.443544	0.726282	0.322137773	CBY1
25777	0.996518	0.797287	0.439405	0.25686	0.112865377	SUN2

Reviewer #2 (Comments to the Author):
The study entitled "Observational, Causal Relationship and Shared Genetic Basis Between Cholelithiasis and Gastroesophageal Reflux Disease: Evidence from a Cohort Study and Comprehensive Genetic Analysis" is a prospective cohort analyses demonstrated a significantly increased risk of GERD in individuals with cholelithiasis as well as a higher risk of cholelithiasis among GERD patients. The observational correlations were analyzed using data from the UK Biobank prospective cohort study. Subsequently, bidirectional MR analysis revealed a causal effect of genetic liability to cholelithiasis on the incidence of GERD and a causal effect of genetically predicted GERD on cholelithiasis. Additionally, cholelithiasis and GERD exhibited a strong genetic association, with cross-trait meta-analyses identifying five novel independent loci shared between the two conditions. Three shared genes, SUN2, CBY1, and JOSD1, were further identified as novel risk genes.
The study utilized a large sample size from the UK Biobank, comprising over 330,000 participants for both cholelithiasis and GERD. This large cohort enhances the statistical power and generalizability of the findings, reducing the potential for sampling bias and increasing the reliability of the results. The study further employed Mendelian randomization (MR) analysis to explore bidirectional causal relationships between cholelithiasis and GERD. This approach allows for the assessment of causality rather than mere correlation, providing stronger evidence for the genetic link between the two conditions. Through comprehensive genetic analyses, the study identified five novel independent loci and three shared genes (SUN2, CBY1, and JOSD1) associated with both cholelithiasis and GERD. These findings contribute to a deeper understanding of the genetic mechanisms underlying the relationship between the two diseases and offer potential targets for future research and therapeutic development. The current study has high translational potential.
Authors’ Response:
We sincerely thank the reviewer for the thorough evaluation of our manuscript and pointing out lots of strengths of our study, including: (1) using large sample size to enhance the statistical power and reliability of the results; (2) applying comprehensive genetic analyses to assess the causal correlation and identify novel variants and shared genes; (3) contributing to a deeper understanding of the genetic mechanisms; (4) offering potential targets for future research and therapeutic development; (5) high translational potential. According to your kind comments, we have revised our manuscript point-to-point as follows (marked in blue font) and highlighted the revised parts with tracked changes in the manuscript (Manuscript - Marked.docx).

However, following comments must be answered before publication:
1. Provide the scatter plot or any other equivalent visualization for MR analysis
2. Sensitivity analysis also must be plotted for better understanding and visualization
3. Important SNPs and OR should be visualized with or without age and sex adjustments
Authors’ Response:
Thank you for your detailed and helpful comments. The relevant plots and tables we supplemented are shown below.
“The leave-one-out analysis suggested that the observed causal relationship was not influenced by any outliers (Supplementary Figures 2-3). The scatter plots, forest plots, and funnel plots of the MR results were displayed in Supplementary Figures 2-3.” (Page 15, Line 357-360).”
Besides, the important SNPs and OR are listed in Table 3, as shown below.
