# Supplementary material for: Observational, causal relationship and shared genetic basis between cholelithiasis and gastroesophageal reflux disease: evidence from a cohort study and comprehensive genetic analysis
Source: Gigascience. 2025 Mar 26;14:giaf023. doi: 10.1093/gigascience/giaf023 (PMC11943489; doi:10.1093/gigascience/giaf023)
Supplement: giaf023_Reviewer_1_Report_Original_Submission — Jian Zeng -- 7/9/2024 [file giaf023_reviewer_1_report_original_submission.pdf]

Reviewer Report

Title: Observational, Causal Relationship and Shared Genetic Basis Between Cholelithiasis and Gastroesophageal Reflux Disease: Evidence from a Cohort Study and Comprehensive Genetic Analysis

Version: Original Submission **Date:** 7/9/2024

Reviewer name: Jian Zeng

Reviewer Comments to Author:

Lyu and coworkers have investigated both observational and genetic links between a gallstone known as cholelithiasis and gastroesophageal reflux disease (GERD), leveraging UK Biobank datasets. The authors observed a significant, modest positive genetic correlation between these two traits. In addition, authors have identified some shared loci between two traits via cross-trait meta-analysis. The causal relationship was assessed by Mendelian randomisation (MR). Gene imputation methods TWAS and SMR were also performed, showing shared genes between two traits. Although the presented manuscript is an interesting premise for study, the biological implications of the results underlying cholelithiasis and GERD are lacking.

Some comments are provided to consider:

* Page 14. "genetically predicted GERD could increase the risk of cholelithiasis by 15% (OR = 1.15, 95%CI = 1.02-1.30, P = 0.025) according to IVW method (Figure 2B)". This is likely cherry picking, as this association signal is not picked up by the other methods.
* The genetic correlation using LDSC and/or the local correlation-based method p-HESS reported in this study seems modest. The liability scale of reported heritability (presented in Table 2) for both cholelithiasis and GERD was not shown. The local genetic correlation method identified eight correlated genomic loci between two traits. How was the MHC region (the most complicated genomic region) handled? Did the authors use LD-based clumping? Further discussion is necessary about genetic correlation and to what extent these correlated genetic factors might influence these two traits.
* Regarding the causality test by MR, I wonder how the confounders of these two traits were considered. Are there other sources of pleiotropy in the selected variants, as instruments can act via different pathways such as obesity, smoking, or dietary preferences? Is there any evidence to support that the confounders were minimised by the selection of instruments?
* According to the author's statement in the introduction (page 6), causality can be reversed. While doing MR analyses, did the authors perform reverse MR? The authors mentioned bidirectional causal relations were detected (in the conclusion section), but that was not clear from reading the method or results section. According to supplementary Table 3, a two-way causal relationship between cholelithiasis and GERD was observed. However, a sensitivity analysis revealed no significant causal association between them. This aspect of the results should be acknowledged in the results and discussion section.
* Regarding transcriptomic imputation-based analyses (TWAS and SMR), I did not see a follow-up colocalization test was performed. Is there any evidence to explain that the same variant is sufficient to influence gene expression and traits as predicted through TWAS?

* I would encourage authors to avoid using the term 'causal' (as emphasised in the conclusion section). Still larger datasets in the future relevant to cholelithiasis and GERD will be required to establish the causality between the two investigated traits. Furthermore, the conclusion section claims that biological mechanisms were explored between these two traits. There are plenty of bioinformatics analyses, such as biological pathway analyses, gene ontology, and biomolecular network analyses, which might be employed to support that conclusion. Unfortunately, I note this is the very weak part of the current manuscript; it lacks a thorough presentation of the biological implications of the findings underpinning cholelithiasis and GERD. Therefore, instead of presenting a series of statistical analyses, describing to what extent and how the presented results can explain the biology of the disease and its aetiology will strengthen the manuscript.
* I also believe that the transcriptomic imputation-based findings (TWAS, SMR) would also need to be validated, as these methods impute gene expression using GWAS and eQTLs. These eQTL datasets were obtained from normal individuals; therefore, it is uncertain whether the predicted gene expression will correlate with real gene expression in cholelithiasis and GERD patients. Are there any existing gene expression datasets, such as microarrays or RNA sequencing datasets, available for cholelithiasis and GERD? If so, authors could look at how predicted expression correlates with differential gene expression in cholelithiasis and GERD patients compared to controls.

Methods

Are the methods appropriate to the aims of the study, are they well described, and are necessary controls included? Choose an item.

Conclusions

Are the conclusions adequately supported by the data shown? Choose an item.

Reporting Standards

Does the manuscript adhere to the journal's guidelines on [minimum standards of reporting?]() Choose an item.

Choose an item.

Statistics

Are you able to assess all statistics in the manuscript, including the appropriateness of statistical tests used? Choose an item.

Quality of Written English

Please indicate the quality of language in the manuscript: Choose an item.

Declaration of Competing Interests

Please complete a declaration of competing interests, considering the following questions:

- Have you in the past five years received reimbursements, fees, funding, or salary from an organisation that may in any way gain or lose financially from the publication of this manuscript, either now or in the future?
- Do you hold any stocks or shares in an organisation that may in any way gain or lose financially from the publication of this manuscript, either now or in the future?
- Do you hold or are you currently applying for any patents relating to the content of the manuscript?
- Have you received reimbursements, fees, funding, or salary from an organization that holds or has applied for patents relating to the content of the manuscript?
- Do you have any other financial competing interests?
- Do you have any non-financial competing interests in relation to this paper?

If you can answer no to all of the above, write 'I declare that I have no competing interests' below. If your reply is yes to any, please give details below.

I declare that I have no competing interests

I agree to the open peer review policy of the journal. I understand that my name will be included on my report to the authors and, if the manuscript is accepted for publication, my named report including any attachments I upload will be posted on the website along with the authors' responses. I agree for my report to be made available under an Open Access Creative Commons CC-BY license (http://creativecommons.org/licenses/by/4.0/). I understand that any comments which I do not wish to be included in my named report can be included as confidential comments to the editors, which will not be published.

Choose an item.

To further support our reviewers, we have joined with Publons, where you can gain additional credit to further highlight your hard work (see: https://publons.com/journal/530/gigascience). On publication of this paper, your review will be automatically added to Publons, you can then choose whether or not to claim your Publons credit. I understand this statement.

Yes Choose an item.
