# Supplementary material for: Observational, causal relationship and shared genetic basis between cholelithiasis and gastroesophageal reflux disease: evidence from a cohort study and comprehensive genetic analysis
Source: Gigascience. 2025 Mar 26;14:giaf023. doi: 10.1093/gigascience/giaf023 (PMC11943489; doi:10.1093/gigascience/giaf023)
Supplement: giaf023_Reviewer_2_Report_Original_Submission — Mangala Hegde -- 8/12/2024 [file giaf023_reviewer_2_report_original_submission.pdf]

Reviewer Report

Title: Observational, Causal Relationship and Shared Genetic Basis Between Cholelithiasis and Gastroesophageal Reflux Disease: Evidence from a Cohort Study and Comprehensive Genetic Analysis

Version: Original Submission **Date:** 8/12/2024

Reviewer name: Mangala Hegde

Reviewer Comments to Author:

The study entitled "Observational, Causal Relationship and Shared Genetic Basis Between Cholelithiasis and Gastroesophageal Reflux Disease: Evidence from a Cohort Study and Comprehensive Genetic Analysis" is a prospective cohort analyses demonstrated a significantly increased risk of GERD in individuals with cholelithiasis as well as a higher risk of cholelithiasis among GERD patients. The observational correlations were analyzed using data from the UK Biobank prospective cohort study. Subsequently, bidirectional MR analysis revealed a causal effect of genetic liability to cholelithiasis on the incidence of GERD and a causal effect of genetically predicted GERD on cholelithiasis. Additionally, cholelithiasis and GERD exhibited a strong genetic association, with cross-trait meta-analyses identifying five novel independent loci shared between the two conditions. Three shared genes, SUN2, CBY1, and JOSD1, were further identified as novel risk genes.
The study utilized a large sample size from the UK Biobank, comprising over 330,000 participants for both cholelithiasis and GERD. This large cohort enhances the statistical power and generalizability of the findings, reducing the potential for sampling bias and increasing the reliability of the results. The study further employed Mendelian randomization (MR) analysis to explore bidirectional causal relationships between cholelithiasis and GERD. This approach allows for the assessment of causality rather than mere correlation, providing stronger evidence for the genetic link between the two conditions. Through comprehensive genetic analyses, the study identified five novel independent loci and three shared genes (SUN2, CBY1, and JOSD1) associated with both cholelithiasis and GERD. These findings contribute to a deeper understanding of the genetic mechanisms underlying the relationship between the two diseases and offer potential targets for future research and therapeutic development. The current study has high translational potential.
However, following comments must be answered before publication:
1. Provide the scatter plot or any other equivalent visualization for MR analysis
2. Sensitivity analysis also must be plotted for better understanding and visualization
3. Important SNPs and OR should be visualized with or without age and sex adjustments

Methods

Are the methods appropriate to the aims of the study, are they well described, and are necessary controls included? Choose an item.

Conclusions

Are the conclusions adequately supported by the data shown? Choose an item.

Reporting Standards

Does the manuscript adhere to the journal's guidelines on minimum standards of reporting? Choose an item.

Choose an item.

Statistics

Are you able to assess all statistics in the manuscript, including the appropriateness of statistical tests used? Choose an item.

Quality of Written English

Please indicate the quality of language in the manuscript: Choose an item.

Declaration of Competing Interests

Please complete a declaration of competing interests, considering the following questions:

- Have you in the past five years received reimbursements, fees, funding, or salary from an organisation that may in any way gain or lose financially from the publication of this manuscript, either now or in the future?
- Do you hold any stocks or shares in an organisation that may in any way gain or lose financially from the publication of this manuscript, either now or in the future?
- Do you hold or are you currently applying for any patents relating to the content of the manuscript?
- Have you received reimbursements, fees, funding, or salary from an organization that holds or has applied for patents relating to the content of the manuscript?
- Do you have any other financial competing interests?
- Do you have any non-financial competing interests in relation to this paper?

If you can answer no to all of the above, write 'I declare that I have no competing interests' below. If your reply is yes to any, please give details below.

1. No 2. No, t 3. No, I do not hold or are you currently applying for any patents relating to the content of the manuscript 4. No 5. I do not have any other financial competing interests 6. I do not have any non-financial competing interests in relation to this paper

I agree to the open peer review policy of the journal. I understand that my name will be included on my report to the authors and, if the manuscript is accepted for publication, my named report including any attachments I upload will be posted on the website along with the authors' responses. I agree for my

report to be made available under an Open Access Creative Commons CC-BY license (http://creativecommons.org/licenses/by/4.0/). I understand that any comments which I do not wish to be included in my named report can be included as confidential comments to the editors, which will not be published.

Choose an item.

To further support our reviewers, we have joined with Publons, where you can gain additional credit to further highlight your hard work (see: https://publons.com/journal/530/gigascience). On publication of this paper, your review will be automatically added to Publons, you can then choose whether or not to claim your Publons credit. I understand this statement.

Yes Choose an item.
